# Synthesis, Biological Evaluation and Structure–Activity Relationship of Juglone Derived Naphthoquinones as Potential Antipsoriatic Agents

**DOI:** 10.3390/biom16060802

**Published:** 2026-05-29

**Authors:** Tong Bu, Zile Gong, Yudong Ma, Lixia Dai, Yuchao Ma, Xiaoyan Yu, Xiaorong Yang, Xiaolou Miao, Xiaofei Shang

**Affiliations:** 1 College of Pharmacy, Gansu University of Chinese Medicine, Lanzhou 730000, China; butong@gszy.edu.cn (T.B.); gongzl1226@gszy.edu.cn (Z.G.); 2 Key Laboratory of New Animal Drug Project, Gansu Province, Key Laboratory of Veterinary Pharmaceutical Development of Ministry of Agriculture and Rural Affairs, Lanzhou Institute of Husbandry and Pharmaceutical Sciences, Chinese Academy of Agricultural Sciences, Lanzhou 730050, China; 821012520053@caas.cn (Y.M.); 1073323010065@st.gsau.edu.cn (L.D.); ma15203559424@gmail.com (Y.M.); yuabigail814@gmail.com (X.Y.); yangxiaorong01@caas.cn (X.Y.); miaoxiaolou@caas.cn (X.M.)

**Keywords:** naphthoquinones, structure–activity relationship, anti-inflammatory activity, psoriasis

## Abstract

Psoriasis is a chronic, immune-mediated inflammatory skin disease for which the development of structurally novel and accessible small-molecule candidates remains of considerable interest. In this study, a series of juglone-derived naphthoquinone analogs was synthesized to explore the influence of substitution pattern on anti-inflammatory activity and cytotoxicity. Their biological profiles were first evaluated in LPS-stimulated HaCaT cells by combining cytotoxicity assessment with nitric oxide (NO) screening. Most derivatives showed reduced cytotoxicity compared with juglone, and preliminary structure–activity relationship analysis indicated that retention of a free hydroxyl group at the C-2 position was generally favorable for both reduction in NO release and cellular safety, whereas C-3 alkyl substitution tended to weaken activity and increase cytotoxicity. Among the tested compounds, compound **11** showed the most favorable balance between reduction in NO release and low cytotoxicity. Further evaluation showed that compound **11** reduced the protein levels of several inflammatory mediators in the culture supernatants of LPS-stimulated HaCaT cells, including TNF-α, IL-6, IL-1β, IL-17A, and IL-23, under the tested conditions. In an imiquimod-induced psoriasis-like mouse model, topical administration of compound **11** partially alleviated IMQ-induced psoriasis-like skin lesions, improved histopathological changes to some extent, and reduced selected inflammatory cytokine levels in serum and skin tissues under the tested conditions. Exploratory target prediction, molecular docking, and in silico ADMET analyses provided supportive computational insight into the biological profile of compound **11**. Overall, these findings suggest that juglone-derived naphthoquinones may serve as useful natural-product-inspired scaffolds for further anti-inflammatory optimization, and compound **11** warrants further investigation in psoriasis-related experimental models.

## 1. Introduction

Psoriasis is a chronic, immune-mediated inflammatory skin disease characterized by keratinocyte hyperproliferation, aberrant differentiation, and persistent cutaneous inflammation, typically presenting as erythematous plaques with silvery scales [1,2,3]. Current therapeutic strategies include topical corticosteroids, vitamin D analogs, retinoids, conventional systemic agents, and biologics targeting key inflammatory cytokines such as TNF-α, IL-17, and IL-23 [1,2]. These treatments have substantially improved disease management; however, long-term treatment may still be associated with issues such as recurrence after discontinuation, treatment cost, accessibility, and adverse effects in some patients [1,4,5]. Therefore, the identification of structurally diverse small molecules with anti-inflammatory activity remains useful as a complementary direction for early-stage psoriasis-related drug discovery.

Natural products and their derivatives have long played an important role in drug discovery and development because of their broad structural diversity, biological relevance, and rich pharmacological activities [4,5,6]. Many clinically useful agents have originated from, or been inspired by, natural-product scaffolds, highlighting their continuing value as sources of bioactive molecular frameworks for medicinal chemistry optimization [6]. Nevertheless, the pharmacological potential of natural-product-derived compounds depends on their specific chemical structures and biological profiles, rather than on their natural origin alone. Some natural scaffolds may also show cytotoxicity, redox activity, poor selectivity, or other liabilities. In recent years, natural compounds have also attracted increasing attention in inflammatory disease research, including psoriasis, owing to their capacity to modulate multiple biological pathways and to provide structurally novel starting points for the development of new therapeutic candidates [4,5].

Among such scaffolds, quinone-containing natural products have been extensively investigated because of their rich bioactivity profiles and synthetic tractability [7,8,9]. Juglone (5-hydroxy-1,4-naphthoquinone), a naturally occurring naphthoquinone predominantly found in *Juglans* species, has attracted sustained medicinal chemistry interest because of its diverse biological activities, including anticancer, antimicrobial, immunomodulatory, and anti-inflammatory effects [7,8]. At the same time, the intrinsic electrophilicity and cytotoxicity of the parent scaffold may restrict its direct therapeutic use [7]. This limitation is closely related to the quinone moiety, which can participate in redox cycling and electrophilic reactions with cellular nucleophiles. These chemical properties may contribute to biological activity, but they may also lead to nonspecific oxidative stress, protein modification, and cytotoxicity. Therefore, structural modification of the juglone scaffold is needed to explore whether anti-inflammatory activity can be retained while reducing nonspecific cytotoxicity.

Importantly, recent medicinal chemistry studies have shown that structural modification of juglone can substantially improve or redirect its biological activity. For example, juglone derivatives have been developed as potent SARS-CoV-2 main protease inhibitors [10], and 2-/3-substituted juglone derivatives have also been optimized into dual antiplatelet/anticancer agents with improved activity or selectivity relative to juglone itself [11]. These studies illustrate that judicious modification of the juglone quinone core can markedly reshape biological performance. However, to the best of our knowledge, systematic psoriasis-oriented medicinal chemistry studies centered on juglone-derived naphthoquinones remain limited.

In the present work, we used juglone as a natural-product-inspired starting scaffold to construct a small series of structurally diversified naphthoquinone derivatives. Our structural modification strategy focused on modulating substituent patterns around the quinone core, particularly by comparing analogs with or without a free hydroxyl group and by examining the effect of additional alkyl substitution on selected 2-hydroxynaphthoquinone cores. Accordingly, a series of juglone-derived naphthoquinones was synthesized and evaluated, followed by cytotoxicity assessment and preliminary anti-inflammatory evaluation in LPS-stimulated HaCaT cells. HaCaT keratinocytes were selected because keratinocyte activation is directly involved in psoriatic inflammation; therefore, NO production in this model was used as an initial screening readout of keratinocyte-associated inflammatory responses rather than as a macrophage-specific NO model. On the basis of the activity and cytotoxicity profiles, a representative compound was further examined in cytokine assays and in an imiquimod (IMQ)-induced psoriasis-like mouse model. In addition, structure–activity relationships (SAR) within this series were analyzed, and exploratory computational studies were performed to provide auxiliary insight into possible target interactions and ADMET-related properties.

## 2. Materials and Methods

### 2.1. Chemistry

All final compounds used for biological evaluation were purified by silica gel column chromatography and/or recrystallization. Their chemical structures were confirmed by ^1^H NMR, ^13^C NMR, and HRMS. The apparent purity of all isolated compounds was preliminarily assessed by TLC and ^1^H NMR spectroscopy, and no obvious impurity signals were observed in the ^1^H NMR spectra. The corresponding NMR spectra are provided in the Supplementary Materials.

To further address quantitative purity assessment for the key active compounds, representative biologically active compounds **4** and **11** were analyzed by analytical HPLC using UV detection. Compound **11** was selected because it was the lead compound used for further cytokine assays, in vivo evaluation, docking, and ADMET analysis, whereas compound **4** was selected as a representative active derivative from the preliminary NO-screening assay. Both compounds showed purities greater than 95% based on HPLC peak-area normalization.

The HPLC analytical conditions, retention times, and purity values are provided in the Supplementary Materials. Analytical HPLC purity analysis was performed using a C18 reversed-phase column with UV detection at 254 nm. The mobile phase consisted of acetonitrile/water, the flow rate was 1.0 mL/min, and the injection volume was 10 μL. Compound purity was calculated by peak-area normalization.

Compounds **1** and **3**–**11** were identified as previously reported compounds and were synthesized according to reported or modified literature procedures, as indicated by the corresponding references. Compounds **2** and **12**–**26** were not found as exact literature matches during our literature search and are therefore reported here as newly synthesized derivatives. These new derivatives were prepared using modified literature procedures for aminonaphthoquinone formation and *L*-proline-catalyzed alkylation of hydroxynaphthoquinone scaffolds, respectively [12]. Unless otherwise specified, all chemical reagents and solvents were purchased from commercial suppliers, including Aladdin Reagent Co., Ltd. (Shanghai, China), Macklin Biochemical Co., Ltd. (Shanghai, China), Energy Chemical (Shanghai, China), and Bidepharm (Shanghai, China), and were used without further purification.

**5-(allyloxy)naphthalene-1,4-dione (1)** [13]. Allyl bromide (1.16 mmol, 2 equiv) was added to a solution of juglone (0.58 mmol, 1 eq) and silver oxide (0.87 mmol, 1.5 eq) in DCM (5.0 mL). The mixed solution was stirred at room temperature for 20 h. Then, silver oxide (0.58 mmol, 1 eq) and allyl bromide (0.52 mmol, 0.9 eq) were added to the reaction system, and stirring was continued for 15 h. After TLC indicated consumption of the starting material, the mixture was filtered, and the filter cake was washed with DCM. The combined organic layers were washed with water and saturated brine, dried over anhydrous Na_2_SO_4_, filtered, and concentrated under reduced pressure. The residue was purified by silica gel column chromatography with petroleum ether/EtOAc (4:1) to afford the product. Yellow solid, 58% yield, mp 95 ± 1 °C. ^1^H NMR (500 MHz, Chloroform-*d*) δ 7.72 (dd, *J* = 7.6, 1.2 Hz, 1H), 7.68–7.62 (m, 1H), 7.29 (dd, *J* = 8.3, 1.1 Hz, 1H), 6.87 (d, *J* = 2.2 Hz, 2H), 6.15–6.05 (m, 1H), 5.66 (dq, *J* = 17.2, 1.7 Hz, 1H), 5.37 (dq, *J* = 10.6, 1.5 Hz, 1H), 4.73 (dt, *J* = 4.7, 1.8 Hz, 2H). ^13^C NMR (126 MHz, Chloroform-*d*) δ 185.35, 184.27, 158.69, 141.01, 136.29, 134.90, 134.19, 132.06, 120.14, 119.54, 119.45, 118.18, 69.91. HRMS (ESI): calcd for C_13_H_10_O_3_H (M + H^+^) 215.07022; found 215.07005.

**5-(allyloxy)-2-aminonaphthalene-1,4-dione (2)** [14]. To a solution of *O*-Benzylhydroxylamine hydrochloride (0.50 mmol, 1 eq) in ethanol (4.0 mL) was added triethylamine (0.50 mmol, 1 eq) at 5 °C. The mixed solution was stirred at 5 °C for 10 min. Then, a solution of **1** (0.75 mmol, 1.5 eq) in ethanol (3 mL) was added dropwise to the reaction system, and stirring was continued for 5 h at room temperature. After TLC indicated consumption of the starting material, the solvent was removed under reduced pressure. The residue was purified by silica gel column chromatography with petroleum ether/EtOAc (4:1) to afford the product. Orange solid, 60% yield, mp 184 ± 1 °C. ^1^H NMR (500 MHz, DMSO-*d*_6_) δ 7.71 (dd, *J* = 8.4, 7.6 Hz, 1H), 7.54 (dd, *J* = 7.6, 1.1 Hz, 1H), 7.36 (dd, *J* = 8.6, 1.1 Hz, 1H), 6.12–6.02 (m, 1H), 5.74 (s, 1H), 5.71–5.64 (m, 1H), 5.30 (dq, *J* = 10.7, 1.8 Hz, 1H), 4.71 (dt, *J* = 4.0, 1.8 Hz, 2H). ^13^C NMR (126 MHz, DMSO-*d*_6_) δ 181.20, 179.92, 158.29, 151.47, 135.74, 135.57, 132.90, 118.05, 117.84, 117.69, 116.94, 100.53, 68.97. HRMS (ESI): calcd for C_13_H_11_NO_3_H (M + H^+^) 230.08113; found 230.08092.

**5-methoxynaphthalene-1,4-dione (3)** [13]. To a solution of juglone (0.58 mmol, 1 eq) and silver oxide (0.87 mmol, 1.5 eq) in DCM (5.0 mL) was added iodomethane (1.16 mmol, 2 eq). The mixed solution was stirred at room temperature for 20 h. Then, silver oxide (0.58 mmol, 1 eq) and iodomethane (0.52 mmol, 0.9 eq) were added to the reaction system, and stirring was continued for 15 h. After TLC indicated consumption of the starting material, the mixture was filtered, and the filter cake was washed with DCM. The combined organic layers were washed with water and saturated brine, dried over anhydrous Na_2_SO_4_, filtered, and concentrated under reduced pressure. The residue was purified by silica gel column chromatography with petroleum ether/EtOAc (4:1) to afford the product. Yellow solid, 60% yield, mp 188 ± 1 °C. ^1^H NMR (500 MHz, Chloroform-*d*) δ 7.72 (dd, *J* = 7.7, 1.5 Hz, 1H), 7.69 (t, *J* = 7.9 Hz, 1H), 7.32 (dd, *J* = 8.2, 1.5 Hz, 1H), 6.87 (d, *J* = 1.5 Hz, 2H), 4.01 (s, 3H). ^13^C NMR (126 MHz, Chloroform-*d*) δ 185.33, 184.47, 159.76, 141.01, 136.33, 135.12, 134.17, 119.85, 119.30, 118.07, 56.61. HRMS (ESI): calcd for C_11_H_8_O_3_H (M + H^+^) 189.05455; found 189.05455.

**8-methoxy-2-(phenylamino)naphthalene-1,4-dione (4)** [15]. To a solution of **3** (4.25 mmol, 1 eq) in ethanol (20.0 mL) was added aniline (85.00 mmol, 20 eq) at 0 °C. The mixed solution was stirred at room temperature for 5 days. After TLC indicated consumption of the starting material, the reaction mixture was acidified dropwise with 6 M HCl. The resulting red precipitate was collected by filtration, washed, and dried to afford the product. Red solid, 64% yield, mp 151 ± 1 °C. ^1^H NMR (500 MHz, DMSO-*d*_6_) δ 9.08 (s, 1H), 7.77 (dd, *J* = 8.5, 7.6 Hz, 1H), 7.56 (dd, *J* = 7.6, 1.1 Hz, 1H), 7.45 (dd, *J* = 4.1, 3.0 Hz, 1H), 7.44–7.41 (m, 2H), 7.38–7.35 (m, 2H), 7.21 (tt, *J* = 7.2, 1.2 Hz, 1H), 6.01 (s, 1H), 3.94 (s, 3H). ^13^C NMR (126 MHz, DMSO-*d*_6_) δ 182.13, 179.39, 159.70, 147.08, 138.19, 136.08, 134.78, 129.68, 129.28, 125.17, 123.60, 122.58, 117.87, 117.70, 117.21, 100.29, 56.41. HRMS (ESI): calcd for C_17_H_13_NO_3_H (M + H^+^) 280.09676; found 280.09662.

**2-amino-5-hydroxynaphthalene-1,4-dione (5)** [14]. To a solution of *O*-Benzylhydroxylamine hydrochloride (1.00 mmol, 1 eq) in ethanol (4.0 mL) was added triethylamine (1.00 mmol, 1 eq) at 5 °C. The mixed solution was stirred at 5 °C for 10 min. Then, a solution of juglone (1.50 mmol, 1.5 eq) in ethanol (3 mL) was added dropwise to the reaction system, and stirring was continued for 5 h at room temperature. After TLC indicated consumption of the starting material, the solvent was removed under reduced pressure. The residue was purified by silica gel column chromatography with petroleum ether/EtOAc (4:1) to afford the product. Red solid, 44% yield, mp 268 ± 1 °C. ^1^H NMR (500 MHz, DMSO-*d*_6_) δ 13.35 (s, 1H), 7.59–7.53 (m, 1H), 7.48 (dd, *J* = 7.5, 1.2 Hz, 1H), 7.25 (dd, *J* = 8.3, 1.1 Hz, 1H), 5.74 (s, 1H). ^13^C NMR (126 MHz, DMSO-*d*_6_) δ 188.33, 181.18, 160.07, 151.81, 134.12, 130.50, 125.07, 118.35, 114.48, 100.73. HRMS (ESI): calcd for C_10_H_6_NO_3_ (M-H^+^) 188.03468; found 188.03528.

**2,5-dihydroxynaphthalene-1,4-dione (6)** [16]. **5** (0.5 mmol) was added to concentrated hydrochloric acid (25 mL), and the mixture was refluxed with stirring for 24 h. After TLC indicated consumption of the starting material, the mixture was cooled to room temperature and filtered. The filtrate was extracted with DCM, and the combined organic layers were washed with water and saturated brine, dried over anhydrous Na_2_SO_4_, filtered, and concentrated under reduced pressure. The residue was purified by silica gel column chromatography with petroleum ether/EtOAc (1:1) to afford the product. Yellow solid, Yield: 31%; mp 211 ± 1 °C. ^1^H NMR (500 MHz, DMSO-*d*_6_) δ 12.47 (s, 1H), 7.70–7.62 (m, 1H), 7.53 (dd, *J* = 7.5, 1.3 Hz, 1H), 7.31 (dd, *J* = 8.4, 1.3 Hz, 1H), 6.12 (s, 1H). ^13^C NMR (126 MHz, DMSO-*d*_6_) δ 191.44, 180.65, 160.74, 159.95, 135.43, 130.81, 124.69, 118.72, 113.96, 110.48. HRMS (ESI): calcd for C_10_H_5_O_4_ (M-H^+^) 189.01929; found 189.01820.

**8-hydroxy-2-(*p*-tolylthio)naphthalene-1,4-dione (7)** [17]. Juglone (3.00 mmol, 1 eq) was dissolved in ethanol (25.0 mL). To this solution was slowly added a solution of *p*-toluenethiol (3.00 mmol, 1 eq) in ethanol (6 mL) at 0 °C. The resulting mixture was then stirred at room temperature for 4 h. After TLC indicated consumption of the starting material, the reaction mixture was cooled in an ice bath. The resulting precipitate was collected by filtration, washed with cold ethanol, and dried under vacuum to afford the product. Yellow solid, 26% yield, mp 170 ± 1 °C. ^1^H NMR (500 MHz, Chloroform-*d*) δ 11.72 (s, 1H), 7.61 (dd, *J* = 8.4, 7.4 Hz, 1H), 7.55 (dd, *J* = 7.5, 1.2 Hz, 1H), 7.43–7.38 (m, 2H), 7.31 (d, *J* = 7.9 Hz, 2H), 7.23 (dd, *J* = 8.4, 1.2 Hz, 1H), 6.07 (s, 1H), 2.43 (s, 3H). ^13^C NMR (126 MHz, Chloroform-*d*) δ 187.36, 181.41, 161.98, 156.92, 141.35, 137.22, 135.73, 132.39, 131.40, 129.04, 123.92, 123.38, 119.45, 114.79, 21.53. HRMS (ESI): calcd for C_17_H_12_O_3_SH (M + H^+^) 297.05797; found 297.05801.

**2,8-dihydroxynaphthalene-1,4-dione (8)** [18]. To a solution of **7** (3.00 mmol, 1 eq) in ethanol (48.0 mL) was added sodium hydroxide solution (2 M, 24.0 mL) at room temperature. The mixed solution was refluxed with stirring for 3 h. After TLC indicated consumption of the starting material, the reaction mixture was cooled to 0 °C and acidified dropwise with 6M HCl. The resulting precipitate was collected by filtration, washed, and dried to afford the product. Yellow solid, 80% yield, mp 213 ± 1 °C. ^1^H NMR (500 MHz, Chloroform-*d*) δ 11.07 (s, 1H), 7.70–7.66 (m, 1H), 7.65 (dd, *J* = 7.4, 1.6 Hz, 1H), 7.24 (dd, *J* = 7.9, 1.7 Hz, 1H), 6.34 (s, 1H). ^13^C NMR (126 MHz, Chloroform-*d*) δ 185.34, 184.21, 161.77, 156.28, 138.36, 132.88, 123.57, 119.76, 113.29, 111.78. HRMS (ESI): calcd for C_10_H_6_O_4_H (M + H^+^) 189.01930; found 189.01853.

**2-hydroxy-5-methoxynaphthalene-1,4-dione (9)** [16]. Potassium *tert*-butoxide (5.00 mmol, 5 eq) was dissolved in *tert*-butanol (5 mL), and the solution was bubbled with oxygen for 30 min. Subsequently, 5-Methoxy-1-tetralone (1.00 mmol, 1 eq) was added to the reaction system, and the reaction mixture was stirred under an oxygen atmosphere for an additional 2 h at room temperature. After TLC indicated consumption of the starting material, the reaction mixture was cooled to 0 °C, and the pH was adjusted to 1 by adding 2M HCl. The mixture was extracted with DCM, and the combined organic phases were extracted with saturated aqueous NaHCO_3_ solution. The aqueous phase was then acidified to pH 1 with 2M HCl and extracted with DCM. The combined organic layers were washed with saturated brine, dried over anhydrous Na_2_SO_4_, filtered, and concentrated under reduced pressure. The resulting residue was recrystallized from dichloromethane/petroleum ether to afford the product. Yellow solid, 62% yield, mp 162 ± 1 °C. ^1^H NMR (500 MHz, DMSO-*d*_6_) δ 9.83 (s, 1H), 6.34 (dd, *J* = 8.4, 7.6 Hz, 1H), 6.24 (dd, *J* = 7.6, 1.1 Hz, 1H), 6.14 (dd, *J* = 8.5, 1.2 Hz, 1H), 4.63 (s, 1H), 2.50 (s, 3H). ^13^C NMR (126 MHz, DMSO-*d*_6_) δ 184.16, 181.60, 158.80, 156.92, 134.24, 132.69, 119.70, 118.75, 118.55, 113.29, 56.28. HRMS (ESI): calcd for C_11_H_8_O_4_H (M + H^+^) 205.04949; found 205.04953.

**2-hydroxy-6-methoxynaphthalene-1,4-dione (10)** [16]. Compound **10** was synthesized from 6-methoxy-1-tetralone using the same procedure as described for **9**. Yellow solid, 61% yield, mp 214 ± 1 °C. ^1^H NMR (500 MHz, DMSO-*d*_6_) δ 7.90 (d, *J* = 8.5 Hz, 1H), 7.30 (d, *J* = 2.6 Hz, 1H), 7.24 (dd, *J* = 8.6, 2.7 Hz, 1H), 6.11 (s, 1H), 3.90 (s, 3H). ^13^C NMR (126 MHz, DMSO-*d*_6_) δ 180.01, 164.14, 134.32, 128.74, 123.76, 118.76, 110.58, 109.76, 56.00. HRMS (ESI): calcd for C_11_H_8_O_4_H (M + H^+^) 205.04951; found 205.04926.

**2-hydroxy-8-methoxynaphthalene-1,4-dione (11)** [16]. Compound **11** was synthesized from 8-methoxy-1-tetralone using the same procedure as described for **9**. Yellow solid, 34% yield, mp 211 ± 1 °C. ^1^H NMR (500 MHz, Chloroform-*d*) δ 7.80–7.77 (m, 1H), 7.76–7.73 (m, 1H), 7.72 (s, 1H), 6.29 (s, 1H), 4.05 (s, 3H). ^13^C NMR (126 MHz, Chloroform-*d*) δ 184.85, 180.27, 160.55, 156.92, 136.97, 135.37, 119.70, 117.05, 108.69, 56.70. HRMS (ESI): calcd for C_11_H_8_O_4_H (M + H^+^) 205.04947; found 205.04950.

**3-butyl-2-hydroxy-5-methoxynaphthalene-1,4-dione (12).** To a solution of **9** (0.30 mmol, 1 eq) in dichloromethane (5.0 mL) was added *L*-Proline (0.09 mmol, 0.3 eq) and diethyl 1,4-dihydro-2,6-dimethyl-3,5- pyridinedicarboxylate (0.33 mmol, 1.1 eq) at room temperature. Then, butyraldehyde (0.90 mmol, 3.0 eq) was added to the reaction system, and stirring was continued for 5 h. After TLC indicated consumption of the starting material, the mixture was filtered, and the filter cake was washed with DCM. The combined organic layers were washed with water and saturated brine, dried over anhydrous Na_2_SO_4_, filtered, and concentrated under reduced pressure. The residue was purified by silica gel column chromatography with petroleum ether/EtOAc (32:1) to afford the product. Yellow solid, 60% yield, mp 133 ± 1 °C. ^1^H NMR (500 MHz, Chloroform-*d*) δ 7.75 (dd, *J* = 7.6, 1.1 Hz, 1H), 7.60 (dd, *J* = 8.5, 7.6 Hz, 1H), 7.32 (dd, *J* = 8.5, 1.2 Hz, 1H), 7.08–7.03 (m, 1H), 3.99 (s, 3H), 2.61–2.52 (m, 2H), 1.55–1.46 (m, 2H), 1.44–1.34 (m, 2H), 0.92 (t, *J* = 7.3 Hz, 3H). ^13^C NMR (126 MHz, Chloroform-*d*) δ 184.65, 181.85, 159.80, 151.37, 134.01, 131.86, 126.54, 120.07, 119.54, 119.10, 56.64, 30.63, 23.46, 23.10, 14.04. HRMS (ESI): calcd for C_15_H_16_O_4_H (M + H^+^) 261.11212; found 261.11213.

**3-decyl-2-hydroxy-5-methoxynaphthalene-1,4-dione (13)**. Compound **13** was synthesized from decanal using the same procedure as described for **12**. Yellow solid, 58% yield, mp 99 ± 1 °C. ^1^H NMR (500 MHz, Chloroform-*d*) δ 7.75 (dd, *J* = 7.5, 1.1 Hz, 1H), 7.64–7.58 (m, 1H), 7.33 (d, *J* = 1.1 Hz, 1H), 7.05 (s, 1H), 4.00 (s, 3H), 2.59–2.53 (m, 2H), 1.56–1.46 (m, 2H), 1.30–1.20 (m, 14H), 0.90–0.84 (m, 3H). ^13^C NMR (126 MHz, Chloroform-*d*) δ 184.64, 181.86, 159.80, 151.35, 134.01, 131.87, 126.59, 120.09, 119.54, 119.11, 56.64, 32.05, 30.03, 29.81, 29.73, 29.61, 29.48, 28.55, 23.77, 22.83, 14.25. HRMS (ESI): calcd for C_21_H_27_O_4_ (M-H^+^) 345.20592; found 345.20698.

**3-hexadecyl-2-hydroxy-5-methoxynaphthalene-1,4-dione (14)**. Compound **14** was synthesized from palmitaldehyde using the same procedure as described for **12**. Yellow solid, 34% yield, mp 98 ± 1 °C. ^1^H NMR (500 MHz, Chloroform-*d*) δ 7.74 (dd, *J* = 7.6, 1.1 Hz, 1H), 7.63–7.56 (m, 1H), 7.31 (dd, *J* = 8.5, 1.1 Hz, 1H), 7.09 (s, 1H), 3.99 (s, 3H), 2.59–2.52 (m, 2H), 1.56–1.46 (m, 2H), 1.30–1.19 (m, 26H), 0.90–0.82 (m, 3H). ^13^C NMR (126 MHz, Chloroform-*d*) δ 184.63, 181.84, 159.77, 151.36, 133.98, 131.86, 126.59, 120.06, 119.51, 119.08, 56.61, 32.05, 30.03, 29.83, 29.73, 29.60, 29.49, 28.54, 23.75, 22.82, 14.24. HRMS (ESI): calcd for C_27_H_40_O_4_H (M + H^+^) 429.29989; found 429.30014.

**3-butyl-2-hydroxy-6-methoxynaphthalene-1,4-dione (15)**. To a solution of **10** (0.30 mmol, 1 eq) in dichloromethane (5.0 mL) was added *L*-Proline (0.09 mmol, 0.3 eq) and diethyl 1,4-dihydro-2,6-dimethyl-3,5-pyridinedicarboxylate (0.33 mmol, 1.1 eq) at room temperature. Then, butyraldehyde (0.90 mmol, 3.0 eq) was added to the reaction system, and stirring was continued for 5 h. After TLC indicated consumption of the starting material, the mixture was filtered, and the filter cake was washed with DCM. The combined organic layers were washed with water and saturated brine, dried over anhydrous Na_2_SO_4_, filtered, and concentrated under reduced pressure. The residue was purified by silica gel column chromatography with petroleum ether/EtOAc (32:1) to afford the product. Yellow solid, 55% yield, mp 141 ± 1 °C. ^1^H NMR (500 MHz, Chloroform-*d*) δ 8.01 (d, *J* = 8.6 Hz, 1H), 7.57 (d, *J* = 2.7 Hz, 1H), 7.41 (s, 1H), 7.11 (dd, *J* = 8.5, 2.6 Hz, 1H), 3.95 (s, 3H), 2.62–2.52 (m, 2H), 1.56–1.45 (m, 2H), 1.45–1.33 (m, 2H), 0.97–0.89 (m, 3H). ^13^C NMR (126 MHz, Chloroform-*d*) δ 184.81, 180.33, 165.30, 153.45, 135.65, 128.83, 124.09, 122.81, 119.09, 110.99, 56.10, 30.55, 23.20, 22.97, 14.06. HRMS (ESI): calcd for C_15_H_16_O_4_H (M + H^+^) 261.11208; found 261.11207.

**3-decyl-2-hydroxy-6-methoxynaphthalene-1,4-dione (16)**. Compound **16** was synthesized from decanal using the same procedure as described for **15**. Yellow solid, 37% yield, mp 111 ± 1 °C. ^1^H NMR (400 MHz, Chloroform-*d*) δ 8.00 (d, *J* = 8.5 Hz, 1H), 7.57 (d, *J* = 2.6 Hz, 1H), 7.42 (s, 1H), 7.10 (dd, *J* = 8.6, 2.6 Hz, 1H), 3.94 (s, 3H), 2.60–2.52 (m, 2H), 1.56–1.46 (m, 2H), 1.30–1.20 (m, 14H), 0.90–0.82 (m, 3H). ^13^C NMR (126 MHz, Chloroform-*d*) δ 184.80, 180.34, 165.29, 153.43, 135.66, 128.82, 124.14, 122.82, 119.09, 110.99, 56.10, 32.04, 29.90, 29.74, 29.61, 29.47, 28.43, 23.49, 22.82, 14.25. HRMS (ESI): calcd for C_21_H_28_O_4_H (M + H^+^) 345.20598; found 345.20598.

**3-hexadecyl-2-hydroxy-6-methoxynaphthalene-1,4-dione (17)**. Compound **17** was synthesized from palmitaldehyde using the same procedure as described for **15**. Yellow solid, 36% yield, mp 110 ± 1 °C. ^1^H NMR (400 MHz, Chloroform-*d*) δ 8.01 (d, *J* = 8.5 Hz, 1H), 7.57 (d, *J* = 2.6 Hz, 1H), 7.41 (s, 1H), 7.11 (dd, *J* = 8.5, 2.6 Hz, 1H), 3.95 (s, 3H), 2.60–2.52 (m, 2H), 1.56–1.46 (m, 2H), 1.30–1.19 (m, 26H), 0.90–0.84 (m, 3H). ^13^C NMR (101 MHz, Chloroform-*d*) δ 184.80, 180.34, 165.30, 153.42, 135.66, 128.83, 124.13, 122.82, 119.10, 110.99, 56.11, 32.07, 29.91, 29.84, 29.73, 29.62, 29.51, 28.44, 23.50, 22.84, 14.27. HRMS (ESI): calcd for C_27_H_40_O_4_H (M + H^+^) 429.29988; found 429.30006.

**2-butyl-3-hydroxy-5-methoxynaphthalene-1,4-dione (18)**. To a solution of **11** (0.30 mmol, 1 eq) in dichloromethane (5.0 mL) was added *L*-Proline (0.09 mmol, 0.3 eq) and diethyl 1,4-dihydro-2,6-dimethyl-3,5-pyridinedicarboxylate (0.33 mmol, 1.1 eq) at room temperature. Then, butyraldehyde (0.90 mmol, 3.0 eq) was added to the reaction system, and stirring was continued for 5 h. After TLC indicated consumption of the starting material, the mixture was filtered, and the filter cake was washed with DCM. The combined organic layers were washed with water and saturated brine, dried over anhydrous Na_2_SO_4_, filtered, and concentrated under reduced pressure. The residue was purified by silica gel column chromatography with petroleum ether/EtOAc (32:1) to afford the product. Yellow solid, 45% yield, mp 155 ± 1 °C. ^1^H NMR (500 MHz, Chloroform-*d*) δ 7.78 (dd, *J* = 7.6, 1.1 Hz, 1H), 7.68 (dd, *J* = 8.5, 7.5 Hz, 2H), 7.22 (dd, *J* = 8.5, 1.1 Hz, 1H), 4.02 (s, 3H), 2.59–2.52 (m, 2H), 1.55–1.45 (m, 2H), 1.43–1.33 (m, 2H), 0.96–0.88 (m, 3H). ^13^C NMR (126 MHz, Chloroform-*d*) δ 184.67, 180.08, 168.17, 160.12, 153.63, 144.88, 136.38, 135.39, 122.46, 119.80, 117.08, 116.74, 99.69, 56.64, 30.46, 24.92, 23.14, 22.98, 19.31, 14.05. HRMS (ESI): calcd for C_15_H_15_O_4_ (M-H^+^) 259.09751; found 259.09749.

**2-decyl-3-hydroxy-5-methoxynaphthalene-1,4-dione (19)**. Compound **19** was synthesized from decanal using the same procedure as described for **18**. Yellow solid, 25% yield, mp 114 ± 1 °C. ^1^H NMR (400 MHz, Chloroform-*d*) δ 7.79 (dd, *J* = 7.6, 1.1 Hz, 1H), 7.71–7.64 (m, 2H), 7.23 (dd, *J* = 8.6, 1.1 Hz, 1H), 4.02 (s, 3H), 2.58–2.50 (m, 2H), 1.56–1.46 (m, 2H), 1.30–1.20 (m, 14H), 0.90–0.82 (m, 3H). ^13^C NMR (101 MHz, Chloroform-*d*) δ 184.67, 180.10, 160.11, 153.61, 136.38, 135.39, 122.51, 119.81, 117.07, 116.73, 56.64, 32.03, 29.90, 29.74, 29.60, 29.46, 28.34, 23.42, 22.82, 14.26. HRMS (ESI): calcd for C_21_H_27_O_4_ (M-H^+^) 343.19115; found 343.19142.

**2-hexadecyl-3-hydroxy-5-methoxynaphthalene-1,4-dione (20)**. Compound **20** was synthesized from palmitaldehyde using the same procedure as described for **18**. Yellow solid, 24% yield, mp 96 ± 1 °C. ^1^H NMR (500 MHz, Chloroform-*d*) δ 7.78 (dd, *J* = 7.7, 1.1 Hz, 1H), 7.69 (d, *J* = 8.6 Hz, 2H), 7.22 (dd, *J* = 8.5, 1.0 Hz, 1H), 4.02 (s, 3H), 2.58–2.51 (m, 2H), 1.55–1.46 (m, 2H), 1.24 (m, 26H), 0.90–0.82 (m, 3H). ^13^C NMR (126 MHz, Chloroform-*d*) δ 184.65, 180.09, 160.11, 153.61, 136.36, 135.40, 122.51, 119.81, 117.09, 116.72, 56.63, 32.06, 29.91, 29.84, 29.73, 29.61, 29.50, 28.34, 23.42, 22.83, 14.25. HRMS (ESI): calcd for C_27_H_39_O_4_ (M-H^+^) 427.28533; found 427.28555.

**2-butyl-3,5-dihydroxynaphthalene-1,4-dione (21)**. To a solution of **8** (0.30 mmol, 1 eq) in dichloromethane (5.0 mL) was added *L*-Proline (0.09 mmol, 0.3 eq) and diethyl 1,4-dihydro-2,6-dimethyl-3,5-pyridinedicarboxylate (0.33 mmol, 1.1 eq) at room temperature. Then, butyraldehyde (0.90 mmol, 3.0 eq) was added to the reaction system, and stirring was continued for 5 h. After TLC indicated consumption of the starting material, the mixture was filtered, and the filter cake was washed with DCM. The combined organic layers were washed with water and saturated brine, dried over anhydrous Na_2_SO_4_, filtered, and concentrated under reduced pressure. The residue was purified by silica gel column chromatography with petroleum ether/EtOAc (32:1) to afford the product. Yellow solid, 41% yield, mp 116 ± 1 °C. ^1^H NMR (500 MHz, Chloroform-*d*) δ 11.11 (s, 1H), 7.66–7.58 (m, 2H), 7.22 (s, 1H), 7.18 (dd, *J* = 8.0, 1.6 Hz, 1H), 2.62–2.55 (m, 2H), 1.54–1.46 (m, 2H), 1.43–1.34 (m, 2H), 0.93 (t, *J* = 7.3 Hz, 3H). ^13^C NMR (126 MHz, Chloroform-*d*) δ 184.91, 184.03, 161.25, 152.81, 137.65, 132.90, 126.25, 123.20, 119.80, 113.14, 30.49, 23.32, 23.01, 14.03. HRMS (ESI): calcd for C_14_H_13_O_4_ (M-H^+^) 245.08187; found 245.08167.

**2-decyl-3,5-dihydroxynaphthalene-1,4-dione (22)**. Compound **22** was synthesized from decanal using the same procedure as described for **21**. Yellow solid, 38% yield, mp 110 ± 1 °C. ^1^H NMR (500 MHz, Chloroform-*d*) δ 11.11 (s, 1H), 7.66–7.57 (m, 2H), 7.24 (s, 1H), 7.18 (dd, *J* = 8.0, 1.6 Hz, 1H), 2.60–2.54 (m, 2H), 1.54–1.45 (m, 2H), 1.30–1.19 (m, 26H), 0.90–0.82 (m, 3H). ^13^C NMR (126 MHz, Chloroform-*d*) δ 184.92, 184.01, 161.24, 152.80, 137.62, 132.90, 126.30, 123.18, 119.79, 113.14, 32.04, 29.93, 29.75, 29.71, 29.59, 29.46, 28.38, 23.60, 22.82, 14.25. HRMS (ESI): calcd for C_20_H_25_O_4_ (M-H^+^) 329.17579; found 329.17563.

**2-hexadecyl-3,5-dihydroxynaphthalene-1,4-dione (23)**. Compound **23** was synthesized from palmitaldehyde using the same procedure as described for **21**. Yellow solid, 32% yield, mp 102 ± 1 °C. ^1^H NMR (500 MHz, Chloroform-*d*) δ 11.11 (s, 1H), 7.66–7.59 (m, 2H), 7.22 (s, 1H), 7.19 (dd, *J* = 8.0, 1.5 Hz, 1H), 2.62–2.53 (m, 2H), 1.56–1.46 (m, 2H), 1.30–1.19 (m, 26H), 0.90–0.84 (m, 3H). ^13^C NMR (126 MHz, Chloroform-*d*) δ 184.93, 184.01, 161.26, 152.78, 137.64, 132.92, 126.30, 123.19, 119.80, 113.15, 32.08, 29.94, 29.83, 29.72, 29.60, 29.51, 28.38, 23.61, 22.84, 14.26. HRMS (ESI): calcd for C_26_H_37_O_4_ (M-H^+^) 413.26971; found 413.26975.

**3-butyl-2,5-dihydroxynaphthalene-1,4-dione (24)**. To a solution of **6** (0.30 mmol, 1 eq) in dichloromethane (5.0 mL) was added *L*-Proline (0.09 mmol, 0.3 eq) and diethyl 1,4-dihydro-2,6-dimethyl-3,5-pyridinedicarboxylate (0.33 mmol, 1.1 eq) at room temperature. Then, butyraldehyde (0.90 mmol, 3.0 eq) was added to the reaction system, and stirring was continued for 5 h. After TLC indicated consumption of the starting material, the mixture was filtered, and the filter cake was washed with DCM. The combined organic layers were washed with water and saturated brine, dried over anhydrous Na_2_SO_4_, filtered, and concentrated under reduced pressure. The residue was purified by silica gel column chromatography with petroleum ether/EtOAc (32:1) to afford the product. Yellow solid, 47% yield, mp 111 ± 1 °C. ^1^H NMR (400 MHz, Chloroform-*d*) δ 12.50 (s, 1H), 7.63 (dd, *J* = 7.4, 1.2 Hz, 1H), 7.53 (dd, *J* = 8.4, 7.4 Hz, 1H), 7.28 (dd, *J* = 8.5, 1.2 Hz, 1H), 2.65–2.51 (m, 2H), 1.58–1.47 (m, 2H), 1.47–1.35 (m, 2H), 0.98–0.90 (m, 3H). ^13^C NMR (101 MHz, Chloroform-*d*) δ 190.95, 180.72, 161.39, 153.62, 134.95, 129.45, 126.25, 124.41, 119.21, 114.50, 30.39, 22.87, 22.52, 13.91. HRMS (ESI): calcd for C_14_H_14_O_4_H (M + H^+^) 247.09644; found 247.09645.

**3-decyl-2,5-dihydroxynaphthalene-1,4-dione (25)**. Compound **25** was synthesized from decanal using the same procedure as described for **24**. Yellow solid, 32% yield, mp 106 ± 1 °C. ^1^H NMR (400 MHz, Chloroform-*d*) δ 12.50 (s, 1H), 7.63 (d, *J* = 7.2 Hz, 1H), 7.57–7.49 (m, 1H), 7.33–7.22 (m, 1H), 2.62–2.48 (m, 2H), 1.56–1.47 (m, 2H), 1.29–1.20 (m, 14H), 0.91–0.84 (m, 3H). ^13^C NMR (101 MHz, Chloroform-*d*) δ 191.07, 180.86, 161.51, 153.77, 135.06, 129.59, 126.36, 124.58, 119.32, 114.63, 32.04, 29.91, 29.75, 29.71, 29.57, 29.47, 28.41, 22.92, 22.83, 14.26. HRMS (ESI): calcd for C_20_H_26_O_4_H (M + H^+^) 331.19032; found 331.19016.

**3-hexadecyl-2,5-dihydroxynaphthalene-1,4-dione (26)**. Compound **26** was synthesized from palmitaldehyde using the same procedure as described for **24**. Yellow solid, 22% yield, mp 104 ± 1 °C. ^1^H NMR (500 MHz, Chloroform-*d*) δ 12.49 (s, 1H), 7.64 (dd, *J* = 7.4, 1.1 Hz, 1H), 7.56–7.51 (m, 1H), 7.28 (dd, *J* = 8.5, 1.1 Hz, 1H), 2.62–2.53 (m, 2H), 1.58–1.48 (m, 2H), 1.30–1.20 (m, 26H), 0.91–0.85 (m, 3H). ^13^C NMR (126 MHz, Chloroform-*d*) δ 191.09, 180.88, 161.56, 153.73, 135.08, 129.61, 126.39, 124.60, 119.34, 32.08, 29.92, 29.88, 29.72, 29.59, 29.51, 28.42, 22.94, 22.84, 14.27. HRMS (ESI): calcd for C_26_H_37_O_4_ (M-H^+^) 413.27044; found 413.26970.

### 2.2. Cell Culture and Viability Assay

HaCaT cells (American Type Culture Collection, Manassas, VA, USA) were cultured in DMEM (Gibco, Thermo Fisher Scientific, Grand Island, NY, USA) supplemented with 10% fetal bovine serum (FBS, ZETA LIFE INC., Menlo Park, CA, USA) and 1% penicillin-streptomycin (Gibco, Thermo Fisher Scientific, Grand Island, NY, USA) at 37 °C in a humidified incubator with 5% CO_2_. Juglone and its derivatives were dissolved in DMSO (Sinopharm Chemical Reagent, Shanghai, China) and added to cells seeded in 96-well plates at a density of 1 × 10^4^ cells per well. After 24 h treatment, cell viability was evaluated using the CCK-8 (Suzhou XinSaimei Biotechnology, Suzhou, China) assay, and the absorbance at 450 nm was measured using a microplate reader. Relative cell viability was calculated as follows: cell viability (%) = (ODtreated − ODblank)/(ODcontrol − ODblank) × 100%.

The CC_50_ value was defined as the concentration of compound that reduced cell viability by 50% compared with the untreated control. CC_50_ values were calculated using GraphPad Prism 9.5 by nonlinear regression analysis with a four-parameter logistic concentration-response model. When the fitted CC_50_ value was outside the tested concentration range, it was reported as greater than the highest tested concentration or lower than the lowest tested concentration.

Each concentration was tested in three parallel wells.

### 2.3. Preliminary Anti-Inflammatory Screening Based on NO Production in LPS-Stimulated HaCaT Cells

HaCaT cells were seeded in 24-well plates at a density of 5 × 10^4^ cells per well and incubated for 24 h. Juglone derivatives were then added to each well at a final concentration of 5 μg/mL. After 2 h of pretreatment, LPS was added at a final concentration of 20 μg/mL, and the cells were further incubated for 24 h. The culture supernatants were collected for NO determination. The NO-screening assay was performed at a fixed mass concentration of 5 μg/mL to allow practical comparison of the synthesized derivatives under the same mass-exposure condition.

NO production was measured using a nitrate reductase-based Griess colorimetric assay kit (Beyotime, Shanghai, China) according to the manufacturer’s instructions. Briefly, nitrate in the culture supernatants was reduced to nitrite by nitrate reductase, followed by reaction with Griess chromogenic reagents. After color development, the reaction mixture was centrifuged at 4000 rpm for 10 min, and absorbance of the supernatant was measured at 550 nm using a microplate reader. NO concentrations were calculated using the following formula: NO concentration (μM) = [(Asample − Ablank)/(Astandard − Ablank)] × Cstandard × N, where Cstandard was 100 μM and N represents the sample dilution factor. Raw NO concentrations are provided in Table S2. For graphical presentation, NO production was normalized to the LPS group and expressed as relative NO level (% of LPS group) using the following formula: relative NO level (%) = (NO concentration of treated group/NO concentration of LPS group) × 100%.

For the NO-screening assay, each treatment was tested in three parallel wells as technical replicates.

### 2.4. Cytokine Determination in LPS-Stimulated HaCaT Cells

HaCaT cells were seeded in 24-well plates at a density of 5 × 10^4^ cells per well and incubated for 24 h. Compound **11** was then added at final concentrations of 10, 5, and 1 μg/mL. After 2 h pretreatment, LPS (20 μg/mL) was added, and the cells were incubated for a further 24 h. The culture supernatants were collected, centrifuged to remove cell debris, and the protein levels of TNF-α, IL-6, IL-1β, IL-17A, and IL-23 were determined using commercial ELISA kits (Shanghai Jianglai Biotechnology, Shanghai, China) according to the manufacturer’s instructions.

### 2.5. Animal Treatment

Male BALB/c mice (7–8 weeks old) were randomly divided into six groups (*n* = 6 per group) before treatment: blank control (Vaseline), positive control (clobetasol propionate cream, 0.05%, Fuyuan Pharmaceutical, Xuancheng, Anhui, China), compound **11** at 100 mg/kg, compound **11** at 50 mg/kg, compound **11** at 25 mg/kg, and IMQ model group. All mice were housed under standard conditions (25 ± 1 °C) with free access to food and water.

The severity of psoriasis-like lesions was evaluated daily using PASI-related scores for erythema, scaling, and thickness. Each parameter was scored on a scale from 0 to 4, and the total score was calculated as the sum of the three parameters. PASI-related scoring was performed by observers blinded to the treatment groups to minimize assessment bias.

Compound **11** was prepared in 60% absolute ethanol and 40% glycerol at concentrations of 5, 10, and 20 mg/mL, corresponding to 0.5%, 1.0%, and 2.0% (*w*/*v*), respectively. For topical administration, 100 μL of the formulation was applied evenly to the shaved dorsal skin area of each mouse once daily for 7 days. The treated skin area was approximately 2 cm × 3 cm. Based on a nominal mouse body weight of 20 g, these concentrations corresponded to nominal doses of 25, 50, and 100 mg/kg/day, respectively, or approximately 0.083, 0.167, and 0.333 mg/cm^2^/day.

At the end of the experiment, blood samples were collected from the orbital venous plexus, and the mice were then sacrificed. Part of the dorsal skin tissue was fixed in 4% paraformaldehyde for 48 h, while the remaining skin tissue was stored at −80 °C for subsequent analysis. All animal procedures were approved by the Ethics Committee of the Lanzhou Institute of Husbandry and Pharmaceutical Sciences, Chinese Academy of Agricultural Sciences, and were conducted in accordance with the relevant guidelines for the care and use of laboratory animals (Approval No. 2026-10). The SPF BALB/c mice used in this study were purchased from the Lanzhou Veterinary Research Institute, Chinese Academy of Agricultural Sciences (Lanzhou, China).

### 2.6. Histological Analysis

Dorsal skin tissues were fixed in 4% paraformaldehyde, dehydrated using an automatic tissue processor (JT-12S, Wuhan Junjie Electronics Co., Ltd., Wuhan, China), embedded in paraffin using a tissue embedding machine (BMJ-A, Changzhou Zhongwei Electronic Instrument Co., Ltd., Changzhou, China), and sectioned into 4-μm-thick slices using a rotary microtome (Leica-2016, Leica, Berlin, Germany). The sections were then deparaffinized, rehydrated through a graded ethanol series, stained with hematoxylin and eosin (H&E), dehydrated, cleared, and mounted with neutral balsam. Histopathological images were acquired using a slide scanning imaging system (SQS-600P, Shenzhen Shengqiang Technology Co., Ltd., Shenzhen, China) at 100× and 200× magnifications.

### 2.7. Determination of TNF-α, IL-6, IL-1β, IL-17A, and IL-23 in Serum and Skin Tissues

The levels of TNF-α, IL-6, IL-1β, IL-17A, and IL-23 in serum and skin tissue homogenates were determined using commercial ELISA kits (Shanghai Jianglai Biotechnology, China) according to the manufacturer’s instructions.

### 2.8. Prediction of Potential Psoriasis-Related Targets and Intersection Analysis

Potential targets of juglone were predicted using the SwissTargetPrediction and SuperPred platforms based on its chemical structure. Psoriasis-related targets were collected from the GeneCards database using a relevance score threshold greater than 6. After removal of duplicate entries, the predicted targets of juglone were intersected with psoriasis-associated targets to identify common candidate targets. The overlapping targets were visualized using a Venn diagram and selected for subsequent molecular docking analysis.

### 2.9. Molecular Docking

Molecular docking simulations were performed using Discovery Studio 2021 (DS 2021). Crystal structures of the candidate target proteins were retrieved from the Protein Data Bank (PDB). Before docking, protein structures were prepared by removing water molecules, adding hydrogen atoms, and optimizing side chains. Juglone and compound **11** were docked into the active sites of the selected candidate targets using the CDOCKER protocol, and the docking scores were used to compare their predicted binding affinities.

### 2.10. Computational ADMET Prediction

To evaluate the drug-likeness and pharmacokinetic properties of compound **11**, in silico ADMET prediction was performed using ADMETlab 3.0. The structure of compound **11** was uploaded in SMILES format, and physicochemical, absorption, distribution, metabolism, excretion, and toxicity-related parameters were calculated using the default settings. The predicted results were collected for further analysis.

### 2.11. Statistical Analysis

All experimental data are expressed as mean ± standard deviation (SD). Statistical analysis was performed using GraphPad Prism 9.5. One-way analysis of variance (ANOVA) was used for multiple-group comparisons, followed by Bonferroni’s post hoc test for pairwise comparisons. A value of *p* < 0.05 was considered statistically significant. NO production data were analyzed by one-way ANOVA followed by Bonferroni’s post hoc test. The LPS group was compared with the untreated control group, and compound-treated groups were compared with the LPS group.

## 3. Results

### 3.1. Synthesis of Juglone-Derived Naphthoquinones

**Figure 1:**
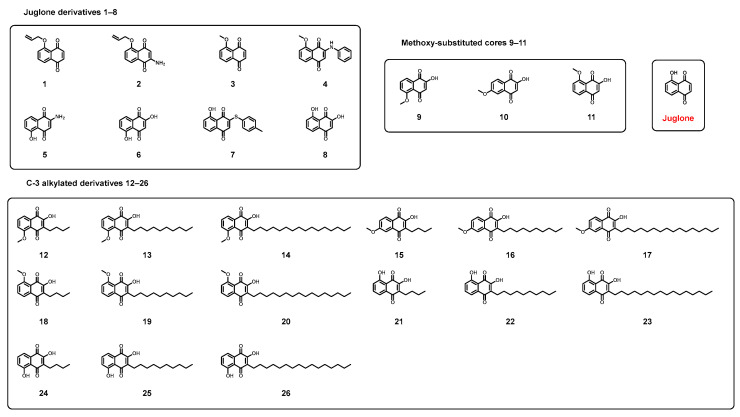
Chemical structures of juglone and synthesized derivatives **1**–**26**.

The chemical structures of juglone and all synthesized derivatives **1**–**26** are summarized in Figure 1.

**Scheme 1:**
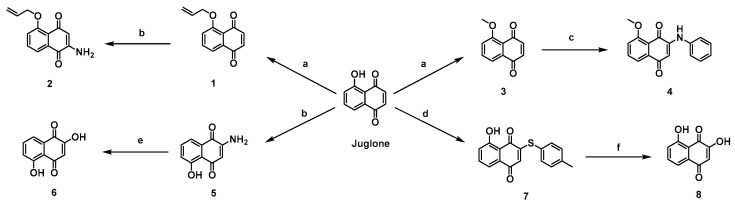
Synthesis of juglone derivatives. Reagents and conditions: (a) Ag_2_O, alkyl halide (MeI or allyl bromide), DCM, rt; (b) *O*-benzylhydroxylamine·HCl, Et_3_N, EtOH, 5 °C to rt; (c) aniline, EtOH, rt, 5 days; (d) *p*-toluenethiol, EtOH, rt; (e) conc. HCl, reflux; (f) NaOH aq, EtOH, reflux.

*O*-Alkylation of juglone was carried out in dichloromethane using methyl iodide or allyl bromide in the presence of Ag_2_O to afford the ether derivatives **1** and **3**. Amination of compound **3** with aniline in ethanol furnished the amino-substituted naphthoquinone **4**. Treatment of compound **1** and juglone with *O*-benzylhydroxylamine hydrochloride afforded the amino derivatives **2** and **5**, which upon hydrolysis gave 2,5-dihydroxy-1,4-naphthoquinone (**6**). In addition, juglone was subjected to thioetherification to provide sulfide derivative **7**, which was further converted into 2,8-dihydroxy-1,4-naphthoquinone (**8**) under basic conditions (Scheme 1).

**Scheme 2:**
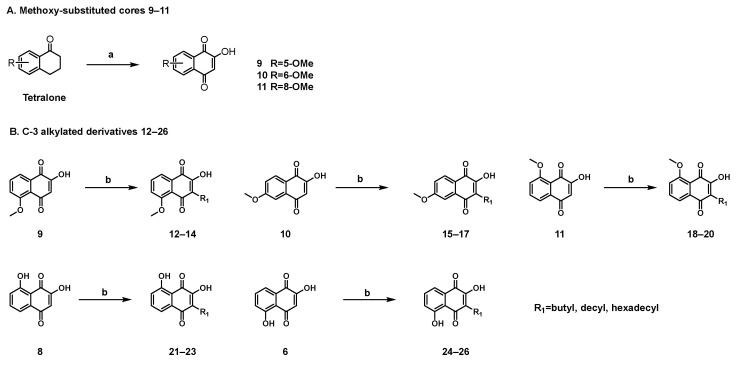
Synthesis of substituted 2-hydroxynaphthoquinone derivatives. Reagents and conditions: (a) *t*-BuOK, *t*-BuOH, O_2_ atmosphere, rt; (b) aldehyde, *L*-proline, Hantzsch ester, DCM, rt.

To investigate the influence of the C-2 hydroxyl group and the substitution pattern on the quinone scaffold, a series of methoxy-substituted 2-hydroxy-1,4-naphthoquinone derivatives based on the juglone framework was synthesized (Scheme 2). Specifically, under an oxygen atmosphere, tetralone derivatives were treated with potassium *tert*-butoxide in *tert*-butanol to afford 2-hydroxy-1,4-naphthoquinones bearing methoxy substituents at the 5-, 6-, and 8-positions (compounds **9**–**11**).

Previous studies have shown that structural modification of 2-hydroxy-1,4-naphthoquinone scaffolds at the C-3 position can markedly alter their biological properties [19]. In particular, C-3-substituted hydroxynaphthoquinones have been reported to display insecticidal and herbicidal activities related to mitochondrial or photosynthetic electron-transport processes, with activity profiles strongly influenced by alkyl-chain length and hydrophobicity [20,21]. In addition, hydroxynaphthoquinone derivatives bearing hydrophobic C-3 side chains have long been recognized as an antimalarial chemotype, while hydroxynaphthoquinone scaffolds more broadly have also shown notable antibacterial potential [19,22]. These observations suggest that C-3 alkyl substitution represents a chemically and biologically sensitive modification site on the 2-hydroxynaphthoquinone scaffold. Therefore, to examine the effect of this structural feature in the present series, a subset of 2-hydroxy-naphthoquinone cores (**6**, **8**, and **9**–**11**) was further modified by introducing alkyl chains of varying lengths (butyl, decyl, and hexadecyl) at the C-3 position, affording compounds **12**–**26**. This design enabled a comparative evaluation of the influence of C-3 alkyl substitution on anti-inflammatory activity and cytotoxicity while retaining the C-2 hydroxyl group as a conserved feature.

The experimental NMR data for each synthesized compound are reported in Section 2.1, and the corresponding NMR spectra are provided in the Supplementary Materials. Overall, these NMR data were consistent with the proposed naphthoquinone structures. In the ^13^C NMR spectra, the characteristic quinone carbonyl carbons were generally observed in the downfield region of approximately 180–191 ppm, supporting the presence of the 1,4-naphthoquinone core. Aromatic carbon signals appeared mainly in the range of approximately 110–165 ppm. For methoxy-substituted derivatives, the OCH_3_ carbon signals were observed at approximately 56 ppm, while the corresponding methoxy proton signals appeared as singlets near 3.9–4.1 ppm in the ^1^H NMR spectra. For alkyl-substituted derivatives, aliphatic proton and carbon signals were observed in the expected upfield regions, including terminal methyl signals near 0.8–0.9 ppm and methylene carbon signals in the range of approximately 22–32 ppm. These characteristic signals, together with the HRMS data reported for each compound in Section 2.1, supported the structural assignments of the synthesized derivatives.

### 3.2. In Vitro Studies with HaCaT Cells

#### 3.2.1. Cytotoxicity Assessment of Juglone Derivative

**Figure 2:**
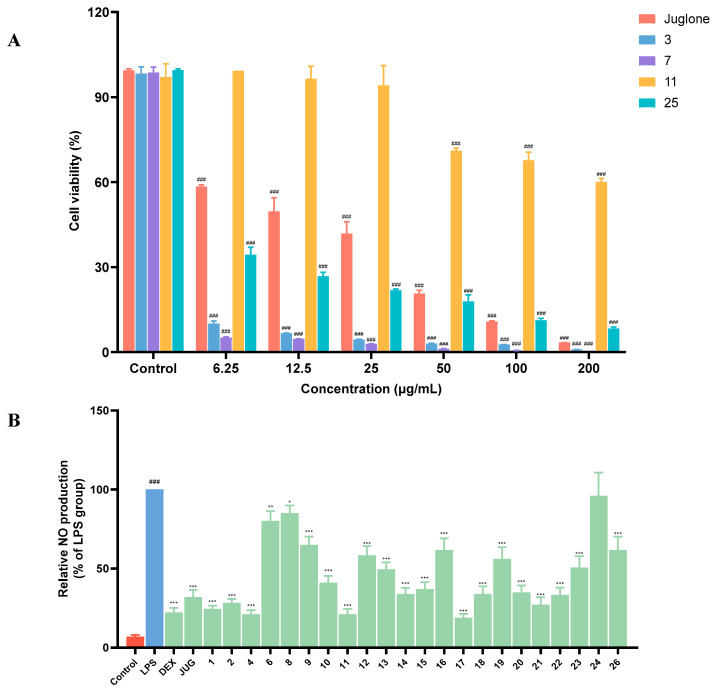
Cytotoxicity and preliminary anti-inflammatory screening of juglone and its derivatives in HaCaT cells. (**A**) Cytotoxicity profiles of juglone (JUG) and representative derivatives (**3**, **7**, **11**, and **25**) were evaluated by the CCK-8 assay after 24 h treatment at the indicated concentrations. Cell viability is expressed as mean ± SD (*n* = 3). (**B**) Effects of dexamethasone (DEX), juglone, and compounds **1**–**26** (5 μg/mL) on LPS-induced nitric oxide (NO) production in HaCaT cells. NO levels were determined by the Griess assay and are expressed as relative percentages compared with the LPS group. Data are presented as mean ± SD from three technical replicate wells (*n* = 3). ^###^ *p* < 0.001 vs. control group; * *p* < 0.05, ** *p* < 0.01, *** *p* < 0.001 vs. LPS group. NO production data were analyzed by one-way ANOVA followed by Bonferroni’s post hoc test.

HaCaT cells, an immortalized human keratinocyte cell line, are widely used as an in vitro model for preliminary evaluation of psoriasis-related inflammatory responses [23,24]. Prior to anti-inflammatory screening, we first assessed the cytotoxicity of juglone and its derivatives in HaCaT cells to define suitable concentration ranges for subsequent biological evaluation. Representative cytotoxicity profiles of juglone and selected derivatives are shown in Figure 2A.

Compared with the parent compound juglone (CC_50_ = 11.66 μg/mL), the majority of derivatives showed markedly reduced cytotoxicity toward HaCaT cells, and many compounds displayed CC_50_ values greater than 200 μg/mL. Notably, compounds **3**, **5**, **7**, and **25** exhibited relatively strong cytotoxicity, with CC_50_ values below 6.25 μg/mL, whereas several derivatives, including **2**, **8**, **9**, **11**, **13**, **16**, **22**, **23**, and **26**, showed negligible cytotoxicity within the tested concentration range. On the basis of these results, compounds **3**, **5**, **7**, and **25** were excluded from subsequent anti-inflammatory screening, while the remaining derivatives were evaluated at a non-cytotoxic concentration of 5 μg/mL.

#### 3.2.2. Preliminary Anti-Inflammatory Screening of Juglone Derivatives

Nitric oxide (NO) is an important inflammatory mediator, and excessive NO production has been associated with inflammatory responses in the skin and other tissues [25]. In psoriasis, dysregulated NO/NOS2 signaling has also been implicated in disease pathogenesis [26]. Accordingly, reduction in NO production can serve as a preliminary readout for evaluating the anti-inflammatory potential of candidate compounds. In the present study, an inflammatory model was established by stimulating HaCaT cells with lipopolysaccharide (LPS, 20 μg/mL), and the effects of juglone derivatives on NO production were evaluated at 5 μg/mL (Figure 2B). Similar LPS-stimulated HaCaT systems have been used in recent anti-inflammatory studies to assess NO- and cytokine-related responses in keratinocytes [27].

As summarized in Table 1, 22 juglone derivatives were subjected to NO screening, excluding compounds **3**, **5**, **7**, and **25** because of their pronounced cytotoxicity. Most of the tested derivatives reduced LPS-induced NO production in HaCaT cells, suggesting that structural modification of the juglone scaffold can substantially influence the biological profile of this series. Compared with juglone (JUG, 17.3 μM), compounds **1**, **2**, **4**, **11**, **17**, and **21** showed lower NO levels under the tested conditions. Among them, compounds **4** (11.4 μM), **11** (11.4 μM), and **17** (10.2 μM) produced some of the lowest NO levels in this series at 5 μg/mL.

**Figure 3:**
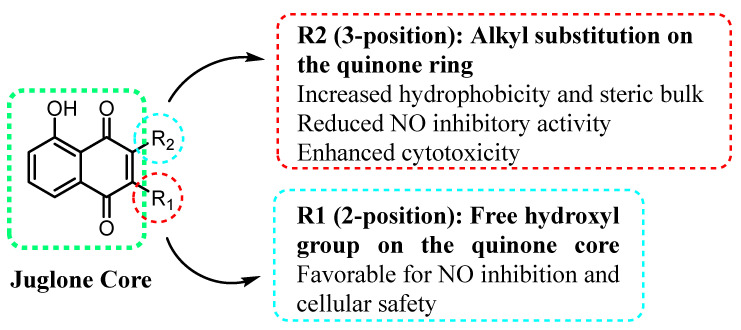
Schematic representation of the SAR of new juglone derivatives.

On the basis of the NO release and cytotoxicity data summarized in Table 1, a preliminary structure–activity relationship (SAR) could be established for these juglone derivatives (Figure 3). Overall, derivatives retaining a free hydroxyl group at the 2-position tended to show more favorable anti-inflammatory activity and lower cytotoxicity than their 3-alkyl-substituted counterparts. Compounds **6**, **8**, and **9**–**11**, which lack bulky alkyl chains at the 3-position, generally showed lower NO release while maintaining acceptable cellular safety. In contrast, C-3 alkyl-substituted derivatives **12**–**26** did not show a consistent improvement in NO suppression across the series. Although compound 17 exhibited strong NO-lowering activity, most C-3 alkylated analogs showed higher NO levels and/or reduced cellular safety compared with the corresponding non-alkylated compounds. Instead, C-3 alkyl substitution generally weakened the reduction in NO release and, in some cases, increased cytotoxicity, particularly for derivatives bearing longer alkyl chains.

For example, compound **11**, which lacks alkyl substitution at the C-3 position, reduced NO release to 11.4 μM and showed negligible cytotoxicity toward HaCaT cells (CC_50_ > 200 μg/mL), whereas its 3-alkyl-substituted analogs **18**–**20** showed higher NO levels (18.3–30.3 μM) together with lower cellular safety (CC_50_ = 32.42–74.99 μg/mL). This comparison further suggests that introduction of alkyl chains at the C-3 position generally compromises both activity and cellular safety. The increased cytotoxicity observed for several C-3 alkyl-substituted derivatives may be associated with changes in lipophilicity, membrane affinity, and the intrinsic redox/electrophilic properties of the naphthoquinone core. Introduction of alkyl chains, particularly longer hydrophobic chains, can increase membrane partitioning and intracellular accumulation, which may enhance nonspecific interactions with cellular membranes and proteins. In addition, modification at the C-3 position may alter the electron distribution of the quinone system and influence redox cycling or Michael-acceptor-like electrophilic reactivity. Such changes could shift the balance from anti-inflammatory modulation toward nonspecific cytotoxicity. Therefore, although C-3 alkyl substitution can tune the physicochemical properties of the scaffold, excessive hydrophobic substitution appears unfavorable for cellular safety in this series.

Taken together, these observations indicate that compound **11** showed the most favorable balance between reduction in NO release and cytotoxicity within this series and was therefore selected for further biological evaluation.

#### 3.2.3. Effect of Compound **11** on Pro-Inflammatory Cytokines TNF-α, IL-6, IL-1β, IL-17A, and IL-23

**Figure 4:**
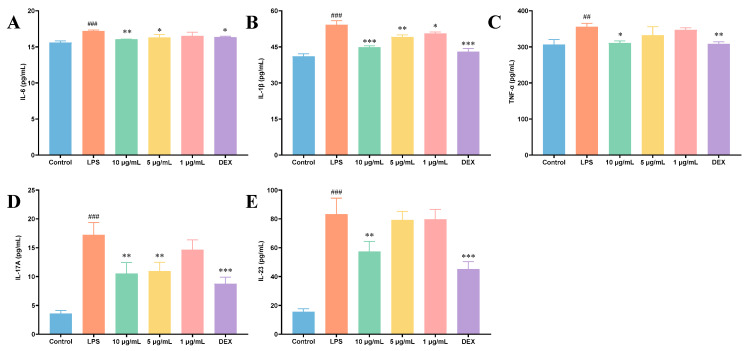
Effects of compound **11** on inflammatory cytokine production in LPS-stimulated HaCaT cells. HaCaT cells were pretreated with compound **11** (10, 5, and 1 μg/mL) for 2 h, followed by stimulation with LPS for 24 h. The levels of IL-6 (**A**), IL-1β (**B**), TNF-α (**C**), IL-17A (**D**), and IL-23 (**E**) in the culture supernatants were quantified by ELISA. Dexamethasone (DEX) was used as a positive control. Data are presented as mean ± SD (*n* = 3). ^##^ *p* < 0.01, ^###^ *p* < 0.001 vs. control group; * *p* < 0.05, ** *p* < 0.01, *** *p* < 0.001 vs. LPS group.

Psoriasis is characterized by marked dysregulation of pro-inflammatory cytokines. Among them, TNF-α, IL-6, and IL-1β contribute to inflammatory amplification and keratinocyte dysfunction, whereas IL-17A and IL-23 form a central pathogenic axis in psoriasis and are closely associated with disease progression [2,28,29,30,31]. Therefore, following the initial screening, compound **11** was further evaluated in LPS (20 μg/mL)-stimulated HaCaT cells by measuring the levels of TNF-α, IL-6, IL-1β, IL-17A, and IL-23 (Figure 4).

Based on the cytotoxicity and preliminary screening results, compound **11** was tested at 1, 5, and 10 μg/mL. In LPS-treated cells, compound **11** at 10 μg/mL significantly suppressed the production of all measured inflammatory cytokines. Treatment at 5 μg/mL markedly reduced the levels of IL-6, IL-1β, and IL-17A, whereas the low dose (1 μg/mL) selectively inhibited IL-1β. Notably, at 10 μg/mL, compound **11** reduced IL-6 and IL-1β levels to 16.06 pg/mL and 44.89 pg/mL, respectively, which were comparable to those achieved with dexamethasone (16.35 pg/mL for IL-6 and 42.96 pg/mL for IL-1β), with cytokine levels approaching those of the control group.

Overall, these results indicate that compound **11** reduced the levels of several inflammatory mediators in the culture supernatants of LPS-stimulated HaCaT cells under the tested conditions. In view of its favorable activity-cytotoxicity balance in the in vitro assays, compound **11** was selected for further evaluation in the IMQ-induced psoriasis-like mouse model.

### 3.3. In Vivo Evaluation in an IMQ-Induced Psoriasis-like Mouse Model

#### 3.3.1. Effect of Compound **11** on Psoriasis-like Skin Lesions and PASI Scores

Based on its favorable activity-cytotoxicity balance in the HaCaT assays, compound **11** was selected for further in vivo evaluation in an imiquimod (IMQ)-induced psoriasis-like mouse model. The IMQ model is widely used for preclinical evaluation of psoriasis-related interventions because it reproduces key features of psoriasiform skin inflammation, including erythema, scaling, epidermal thickening, histopathological alterations, and activation of the IL-23/IL-17 inflammatory axis [29,30,31,32]. Mice were topically treated with compound **11** at nominal doses of 25, 50, and 100 mg/kg/day. Meanwhile, clobetasol propionate (CP) was used as the positive control.

**Figure 5:**
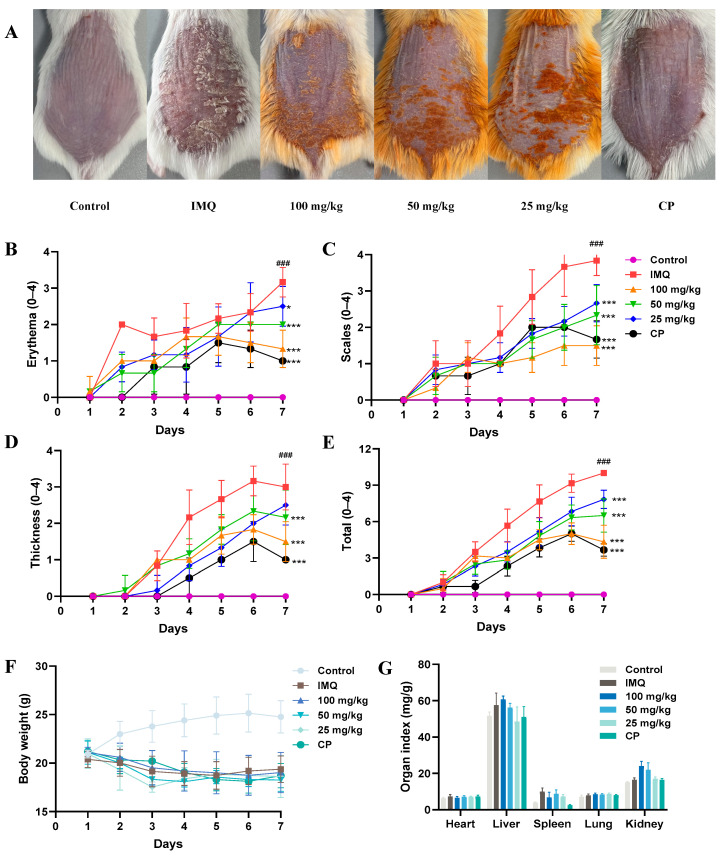
Anti-psoriatic activity and preliminary tolerability assessment of compound **11** in an IMQ-induced psoriasis-like mouse model. (**A**) Representative dorsal skin images of mice from different treatment groups, including the control group, IMQ model group, compound **11**-treated groups (25, 50, and 100 mg/kg/day), and the positive control clobetasol propionate (CP, 2 mg/kg/day). The yellow-brown coloration mainly reflects retention of the colored compound formulation on IMQ-induced scales and hyperkeratotic lesions. (**B**–**E**) Quantitative assessment of psoriasis severity based on PASI-related parameters, including erythema (**B**), scaling (**C**), skin thickness (**D**), and total PASI score (**E**), during the treatment period. (**F**) Changes in body weight of mice throughout the experiment. (**G**) Organ indices of major organs (heart, liver, spleen, lung, and kidney) measured at the end of the experiment. Data are presented as mean ± SD (*n* = 6). The indicated doses are nominal mg/kg/day doses. Compound **11** was applied at 100 μL/mouse/day over an approximately 6 cm^2^ dorsal skin area, corresponding to approximately 0.083, 0.167, and 0.333 mg/cm^2^/day for the low-, medium-, and high-dose groups, respectively. Final-day PASI-related scores were statistically analyzed by one-way ANOVA followed by Bonferroni’s post hoc test. For clarity, statistical annotations for PASI-related scores are shown at the final scoring time point. No significant differences in body weight or major organ indices were observed between the compound **11**-treated groups and the IMQ group under the tested conditions. ^###^ *p* < 0.001 vs. control group; * *p* < 0.05, *** *p* < 0.001 vs. IMQ group.

Compared with the control group, mice in the IMQ-treated group progressively developed typical psoriasis-like skin lesions, including marked erythema, scaling, and thickening of the dorsal skin. By contrast, topical treatment with compound **11** alleviated these IMQ-induced changes to different extents, as reflected by the representative dorsal skin images shown in Figure 5A. Visually, the treated groups exhibited reduced erythema and scaling compared with the IMQ group, with the improvement becoming more apparent at the medium and high doses.

To further assess disease severity, psoriasis area and severity index (PASI)-related parameters, including erythema, scaling, thickness, and total PASI score, were recorded throughout the treatment period (Figure 5B–E). IMQ application resulted in a progressive increase in all PASI-related scores, whereas treatment with compound **11** attenuated these changes under the tested conditions. The medium- and high-dose groups generally showed greater improvement than the low-dose group, while the positive control CP remained more effective overall. These findings suggest that compound **11** partially alleviated IMQ-induced psoriasis-like skin lesions in mice under the tested conditions, with more apparent effects observed in the medium- and high-dose groups.

#### 3.3.2. Histopathological Evaluation of Dorsal Skin Tissues

**Figure 6:**
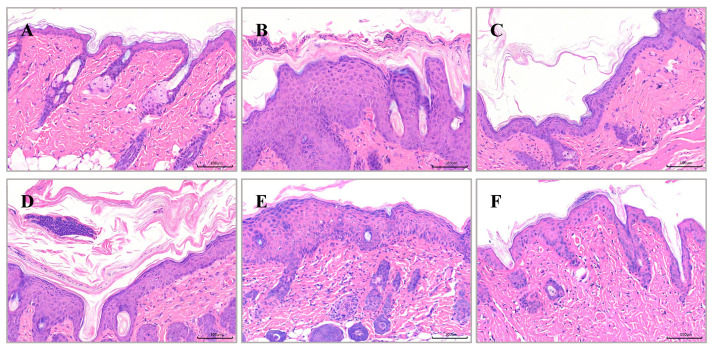
Compound **11** ameliorated IMQ-induced histopathological alterations in dorsal skin tissues of mice (H&E staining, scale bar = 100 μm). (**A**) Control group; (**B**) IMQ group; (**C**) IMQ + compound **11** (100 mg/kg) group; (**D**) IMQ + compound **11** (50 mg/kg) group; (**E**) IMQ + compound **11** (25 mg/kg) group. (**F**) IMQ + clobetasol propionate (CP) group. Dose groups were the same as described in Figure 5. The IMQ group showed typical psoriasis-like histopathological alterations, including hyperkeratosis, parakeratosis, Munro microabscess-like neutrophil accumulation, epidermal thickening, and inflammatory cell infiltration. Compound **11**-treated groups showed partial improvement of these pathological features, especially reduced epidermal thickening and inflammatory infiltration compared with the IMQ group.

Hematoxylin and eosin (H&E) staining was performed to examine histopathological changes in dorsal skin tissues (Figure 6). Compared with the control group, mice in the IMQ-treated group exhibited typical psoriasis-like pathological features, including marked epidermal hyperplasia, pronounced epidermal thickening, and inflammatory cell infiltration in the dermis. In contrast, treatment with compound **11** mitigated these histopathological alterations to varying degrees. In the treated groups, epidermal thickness was reduced and inflammatory infiltration was alleviated compared with the IMQ model group. These findings further suggest that compound **11** may partially improve IMQ-induced histopathological alterations under the tested conditions.

#### 3.3.3. Effect of Compound **11** on Inflammatory Cytokine Levels in Serum and Skin Tissues

**Figure 7:**
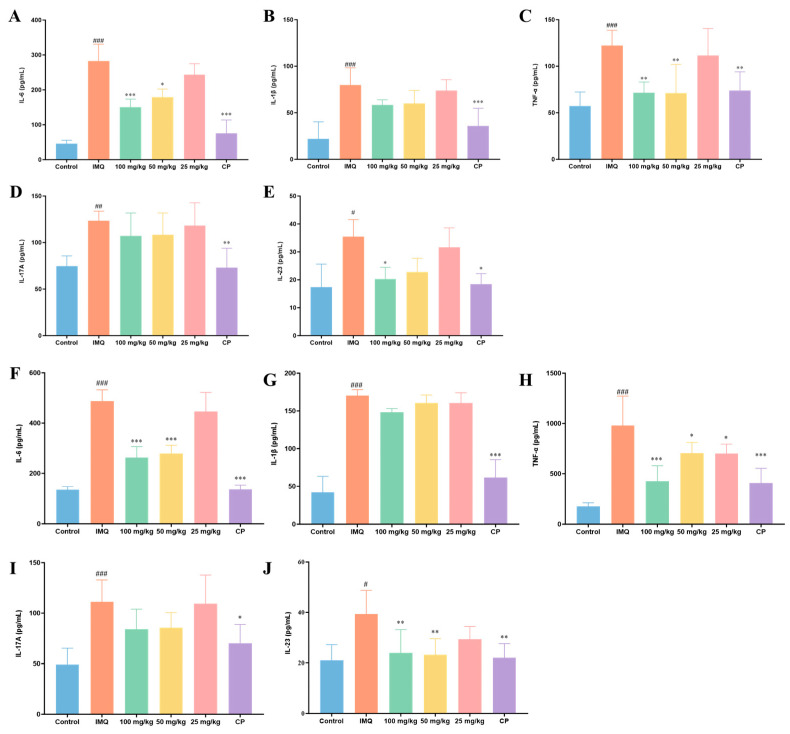
Effects of compound **11** on inflammatory cytokine levels in an IMQ-induced psoriasis-like mouse model. Levels of pro-inflammatory cytokines in serum (**A**–**E**) and skin tissues (**F**–**J**) were quantified by ELISA, including IL-6, IL-1β, TNF-α, IL-17A, and IL-23. Mice were treated with compound **11** at different doses (100, 50, and 25 mg/kg), while clobetasol propionate (CP, 0.05%) was used as a positive control. Data are presented as mean ± SD (*n* = 6), ^#^ *p* < 0.05, ^##^ *p* < 0.01, ^###^ *p* < 0.001 compared with the control group, * *p* < 0.05, ** *p* < 0.01, *** *p* < 0.001 compared with the IMQ group. Dose groups were the same as described in Figure 5.

To further evaluate the anti-inflammatory effects of compound **11** in vivo, the levels of IL-6, IL-1β, TNF-α, IL-17A, and IL-23 in serum (Figure 7A–E) and skin tissues (Figure 7F–J) were determined by ELISA. As expected, IMQ stimulation markedly increased the levels of these pro-inflammatory cytokines in both serum and skin tissues compared with the control group.

Treatment with the positive control CP significantly reduced the levels of these cytokines. Similarly, administration of compound **11** resulted in a marked decrease in inflammatory cytokine production. In particular, compound **11** reduced IL-6, IL-23, and TNF-α levels in both serum and skin tissues. Notably, at 100 mg/kg, compound **11** reduced the IL-23 level in skin tissues to 23.9 pg/mL, which was close to that observed in the control group (20.93 pg/mL). Overall, these results indicate that compound **11** attenuated IMQ-induced inflammatory responses in mice by suppressing the production of key psoriasis-associated cytokines.

#### 3.3.4. Effect of Compound **11** on Body Weight in Psoriasis-like Mice

To obtain a preliminary indication of tolerability during topical treatment, body weight changes were monitored throughout the experimental period. As shown in Figure 5F, no obvious abnormal body-weight loss attributable to compound **11** treatment was observed compared with the IMQ model group. Body weight remained generally stable in the treated groups during the course of the experiment, suggesting that topical administration of compound **11** was well tolerated under the tested conditions.

#### 3.3.5. Effect of Compound **11** on Organ Indices in Psoriasis-like Mice

To further assess the potential systemic impact of compound **11**, organ indices (organ weight-to-body weight ratios) were determined at the end of the experiment. Major organs, including the heart, liver, spleen, lung, and kidney, were collected and weighed. As shown in Figure 5G, no significant differences in organ indices were observed between the compound **11**-treated groups and the IMQ group. These findings provide a preliminary indication that topical treatment with compound **11** did not produce obvious organ-related toxicity under the tested conditions.

### 3.4. Prediction of Potential Psoriasis-Related Targets and Exploratory Molecular Docking Analysis of Compound ***11***

To obtain preliminary insight into the potential target profile of compound **11** in psoriasis-related inflammation, psoriasis-associated targets were first collected from the GeneCards database [33]. Because compound **11** is a juglone derivative, candidate targets were predicted using the parent scaffold juglone with SwissTargetPrediction and SuperPred [34,35]. After data integration and removal of duplicate entries, intersection analysis between juglone-related targets and psoriasis-associated targets yielded six overlapping candidates: STAT3, PTPN22, NFκB1, ACE, MMP2, and MMP9 (Figure 7A).

**Figure 8:**
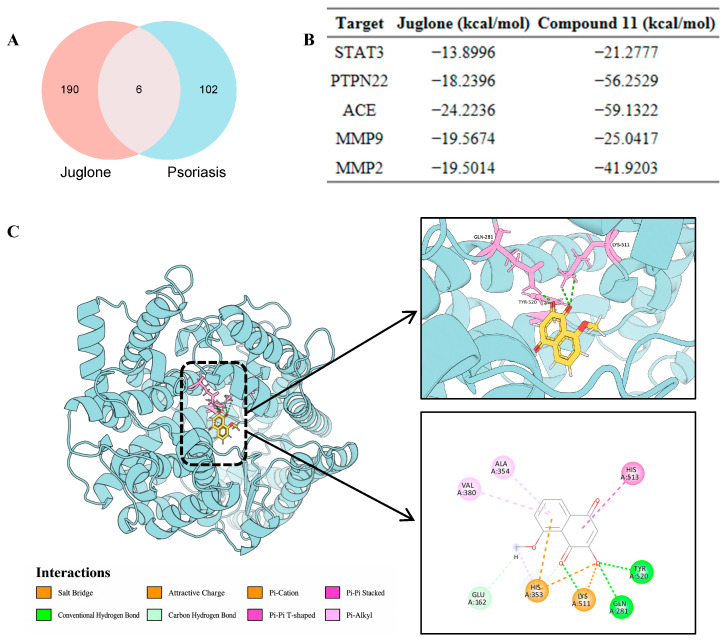
Prediction of psoriasis-related targets and molecular docking analysis of compound **11**. (**A**) Venn diagram showing the overlap between juglone-related targets predicted by SwissTargetPrediction and SuperPred and psoriasis-associated targets collected from the GeneCards database. Six overlapping candidate targets were identified, including STAT3, PTPN22, NFKB1, ACE, MMP2, and MMP9. (**B**) Comparison of docking binding energies of juglone and compound **11** toward five dockable targets (STAT3, PTPN22, ACE, MMP2, and MMP9) obtained using Discovery Studio 2021. NF-κB1 was excluded from the docking analysis due to the absence of a well-defined small-molecule binding pocket. (**C**) Computationally predicted binding mode of compound **11** within the active site of angiotensin-converting enzyme (ACE, PDB ID: 1O86), highlighting possible intermolecular interactions.

To further examine whether compound **11** could interact with these candidates, molecular docking studies were performed for juglone and compound **11** using Discovery Studio 2021. Because NFκB1 functions primarily as a transcription factor and does not present a well-defined small-molecule binding pocket suitable for conventional structure–based docking, docking analysis was limited to the remaining five targets. As shown in Figure 7B, compound **11** displayed more favorable docking scores than juglone toward all five docked proteins, including STAT3 (−21.2777 vs. −13.8996 kcal/mol), PTPN22 (−56.2529 vs. −18.2396 kcal/mol), ACE (−59.1322 vs. −24.2236 kcal/mol), MMP9 (−25.0417 vs. −19.5674 kcal/mol), and MMP2 (−41.9203 vs. −19.5014 kcal/mol). Among these candidates, ACE showed the lowest docking energy for compound **11**, suggesting that it may represent a computationally favored potential target candidate within this set. The predicted binding mode of compound **11** within the active site of ACE (PDB ID: 1O86) is shown in Figure 8C. The docking pose suggests that compound **11** can be accommodated within the ACE binding pocket and may form multiple stabilizing interactions with surrounding residues. Taken together, these results provide preliminary computational evidence for possible target-level interactions of compound **11**, with ACE emerging as a priority candidate for further mechanistic validation.

### 3.5. In Silico ADMET Evaluation of Compound ***11***

The ADMET properties of compound **11** were predicted using ADMETlab 3.0 (Table 2) [36]. The parameters included in Table 2 were selected to provide a concise overview of physicochemical properties, drug-likeness, absorption, distribution, and selected safety-related risks, while additional predicted parameters are provided in Table S3 of the Supplementary Materials. Compound **11** showed a relatively simple and favorable physicochemical profile, including low molecular weight (204.04 Da), moderate polarity (TPSA = 63.6 Å^2^), and balanced lipophilicity (logP = 1.335). The low molecular weight and moderate TPSA suggest that compound **11** possesses a compact structure with an acceptable polar surface area, while the logP value indicates moderate lipophilicity. In addition, compound **11** fully complied with Lipinski’s rule of five, suggesting acceptable drug-likeness from a physicochemical perspective. Predicted absorption and distribution parameters indicated high human intestinal absorption probability (HIA = 0.958) and very low blood–brain barrier penetration probability (BBB = 0.001). These results suggest that compound **11** may have favorable predicted intestinal absorption and a low likelihood of central nervous system exposure. With respect to the selected safety-related predictions in Table 2, compound **11** showed a low predicted probability of hERG inhibition (0.056), suggesting a low predicted risk of hERG-related cardiotoxicity, and a low predicted cytotoxicity probability in the A549 model (0.084). Overall, these computational results suggest that compound **11** possesses a preliminarily acceptable developability profile within the scope of the evaluated parameters. Nevertheless, these ADMET results are based on in silico prediction and should be interpreted as supportive information that requires further experimental validation.

Predicted absorption and distribution parameters indicated high human intestinal absorption probability (HIA = 0.958) and very low blood–brain barrier penetration probability (BBB = 0.001), suggesting a low likelihood of central nervous system exposure.

With respect to the available safety-related predictions in Table 2, compound **11** showed a low predicted probability of hERG inhibition (0.056) and low predicted cytotoxicity in the A549 model (0.084). Overall, these computational results suggest that compound **11** possesses a preliminarily acceptable developability profile within the scope of the evaluated parameters.

## 4. Discussion

The present study was based on the hypothesis that structural modification of juglone could attenuate its intrinsic cytotoxicity while retaining, or potentially improving, its anti-inflammatory and antipsoriatic-relevant activity. The overall results support the feasibility of this strategy. Compared with the parent compound, many of the synthesized juglone-derived naphthoquinones displayed markedly reduced cytotoxicity in HaCaT cells, indicating that the biological profile of this quinone scaffold can be substantially modulated by structural modification. This observation is meaningful in the context of quinone-based drug discovery, because the biological activity and cytotoxicity of quinone-containing compounds can be closely associated with redox cycling and electrophilic reactions with cellular nucleophiles [37]. In the present series, modification of the juglone core appeared to alleviate this limitation to some extent while preserving measurable anti-inflammatory activity, supporting the feasibility of using juglone as a natural-product-inspired starting point for antipsoriatic lead exploration.

The preliminary SAR trends observed in this work further suggest that the substitution pattern around the naphthoquinone nucleus plays an important role in determining the activity-safety balance. In particular, retention of a free hydroxyl group at the C-2 position was generally associated with more favorable anti-inflammatory performance and lower cytotoxicity, whereas alkyl substitution at the C-3 position frequently weakened NO-lowering activity and, in several cases, reduced cellular safety. This trend implies that the C-2 hydroxyl group may help maintain a favorable electronic and physicochemical environment for biological activity, whereas bulky hydrophobic substituents at C-3 may introduce steric and lipophilic penalties that compromise the overall profile. Such an interpretation is consistent with previous studies showing that hydroxynaphthoquinone activity is highly sensitive to substitution pattern, especially at positions that influence hydrogen-bonding capacity and hydrophobicity. In the present study, however, increasing hydrophobic bulk at C-3 did not benefit the psoriasis-related anti-inflammatory profile of this scaffold, suggesting that the structural requirements for this activity may differ from those reported for other hydroxynaphthoquinone-based biological effects.

Within this series, compound **11** showed the most favorable balance between inhibition of inflammatory mediators and cellular safety and therefore emerged as the most promising analog for further study. In addition to reducing NO production in LPS-stimulated HaCaT cells, compound **11** also decreased the protein levels of multiple psoriasis-relevant inflammatory mediators, including TNF-α, IL-6, IL-1β, IL-17A, and IL-23. This broader cytokine modulation is notable because psoriasis is driven by a complex inflammatory network rather than by a single mediator, with the IL-23/IL-17 axis occupying a central pathogenic role and cytokines such as TNF-α, IL-6, and IL-1β contributing to amplification of keratinocyte-associated inflammation. From this perspective, the activity of compound **11** appears to extend beyond simple NO reduction and suggests broader anti-inflammatory potential relevant to psoriasis-like responses. At the same time, because IL-17A and IL-23 are classically associated with immune-cell-driven responses, the changes observed in LPS-stimulated HaCaT cells should be interpreted as cytokine-related inflammatory readouts in a keratinocyte-based screening model, rather than as direct evidence of complete IL-23/IL-17 axis regulation.

The in vivo data in the IMQ-induced psoriasis-like mouse model further strengthened the biological relevance of compound **11**. Topical treatment alleviated erythema, scaling, and epidermal thickening, improved histopathological alterations, and reduced inflammatory cytokine levels in serum and skin tissues under the tested conditions. These findings are important because the IMQ model reproduces several characteristic features of psoriasiform inflammation, including epidermal hyperplasia and activation of IL-23/IL-17-related inflammatory responses. The consistency between the in vitro cytokine results and the in vivo efficacy trends suggests that the activity of compound **11** was not limited to a single assay endpoint, but remained detectable in a psoriasis-like disease context. As expected, the positive control showed stronger overall efficacy, indicating that compound **11** should currently be viewed as a promising lead compound rather than an optimized candidate. The body-weight and organ-index data further suggest preliminary tolerability under the tested conditions, although more systematic safety assessment will be needed in future studies.

The computational analyses included in this study provide preliminary mechanistic and ADMET-related support. The target-prediction and docking workflow identified several psoriasis-related proteins as potential candidates, and compound **11** showed more favorable docking scores than juglone toward the evaluated targets, with ACE giving the most favorable score within this set. This result is of potential interest because it suggests possible target-level interactions that may contribute to the observed anti-inflammatory profile of compound **11**. Because ACE inhibitors are widely used in cardiovascular medicine, this result deserves attention from both mechanistic and safety perspectives. At this stage, the docking result should be interpreted as a computational indication of possible ACE-related interaction rather than a direct enzymatic readout. Therefore, future ACE enzymatic assays and target-engagement experiments would further clarify whether compound **11** has measurable ACE-modulating activity and whether this interaction contributes to its anti-inflammatory profile or potential off-target effects. Considering the electrophilic and redox-active nature of the naphthoquinone scaffold, additional interaction modes may also contribute to the biological activity of these derivatives. Previous studies have shown that natural naphthoquinones can act on enzyme targets such as sortase A, suggesting that both non-covalent recognition and scaffold-dependent chemical reactivity may be relevant for this class of compounds [38]. In the present study, molecular docking was used as an exploratory tool to compare possible non-covalent interactions between compound **11** and selected psoriasis-related targets. These computational findings provide mechanistic hypotheses that merit further validation by target-engagement and biochemical assays.

Future work should therefore focus on further optimization around compound **11**, with particular attention to maintaining the favorable activity-safety balance while improving potency and topical suitability. Mechanistic studies are needed to clarify whether the observed effects are associated with modulation of keratinocyte inflammatory signaling, oxidative stress-related pathways, immune-cell-associated cytokine networks, or any of the computationally suggested targets. It would also be valuable to expand the chemical space around this scaffold through more subtle substituent variation rather than extensive C-3 hydrophobic chain extension, which appeared unfavorable in the current series. In addition, the inherent yellow-to-orange color of naphthoquinone derivatives should be considered for potential topical application, as visible staining may affect cosmetic acceptability and patient compliance. Future formulation studies, such as gel, cream, microemulsion, or encapsulated delivery systems, will be needed to improve topical applicability, reduce visible residue, and evaluate skin retention and irritation. Taken together, the present results identify juglone-derived naphthoquinones as a tractable natural-product-inspired chemotype for antipsoriatic lead exploration and position compound **11** as a useful starting point for continued medicinal chemistry and pharmacological investigation.

## 5. Conclusions

In conclusion, a series of juglone-derived naphthoquinone analogs was synthesized and evaluated to investigate their preliminary anti-inflammatory potential in psoriasis-related experimental models. Structural modification of the juglone scaffold markedly improved the biological profile of many derivatives by reducing cytotoxicity relative to the parent compound. In LPS-stimulated HaCaT cells, most compounds showed reduced NO production together with improved cellular safety, and preliminary SAR analysis indicated that retention of a free hydroxyl group at the C-2 position was generally favorable, whereas alkyl substitution at the C-3 position tended to compromise the activity-safety balance. In the preliminary in vitro screening, compound **11** showed the most favorable balance between NO reduction and cellular safety.

Compound **11** further suppressed multiple pro-inflammatory cytokines in vitro and alleviated psoriasis-like symptoms, histopathological changes, and inflammatory cytokine production in an IMQ-induced psoriasis-like mouse model, while showing preliminary tolerability under the tested conditions. Although the mechanistic basis of its activity remains to be clarified, the exploratory docking and in silico ADMET results provided supportive information for further investigation. Overall, this study indicates that structural modification of the juglone scaffold can generate naphthoquinone derivatives with improved psoriasis-related anti-inflammatory profiles and supports compound **11** as a useful starting point for further optimization and mechanistic validation.

## Figures and Tables

**Table 1:** Structures and biological results of derivatives.

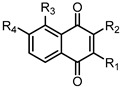						
Compound	R1	R2	R3	R4	NO Generation (μM, Mean ± SD, *n* = 3)	HaCaT (CC_50_, μg/mL)
**1**	H	H	O-allyl	H	13.3 ± 1.3	23.19
**2**	NH2	H	O-allyl	H	15.3 ± 1.4	>200
**3**	H	H	O-Me	H	ND	<6.25
**4**	H	PhNH-	O-Me	H	11.4 ± 1.1	129.9
**5**	NH2	H	OH	H	ND	<6.25
**6**	OH	H	OH	H	43.3 ± 3.7	43.22
**7**	H	4-MePhS-	OH	H	ND	<6.25
**8**	H	OH	OH	H	46.0 ± 3.7	>200
**9**	OH	H	OMe	H	35.1 ± 3.2	>200
**10**	OH	H	H	OMe	22.2 ± 2.5	55.58
**11**	H	OH	OMe	H	11.4 ± 1.4	>200
**12**	OH	butyl	OMe	H	31.5 ± 3.4	25.93
**13**	OH	decyl	OMe	H	26.7 ± 2.8	>200
**14**	OH	hexadecyl	OMe	H	18.3 ± 2.2	30.04
**15**	OH	butyl	H	OMe	20.0 ± 2.4	180.60
**16**	OH	decyl	H	OMe	33.3 ± 4.1	>200
**17**	OH	hexadecyl	H	OMe	10.2 ± 1.4	176.4
**18**	butyl	OH	OMe	H	18.3 ± 2.5	74.99
**19**	decyl	OH	OMe	H	30.3 ± 4.0	32.42
**20**	hexadecyl	OH	OMe	H	18.9 ± 2.4	47.82
**21**	butyl	OH	OH	H	14.7 ± 2.5	176.80
**22**	decyl	OH	OH	H	18.0 ± 2.5	>200
**23**	hexadecyl	OH	OH	H	27.3 ± 3.9	>200
**24**	OH	butyl	OH	H	51.6 ± 4.5	48.31
**25**	OH	decyl	OH	H	ND	<6.25
**26**	OH	hexadecyl	OH	H	33.3 ± 4.7	>200
**J** **uglone**	H	H	OH	H	17.3 ± 2.4	11.68
**D** **EX**	-	-	-	-	12.0 ± 1.6	-
**LPS**	-	-	-	-	54.1 ± 3.6	-

ND, not determined. Compounds **3**, **5**, **7**, and **25** were not subjected to NO screening because they showed pronounced cytotoxicity toward HaCaT cells and were therefore unsuitable for NO-based anti-inflammatory evaluation at the screening concentration. NO production values are presented as mean ± SD from three replicates. Raw replicate values, including the untreated control, LPS, DEX, JUG, and compound-treated groups, are provided in Table S2.

**Table 2:** In silico ADMET properties of compound **11**.

Category	Parameter	Value
Physicochemical	Molecular weight (Da)	204.04
Physicochemical	TPSA (Å^2^)	63.6
Physicochemical	logP (pH 7.4)	1.335
Drug-likeness	Lipinski’s rule	0 violations
Absorption	Human intestinal absorption (HIA, probability)	0.958
Distribution	Blood–brain barrier penetration (BBB, probability)	0.001
Safety	hERG inhibition probability	0.056
Safety	Cytotoxicity (A549, probability)	0.084

## Data Availability

The original contributions presented in this study are included in the article and Supplementary Materials. Further inquiries can be directed to the corresponding author.

## Supplementary Materials

The following supporting information can be downloaded at: https://www.mdpi.com/article/10.3390/biom16060802/s1, Data S1: NMR spectra of all juglone derivatives; Data S2: HPLC purity analysis of representative active compounds **4** and **11**; Figure S1: HPLC chromatogram of compound **4**; Figure S2: HPLC chromatogram of compound **11**; Table S1: Raw cell viability data (%) of juglone and its derivatives in HaCaT cells after 24 h treatment at the indicated concentrations, determined by the CCK-8 assay; Table S2: Raw nitric oxide (NO) production data of juglone and its derivatives in LPS-stimulated HaCaT cells; Table S3: Predicted ADMET properties of compound **11**.

## Abbreviations

The following abbreviations are used in this manuscript:

ACE	Angiotensin-converting enzyme
ADMET	Absorption, Distribution, Metabolism, Excretion, and Toxicity
CCK-8	Cell Counting Kit-8
CP	Clobetasol propionate
DEX	Dexamethasone
ELISA	Enzyme-linked immunosorbent assay
H&E	Hematoxylin and eosin
HaCaT	Human immortalized keratinocyte cell line
IL	Interleukin
IMQ	Imiquimod
LPS	Lipopolysaccharide
NO	Nitric oxide
PASI	Psoriasis Area and Severity Index
SAR	Structure–activity relationship
TNF-α	Tumor necrosis factor alpha
