# Supplementary material for: Synthesis, Biological Evaluation and Structure–Activity Relationship of Juglone Derived Naphthoquinones as Potential Antipsoriatic Agents
Source: Biomolecules. 2026 May 29;16(6):802. doi: 10.3390/biom16060802 (PMC13297244; doi:10.3390/biom16060802)
Supplement: Supplementary file 1 [file biomolecules-16-00802-s001.zip › biomolecules-4286677-supplementary.pdf]

Synthesis, biological evaluation and structure activity relationship of juglone derived naphthoquinones as potential antipsoriatic agents

Tong Bu [1,2], Zile Gong [1,2], Yudong Ma [2], Lixia Dai [2], Yuchao Ma [2], Xiaoyan Yu [1,2], Xiaorong Yang [2], Xiaolou Miao [2], Xiaofei Shang [1,2,*]

[1] College of Pharmacy, Gansu University of Chinese Medicine, Lanzhou 730000, Gansu, PR China

[2] Key Laboratory of New Animal Drug Project, Gansu Province, Key Laboratory of Veterinary Pharmaceutical Development of Ministry of Agriculture and Rural Affairs, Lanzhou Institute of Husbandry and Pharmaceutical Sciences, Chinese Academy of Agricultural Sciences, Lanzhou 730050, PR China

* Correspondence: shangxiaofei@caas.cn (X. Shang)

Data S1: NMR spectra of all juglone derivatives

The 1H NMR spectra were used to support the structural assignment and apparent purity assessment of the isolated compounds.

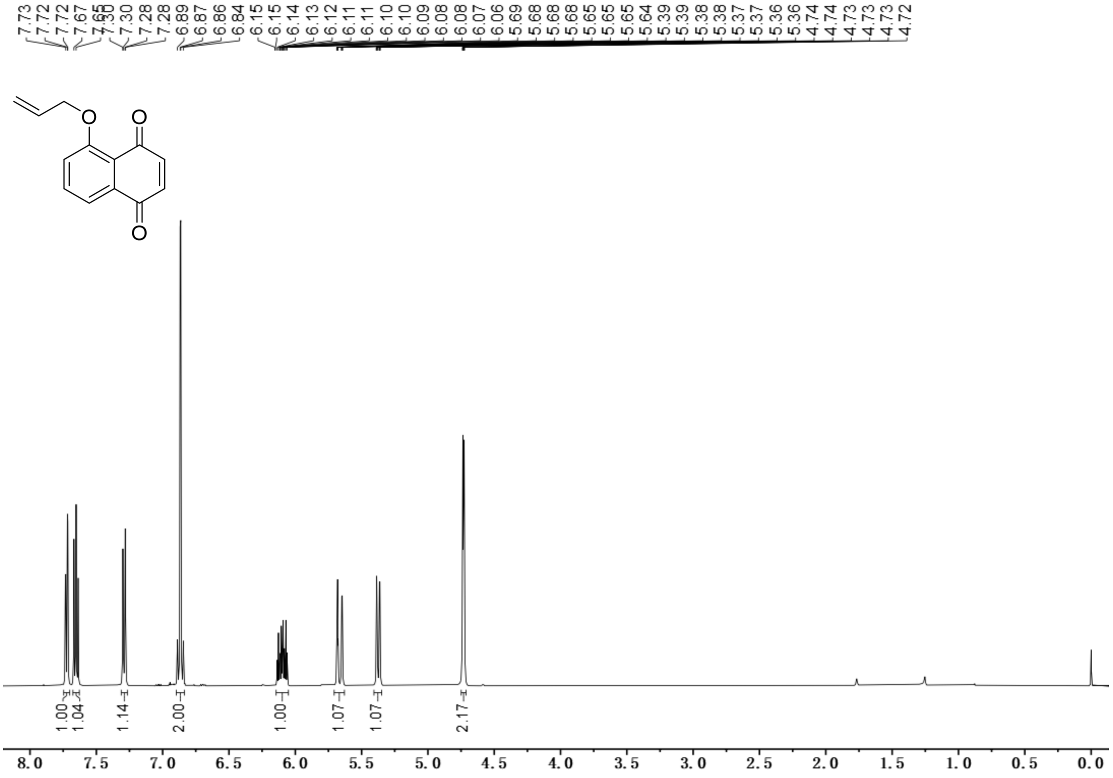

^{1}H NMR Spectra of **1**

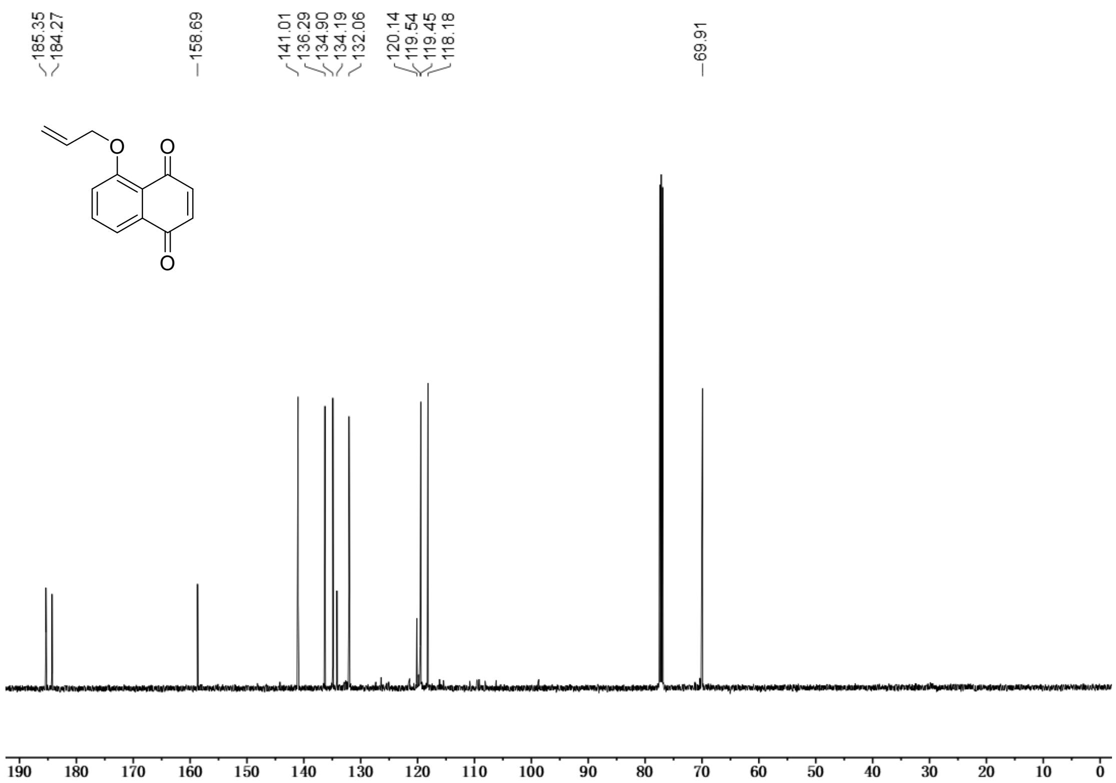

^{13}C NMR Spectra of **1**

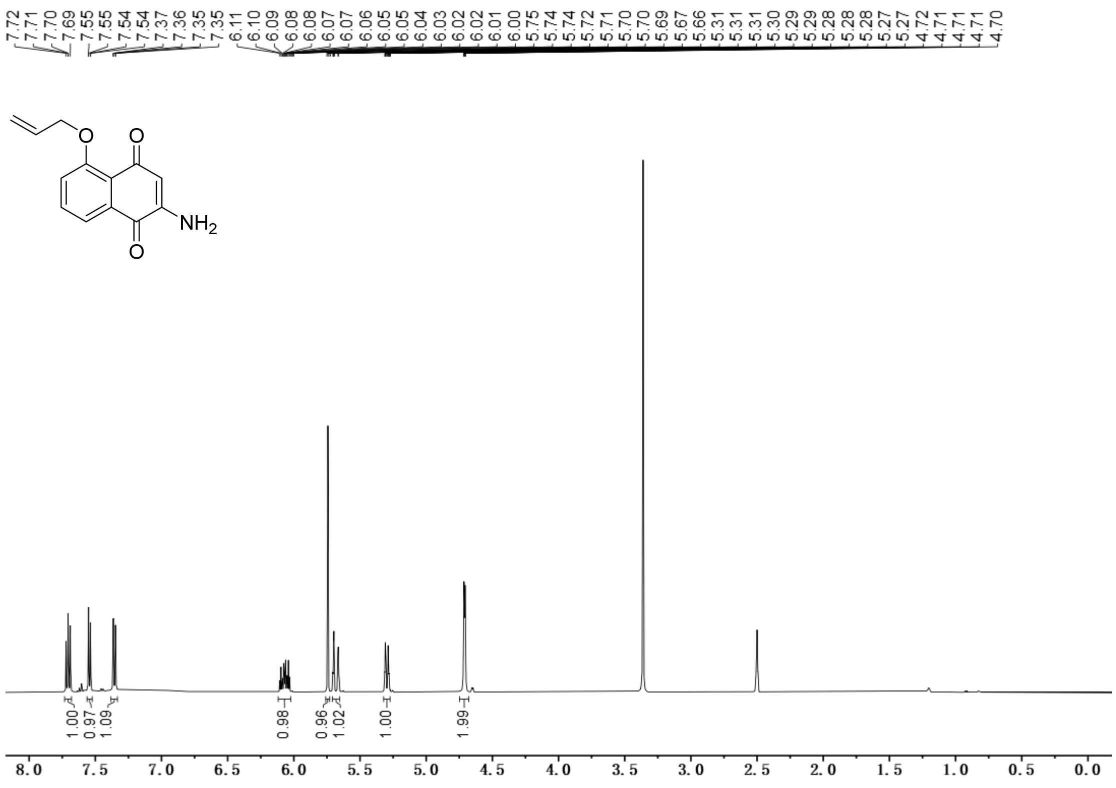

^{1}H NMR Spectra of **2**

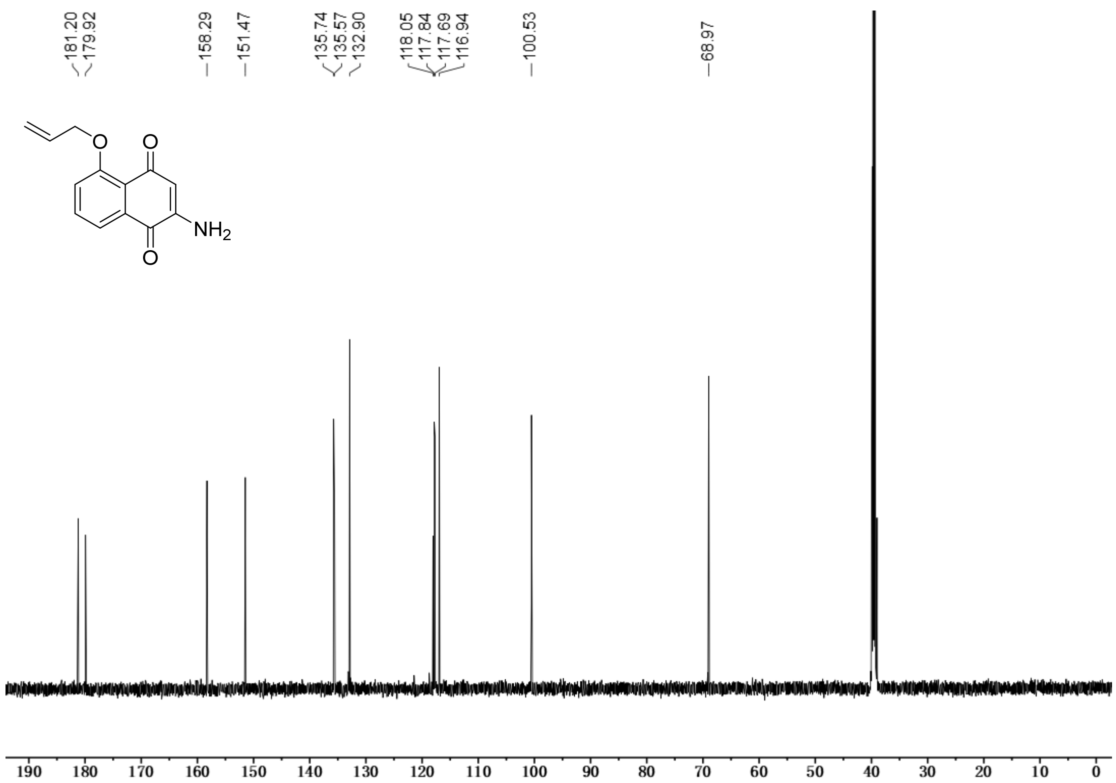

^{13}C NMR Spectra of **2**

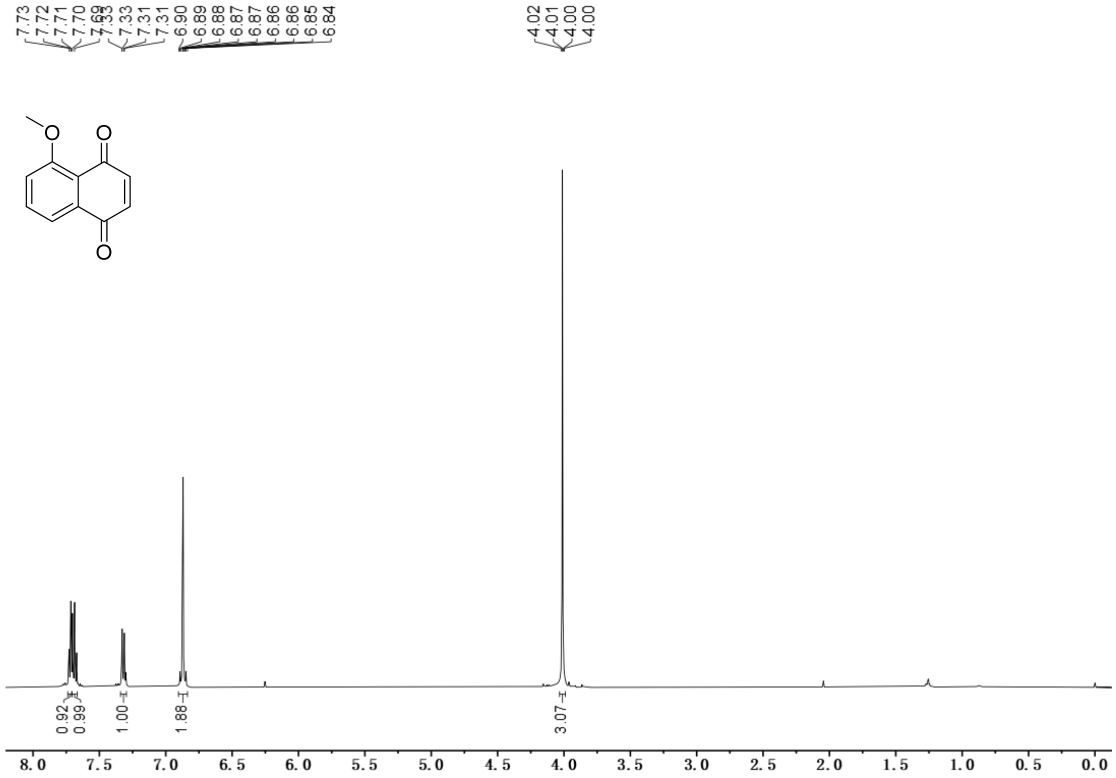

^{1}H NMR Spectra of **3**

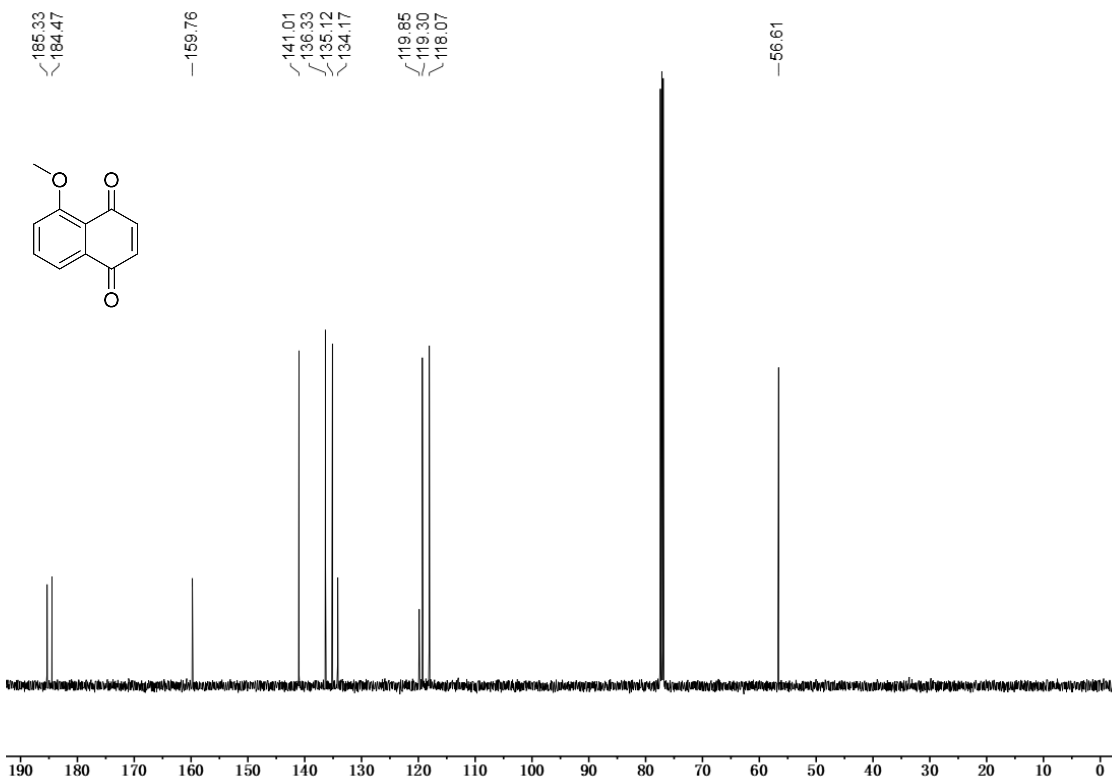

^{13}C NMR Spectra of **3**

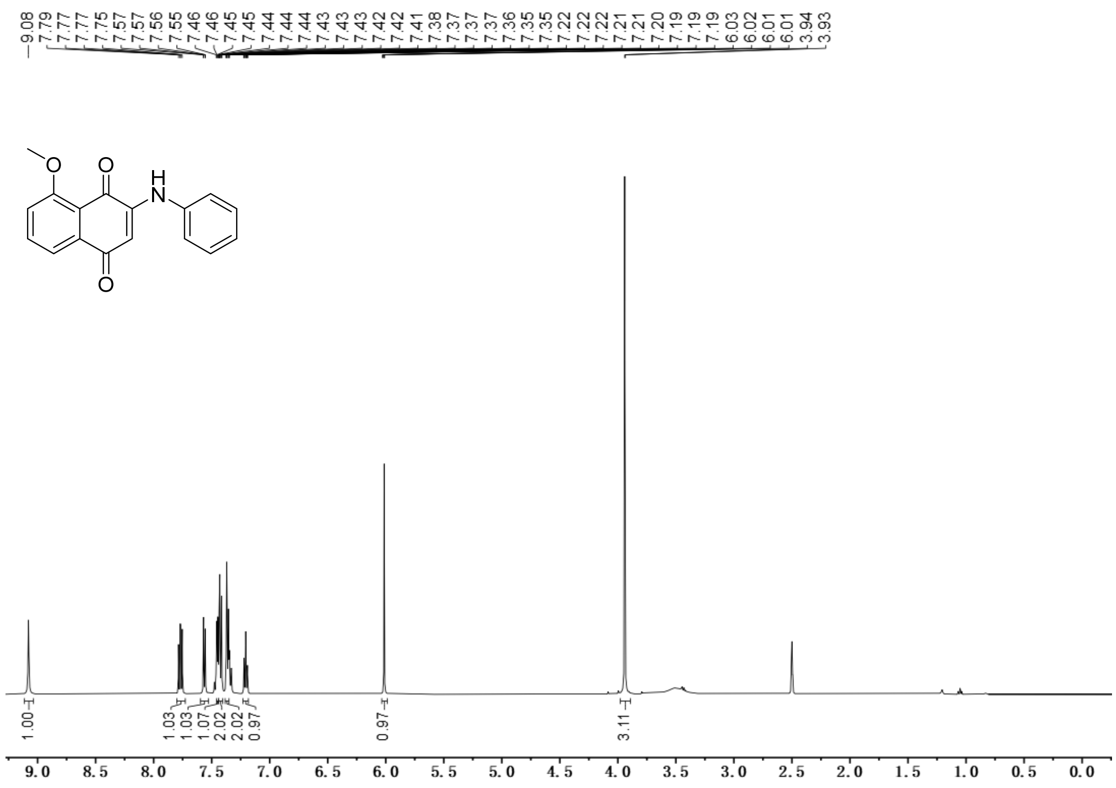

^{1}H NMR Spectra of **4**

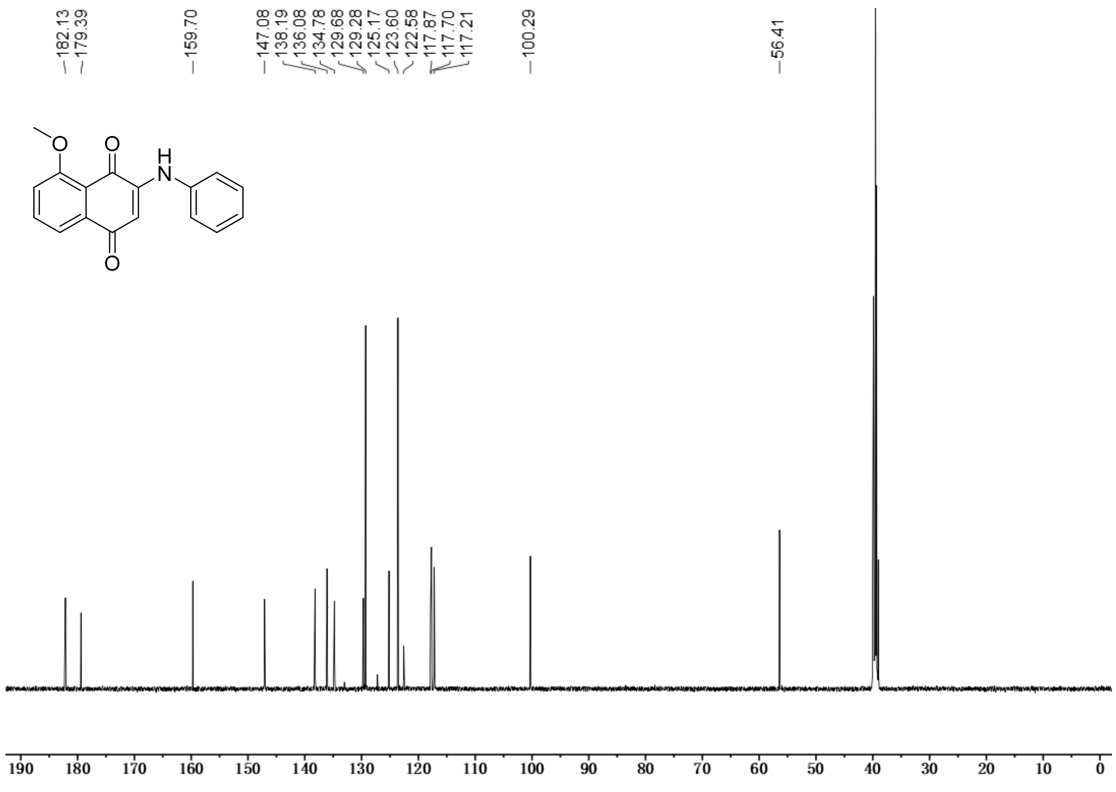

^{13}C NMR Spectra of **4**

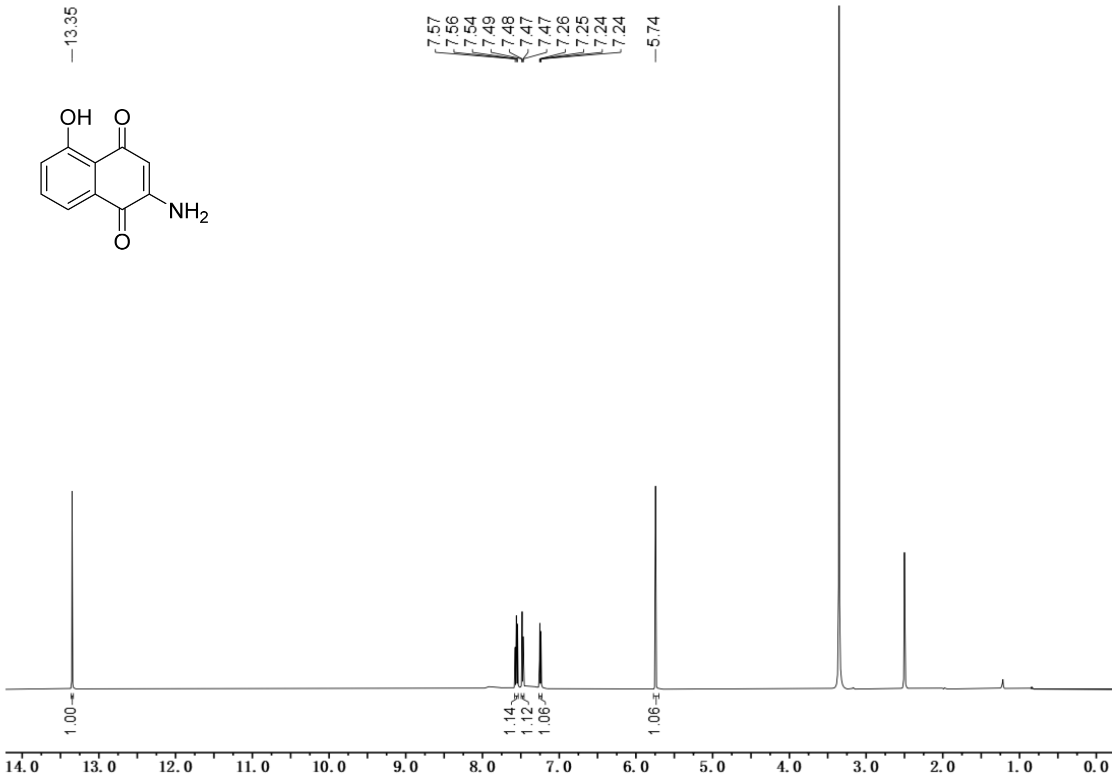

^{1}H NMR Spectra of **5**

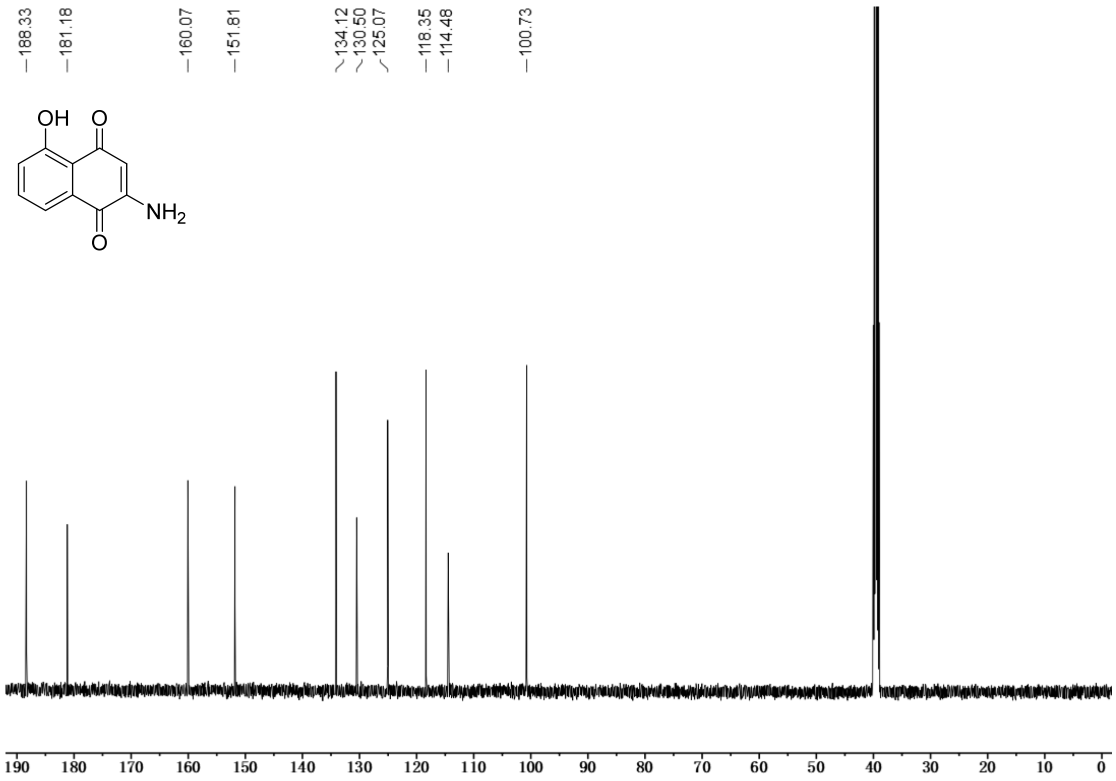

^{13}C NMR Spectra of **5**

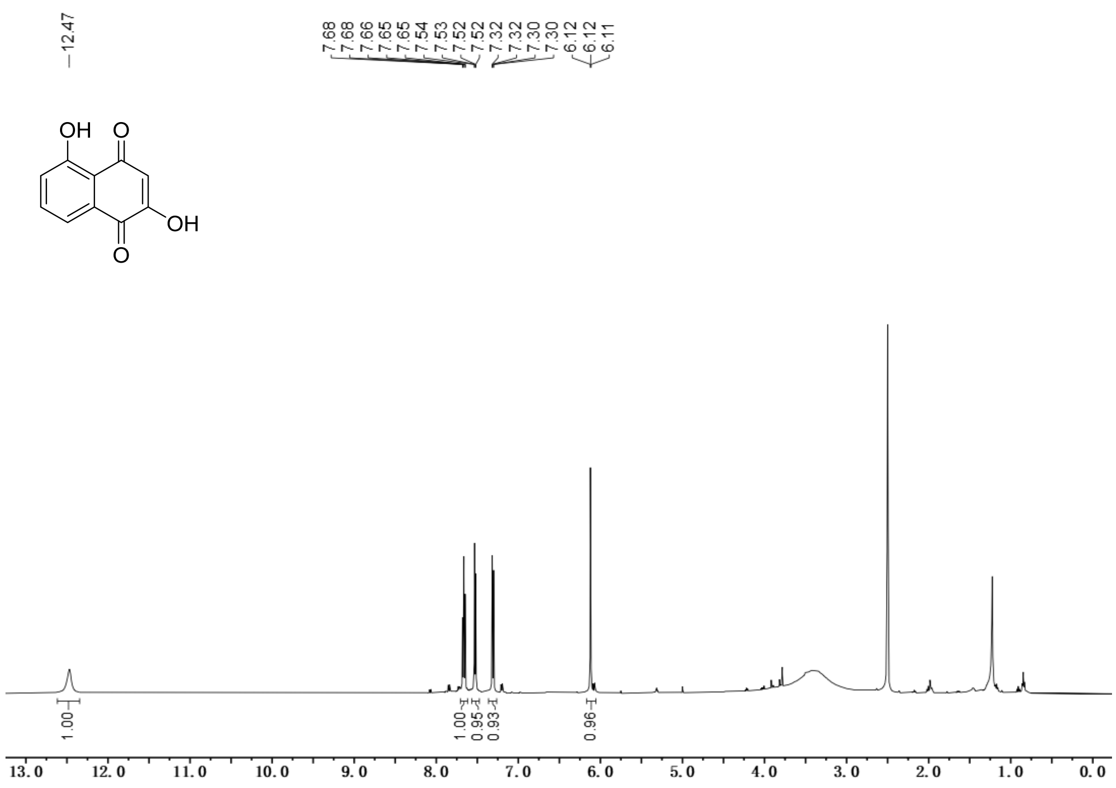

^{1}H NMR Spectra of **6**

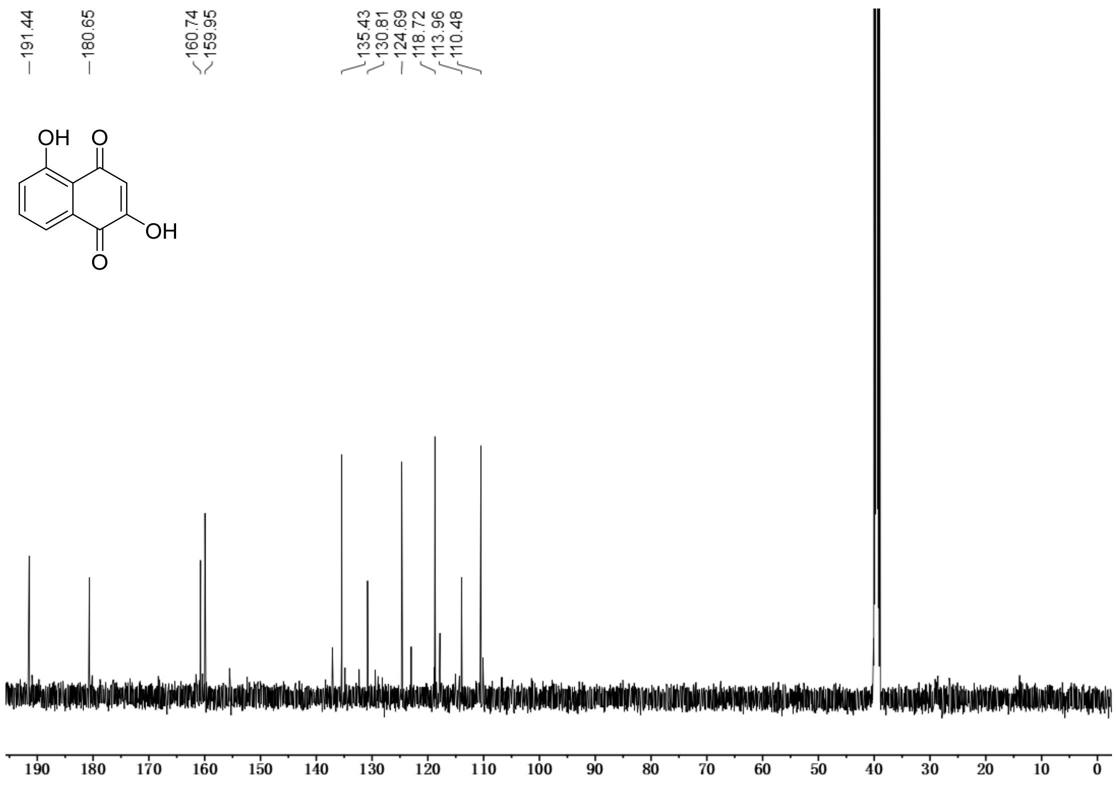

^{13}C NMR Spectra of **6**

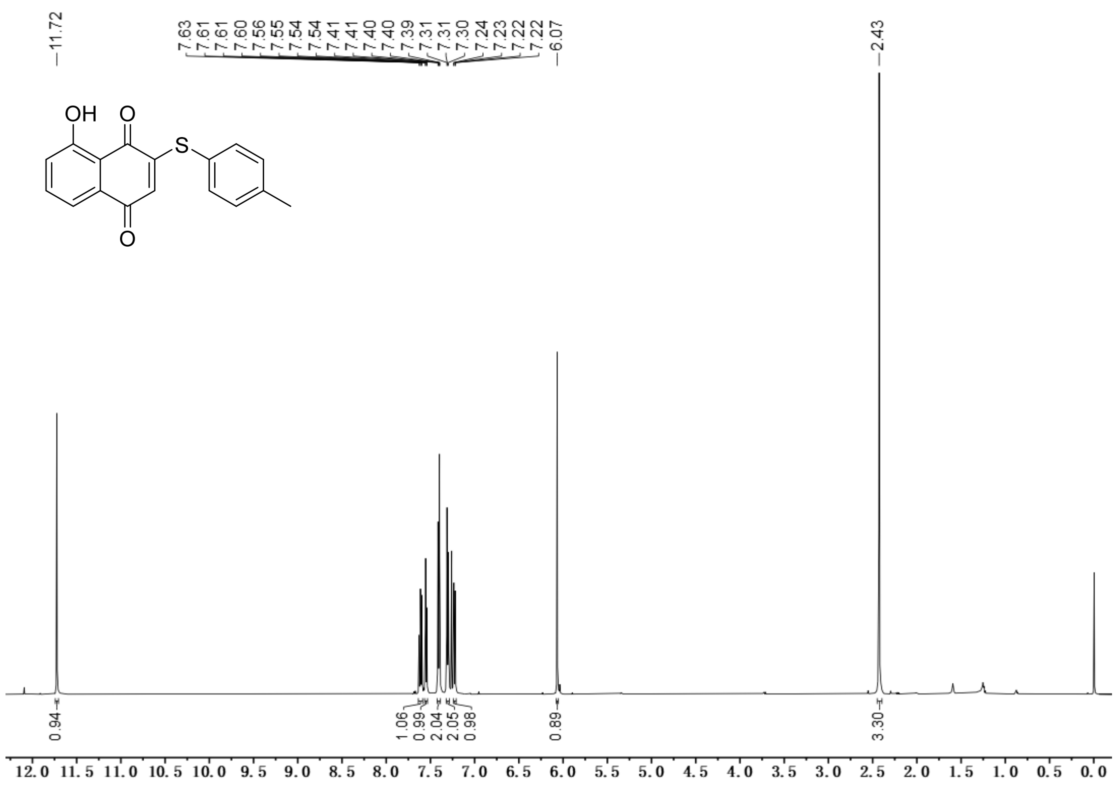

^{1}H NMR Spectra of **7**

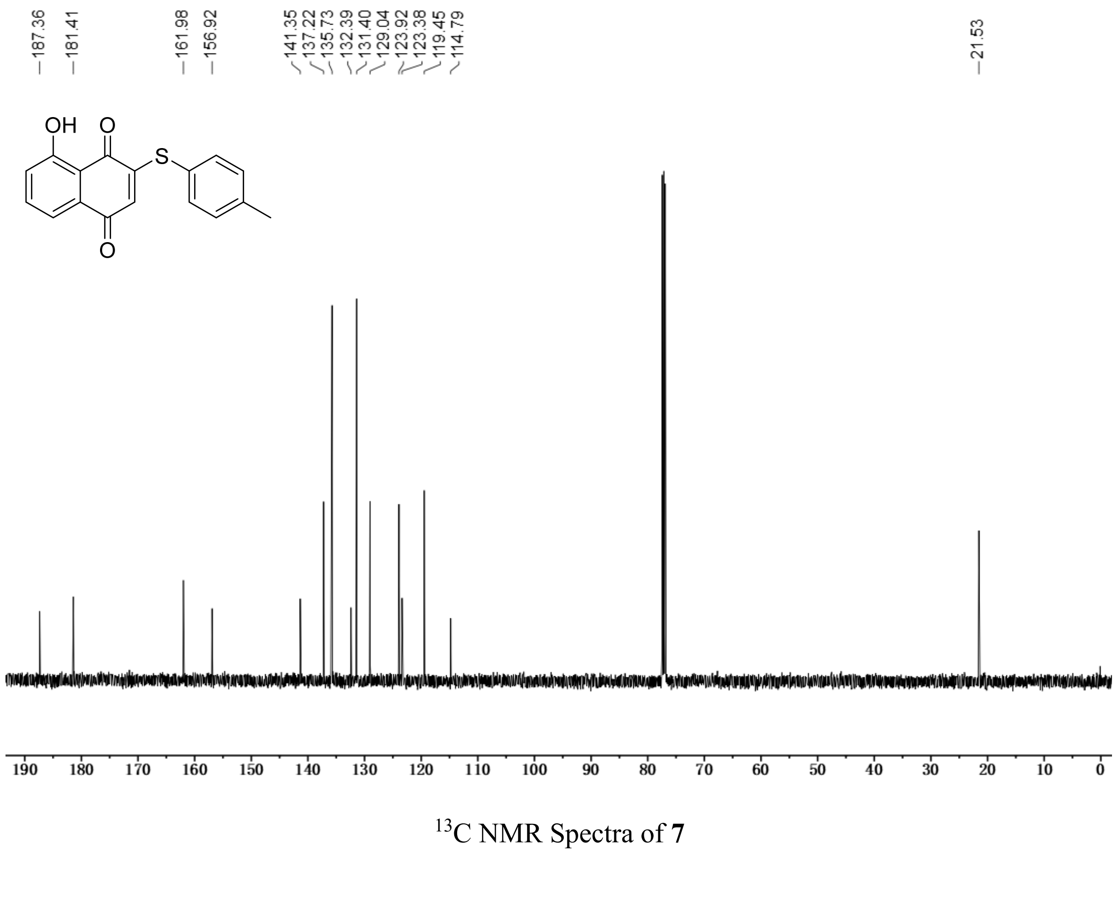

^{13}C NMR Spectra of **7**

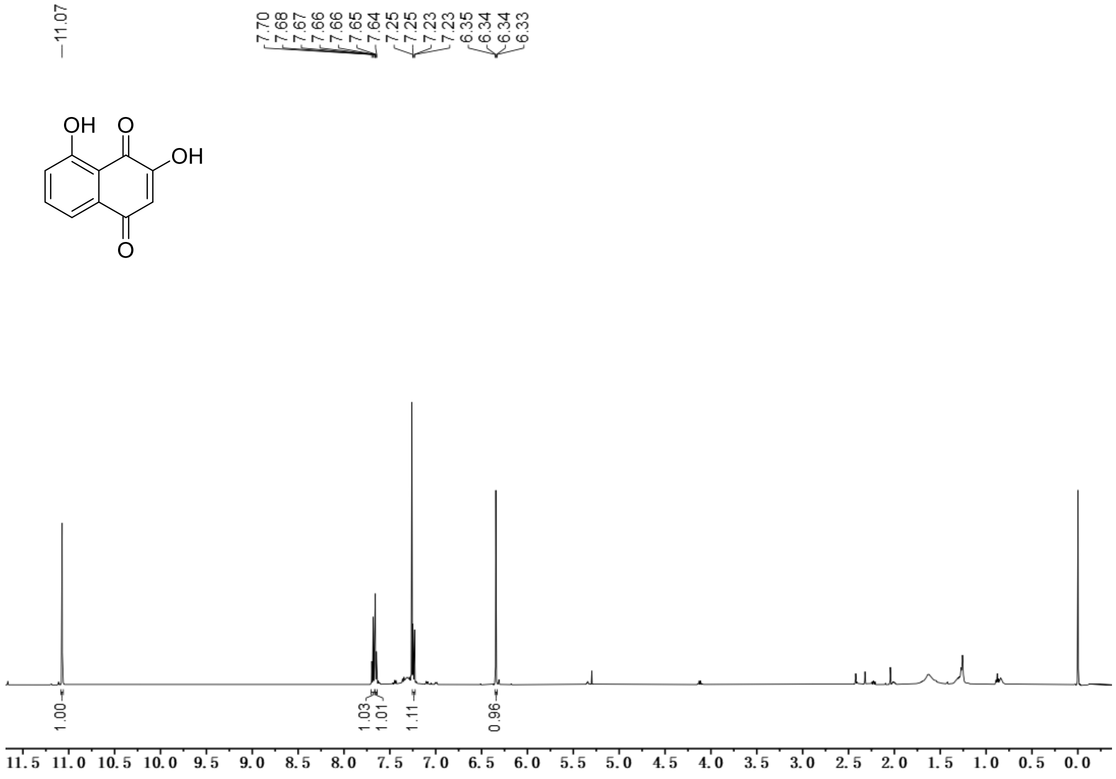

^{1}H NMR Spectra of **8**

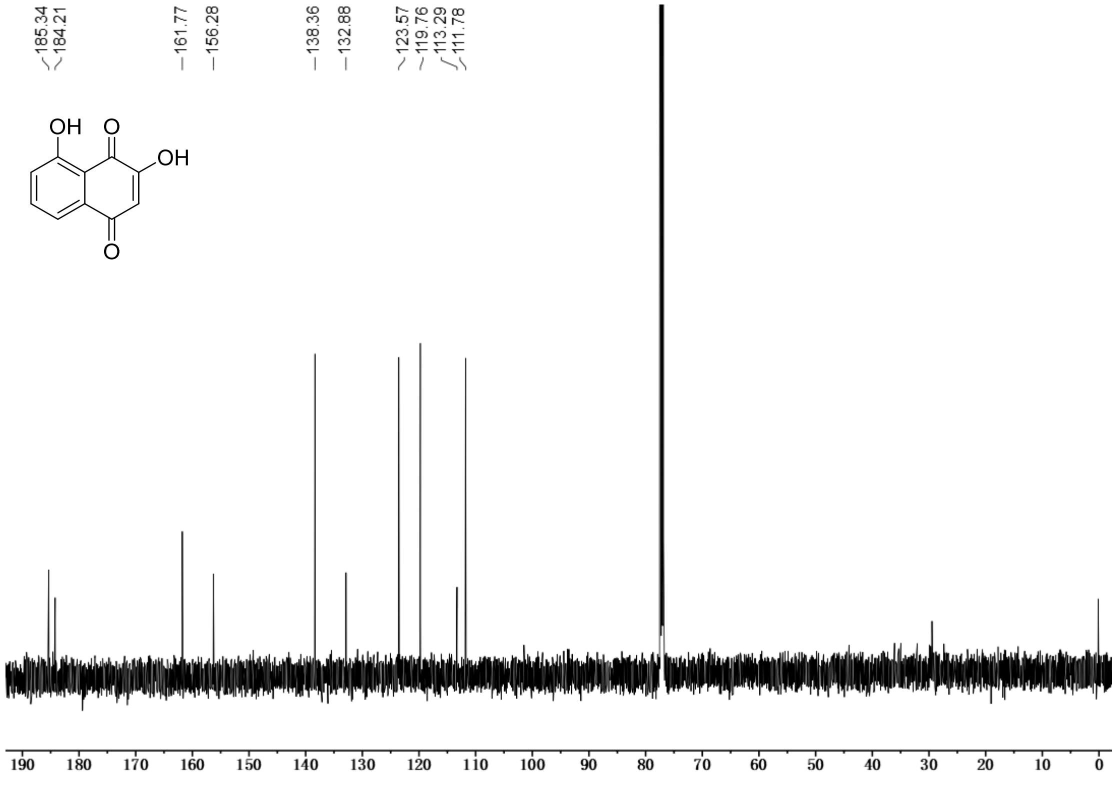

^{13}C NMR Spectra of **8**

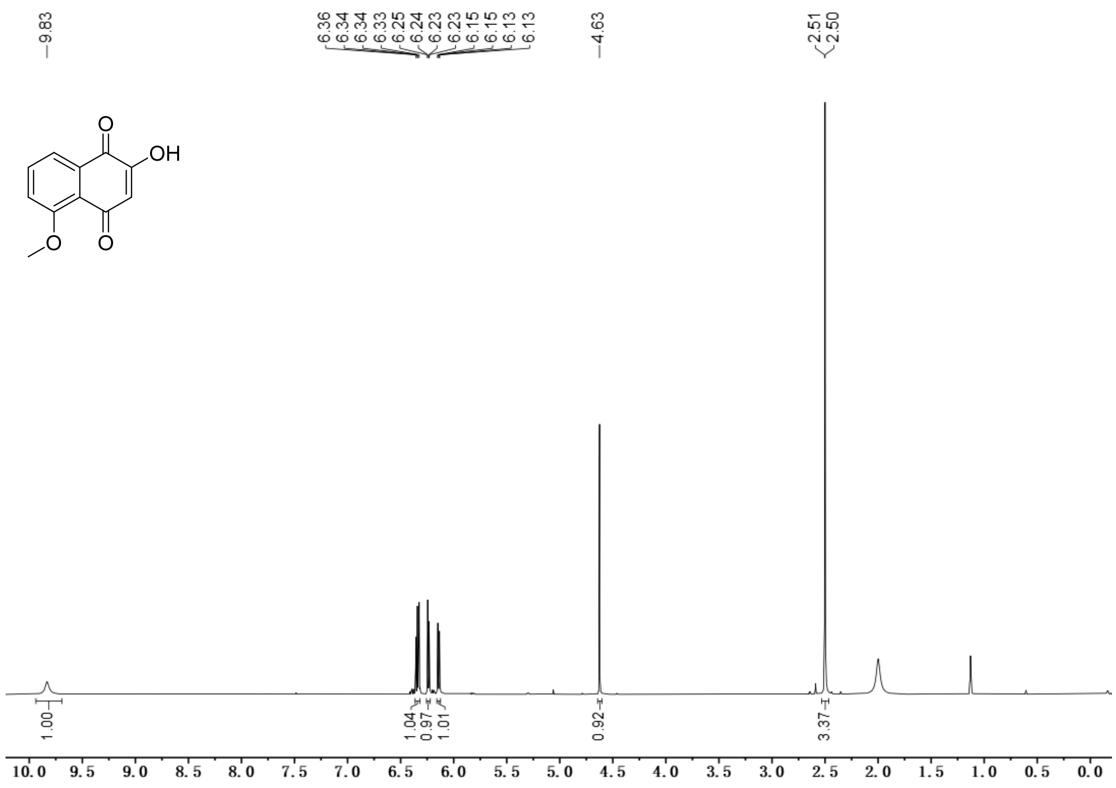

^{1}H NMR Spectra of **9**

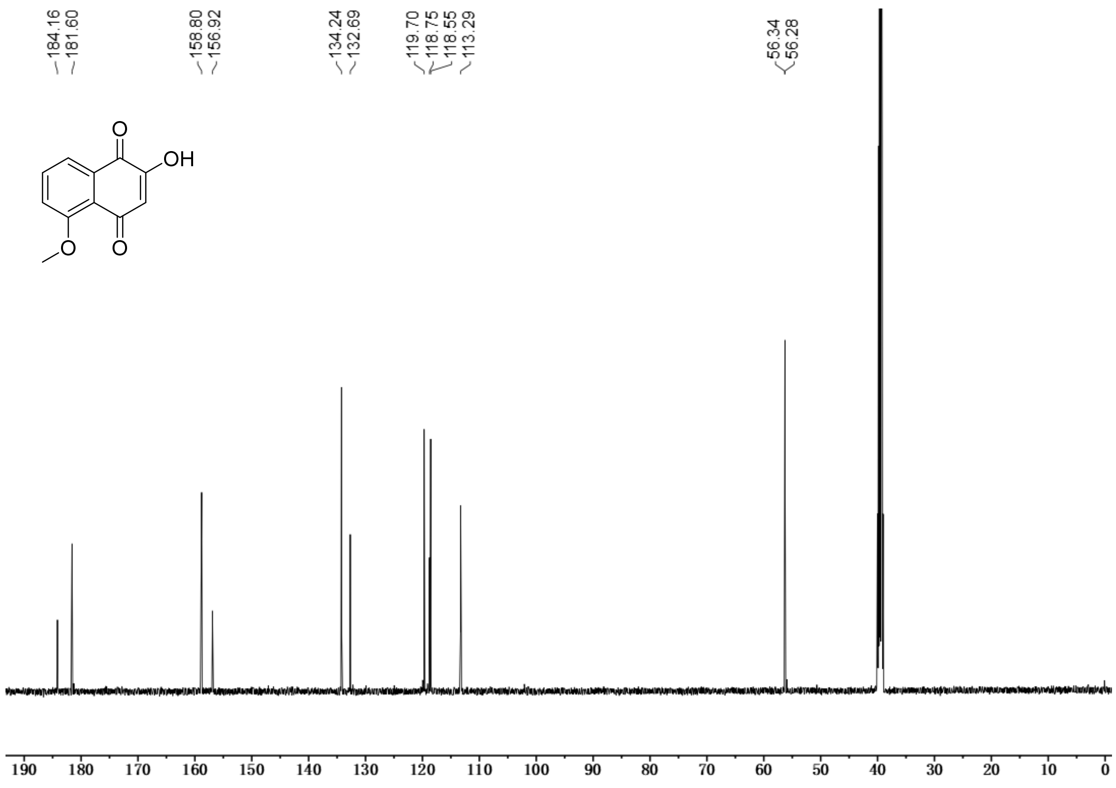

^{13}C NMR Spectra of **9**

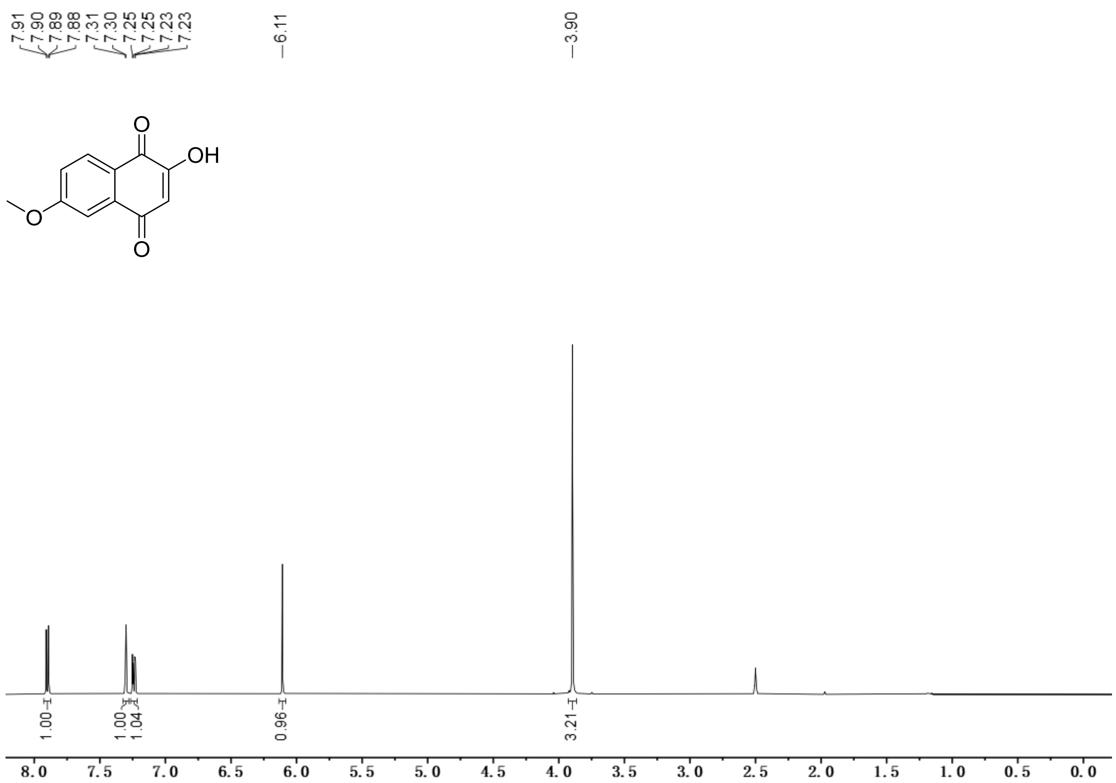

^{1}H NMR Spectra of **10**

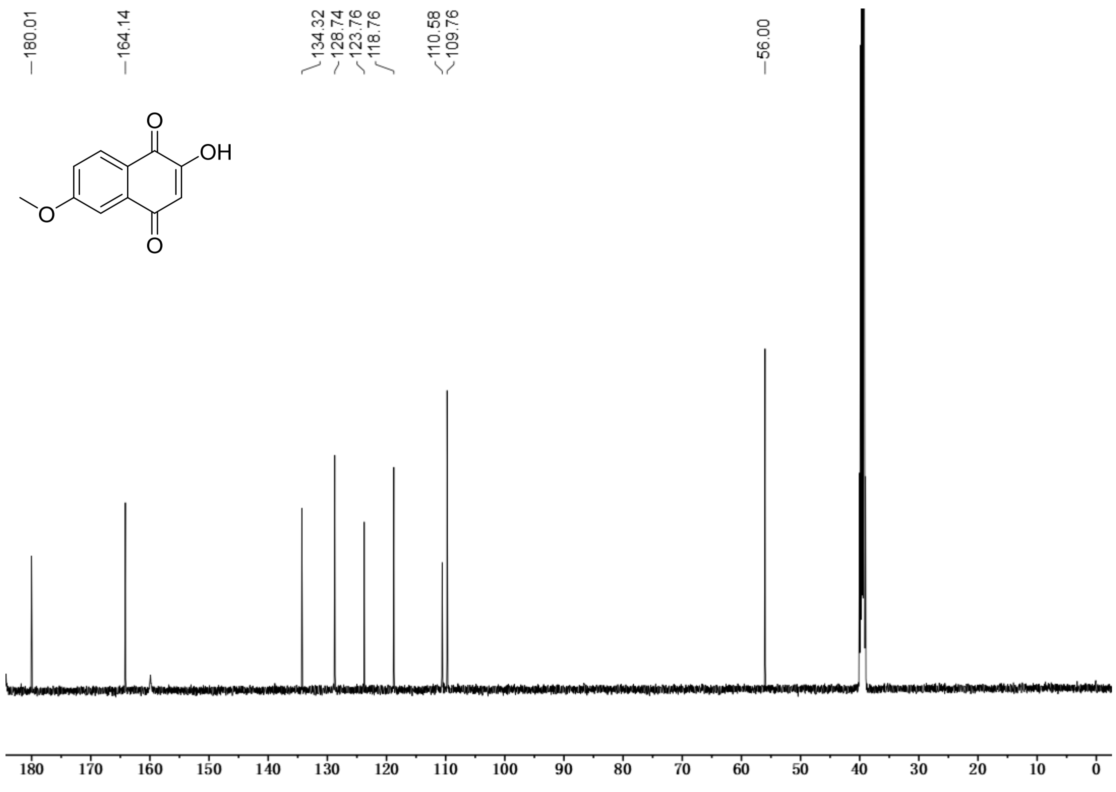

^{13}C NMR Spectra of **10**

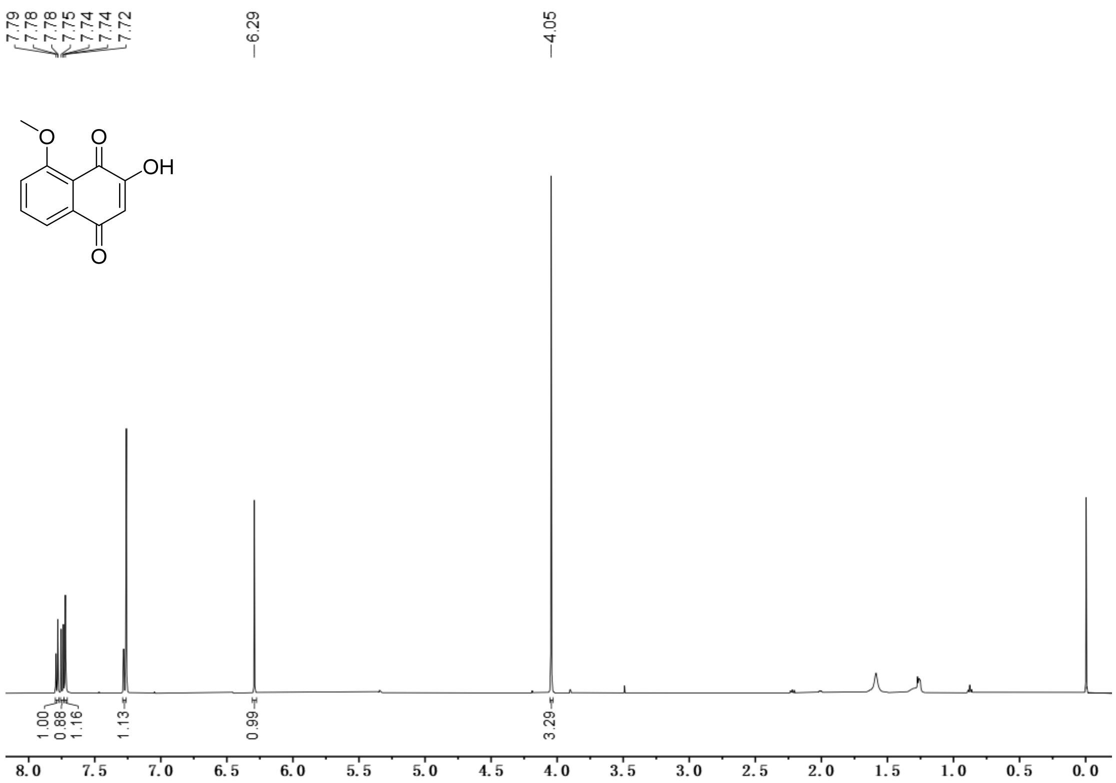

^{1}H NMR Spectra of **11**

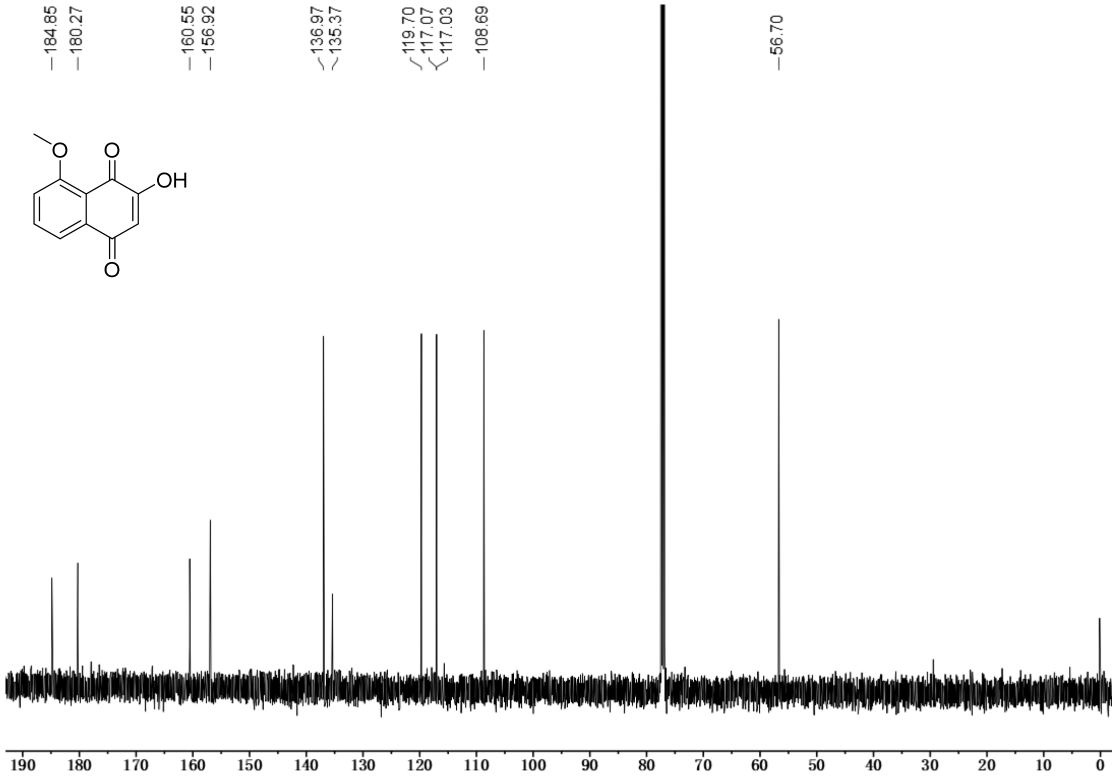

^{13}C NMR Spectra of **11**

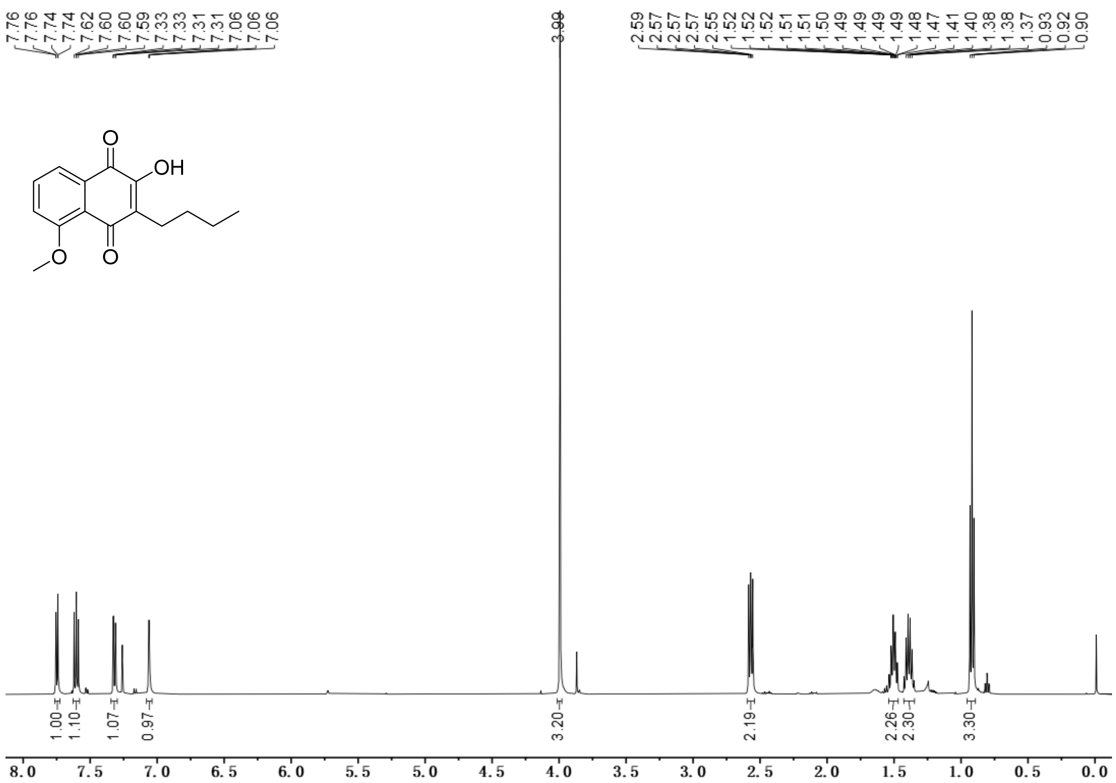

^{1}H NMR Spectra of **12**

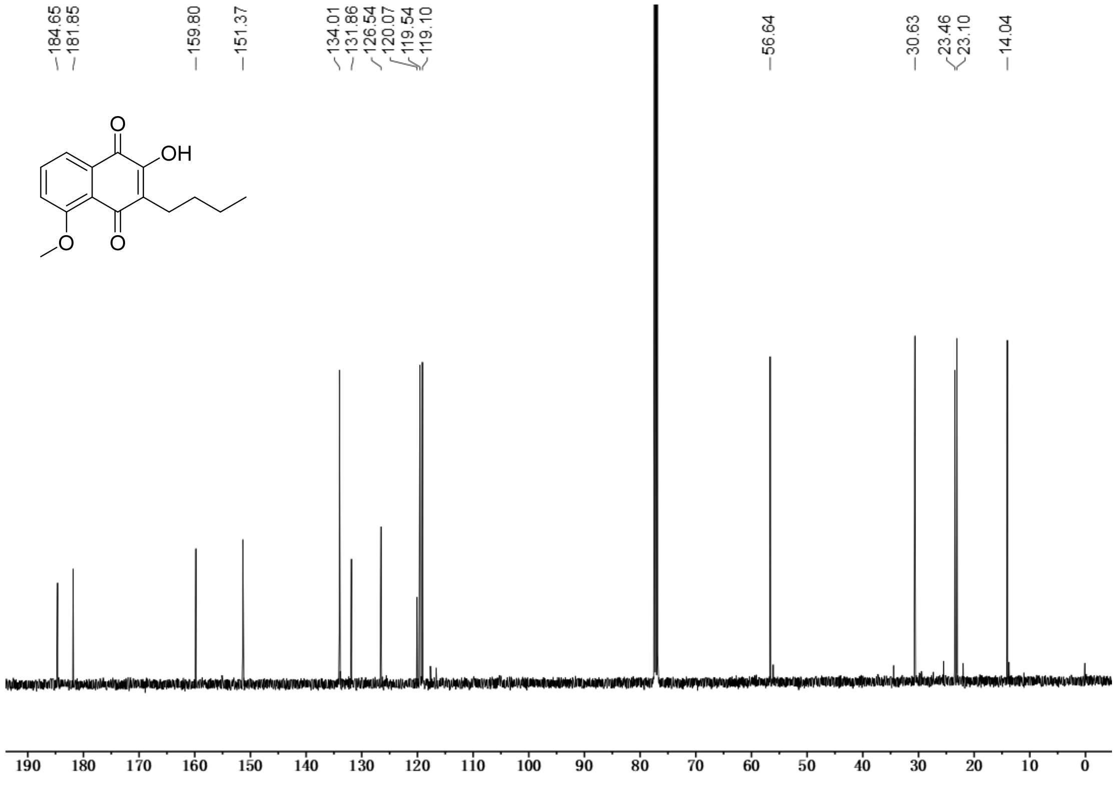

^{13}C NMR Spectra of **12**

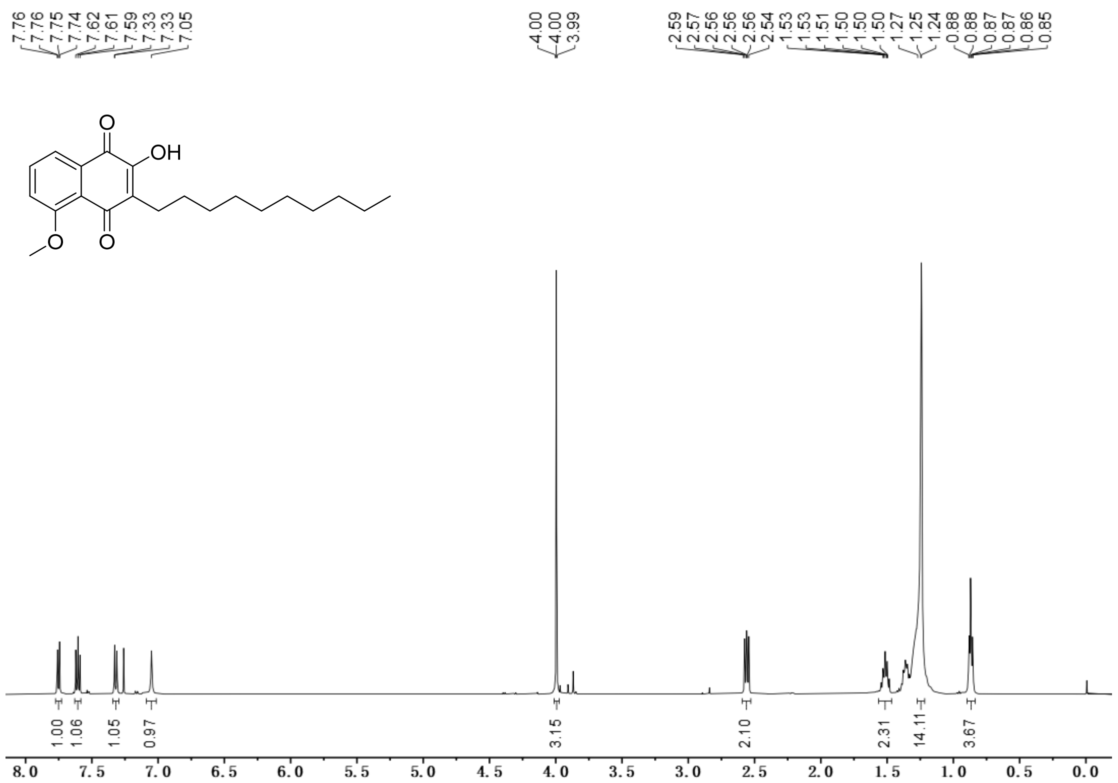

^{1}H NMR Spectra of **13**

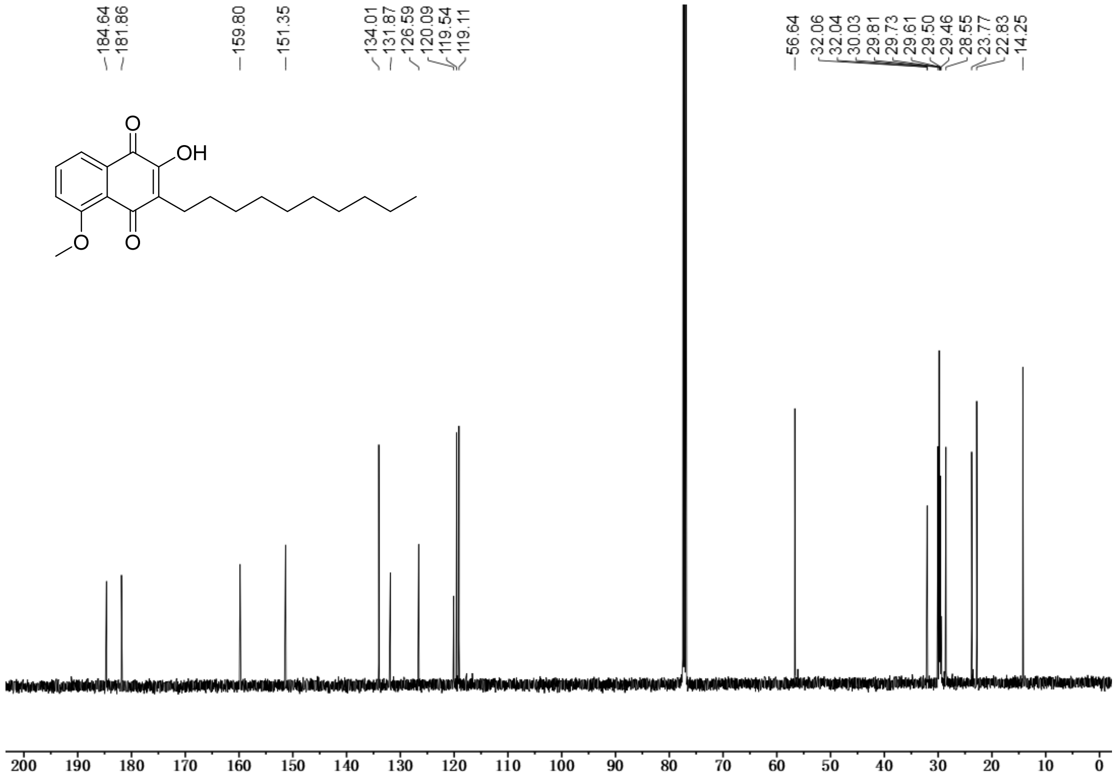

^{13}C NMR Spectra of **13**

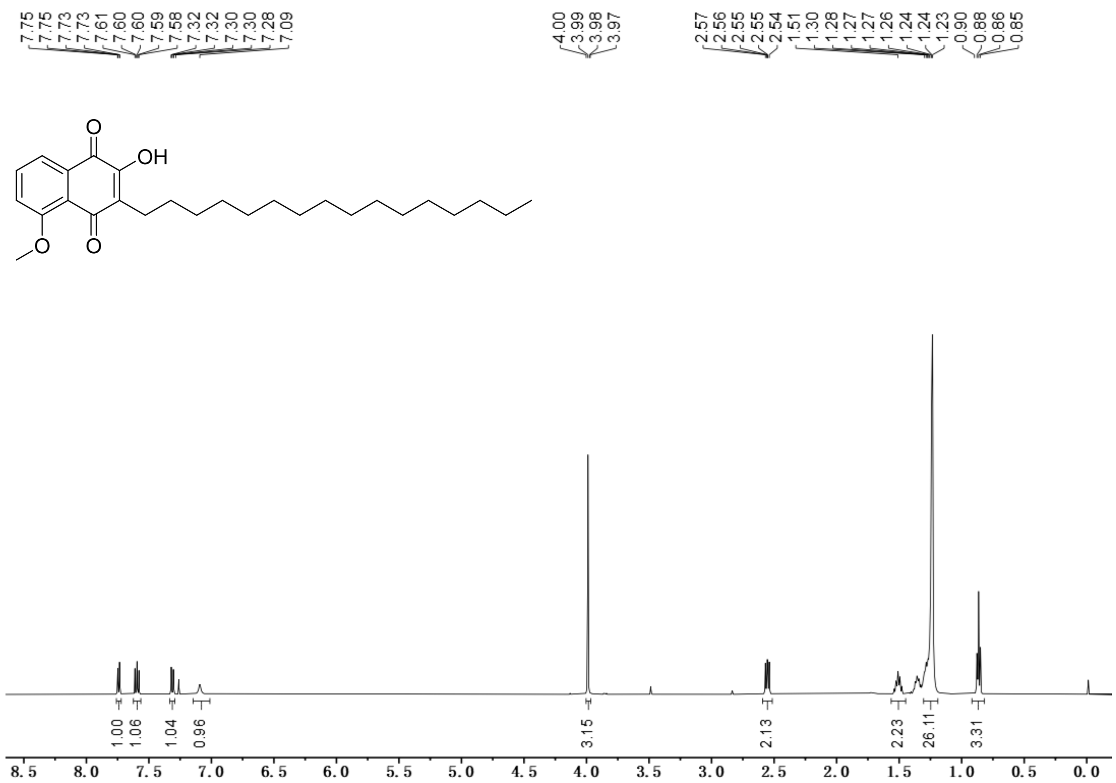

^{1}H NMR Spectra of **14**

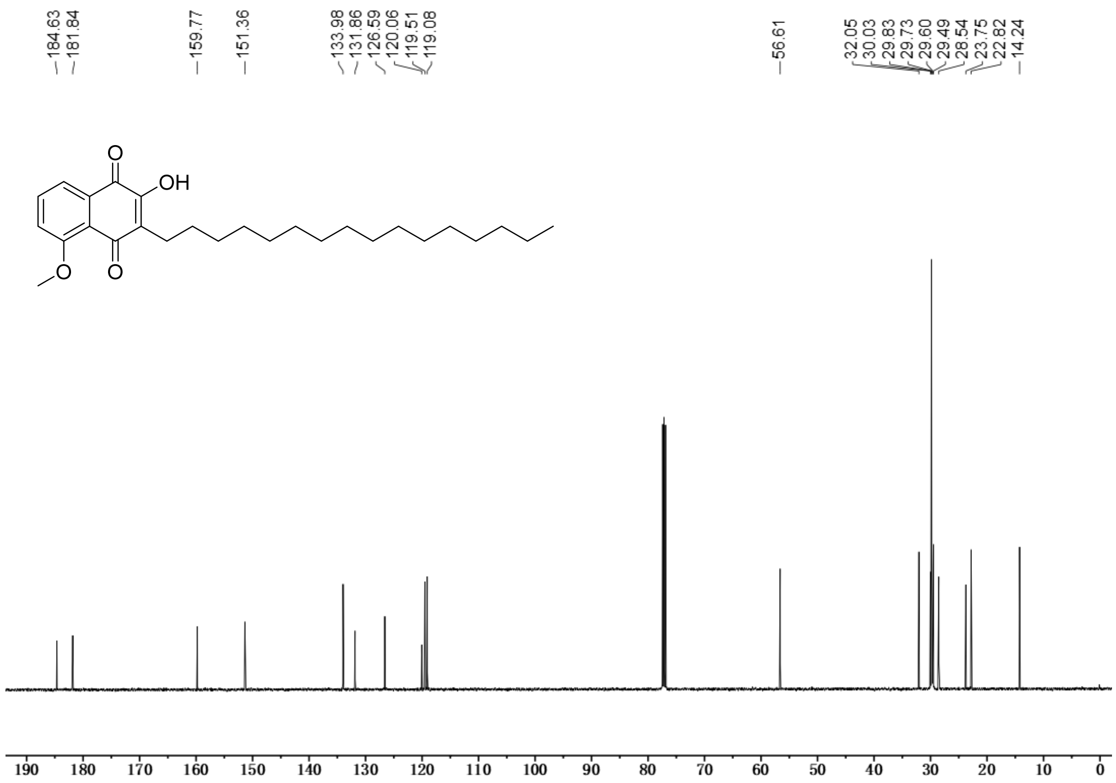

^{13}C NMR Spectra of **14**

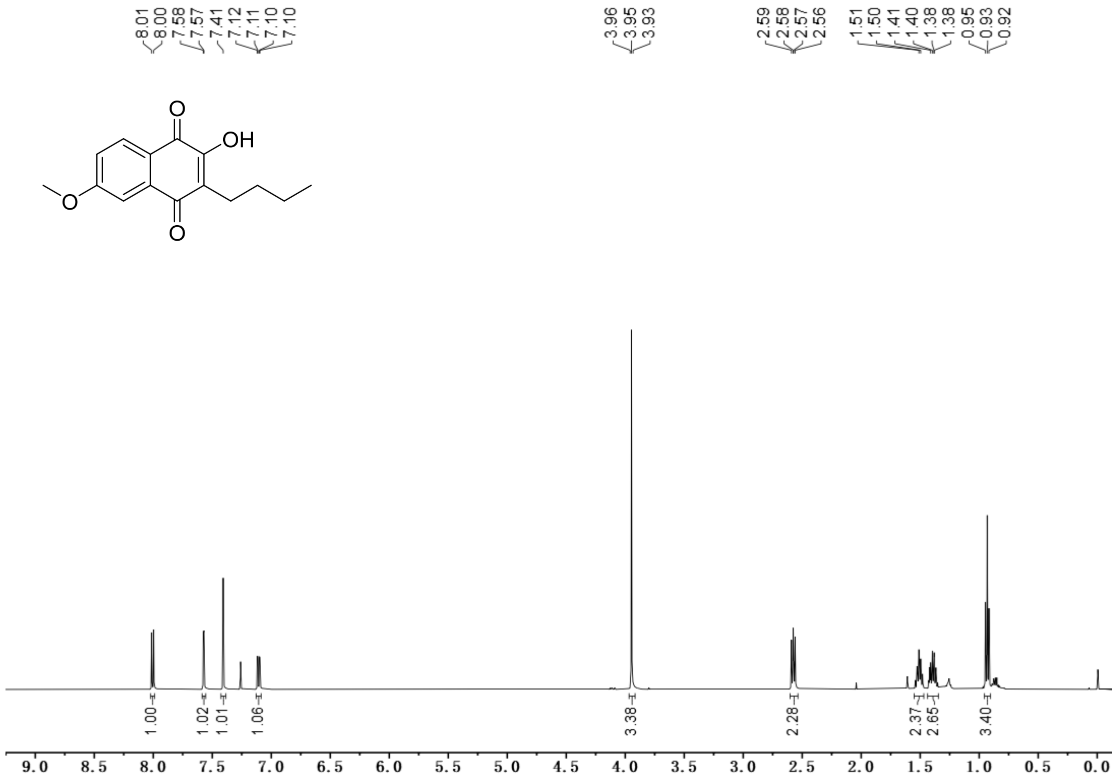

^{1}H NMR Spectra of **15**

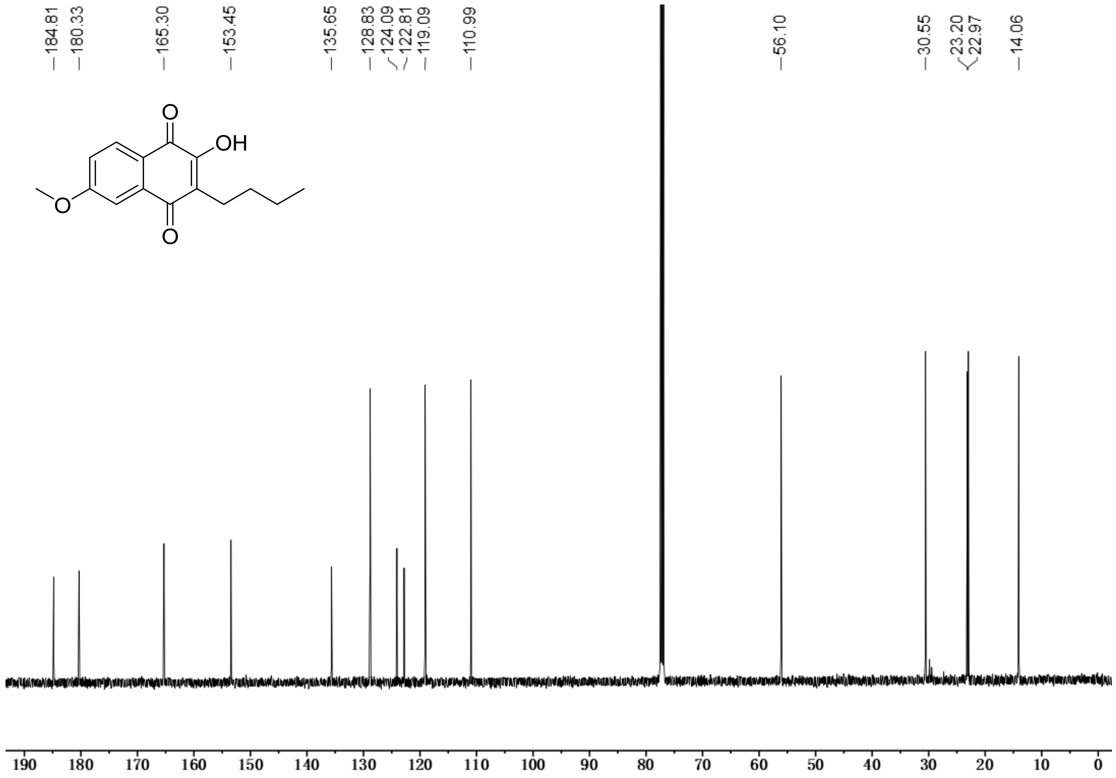

^{13}C NMR Spectra of **15**

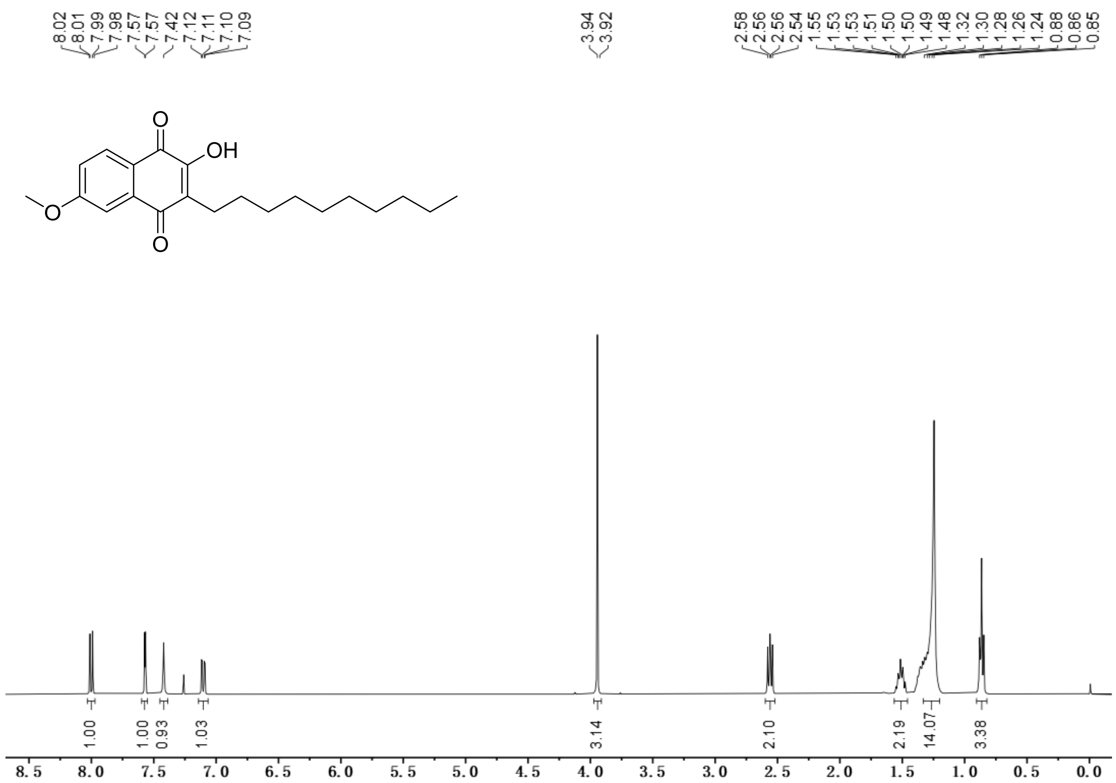

^{1}H NMR Spectra of **16**

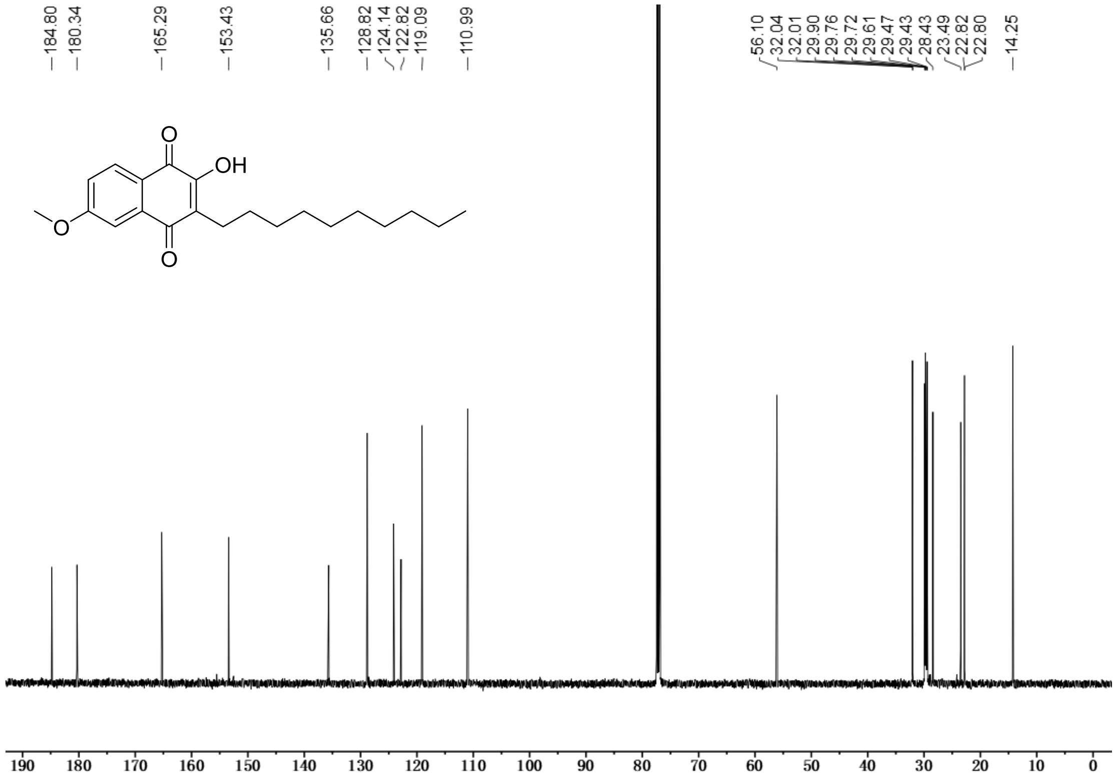

^{13}C NMR Spectra of **16**

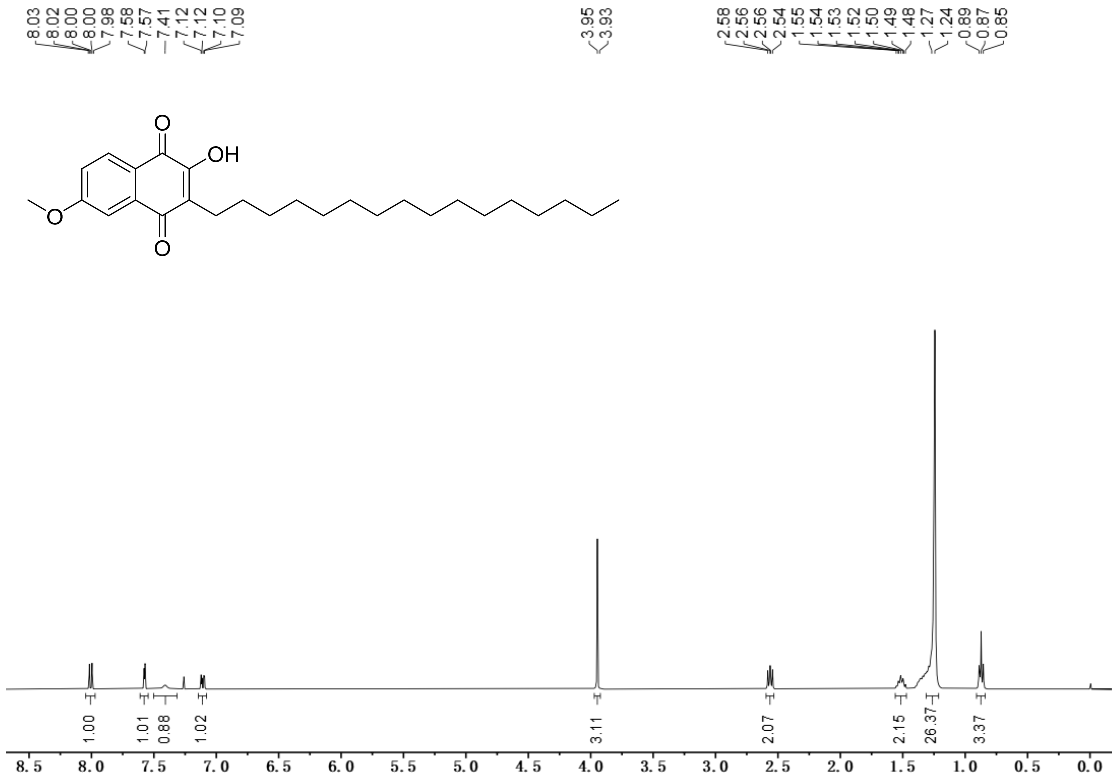

^{1}H NMR Spectra of **17**

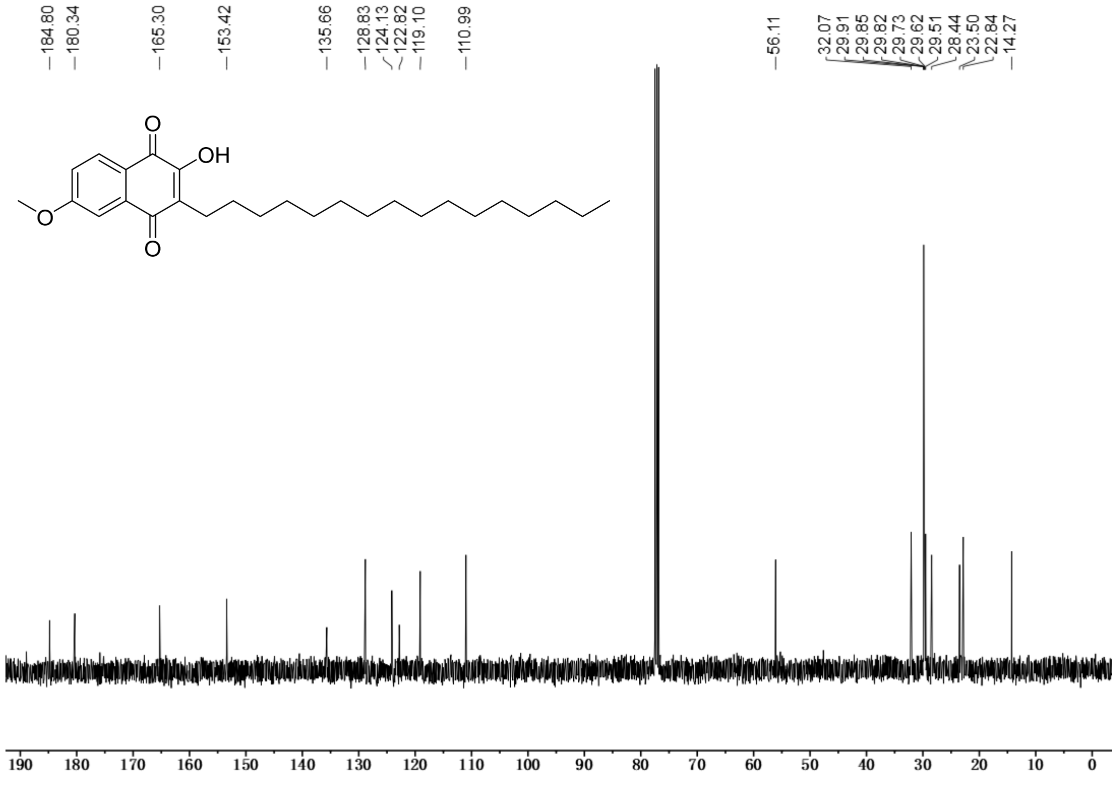

^{13}C NMR Spectra of **17**

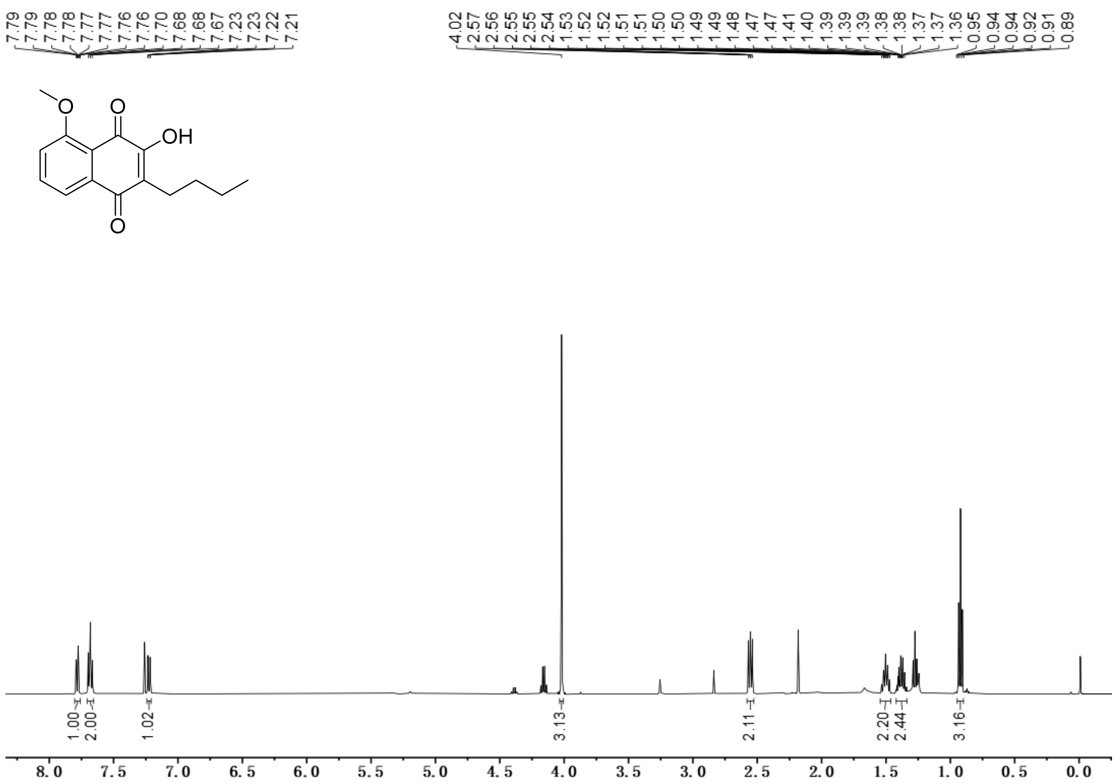

^{1}H NMR Spectra of **18**

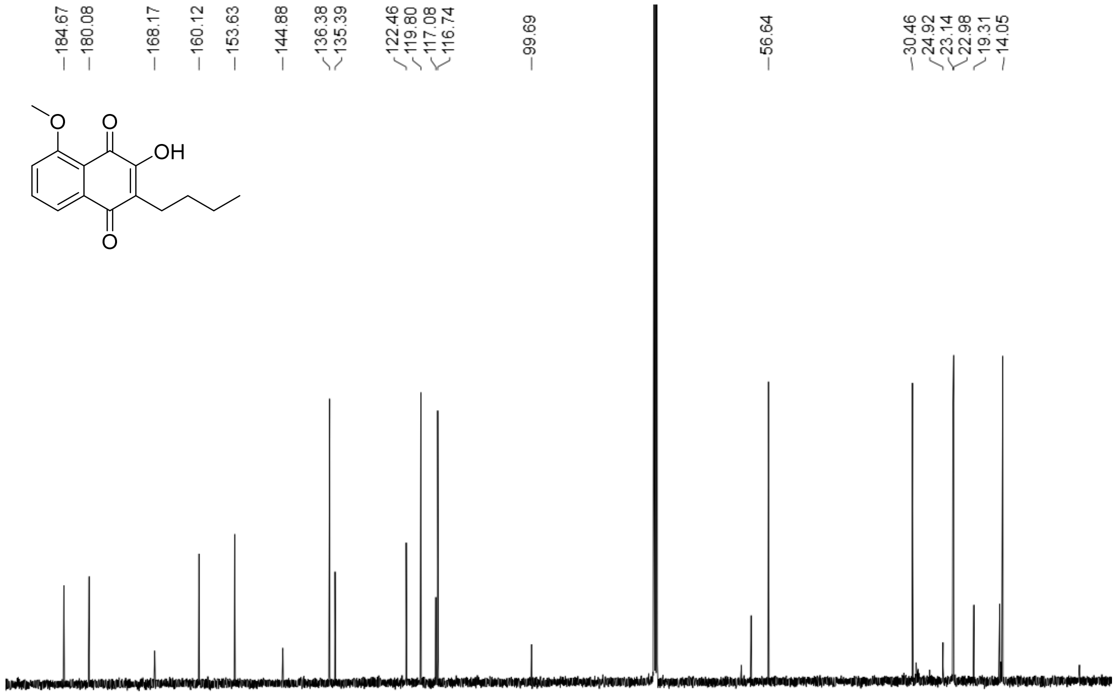

^{13}C NMR Spectra of **18**

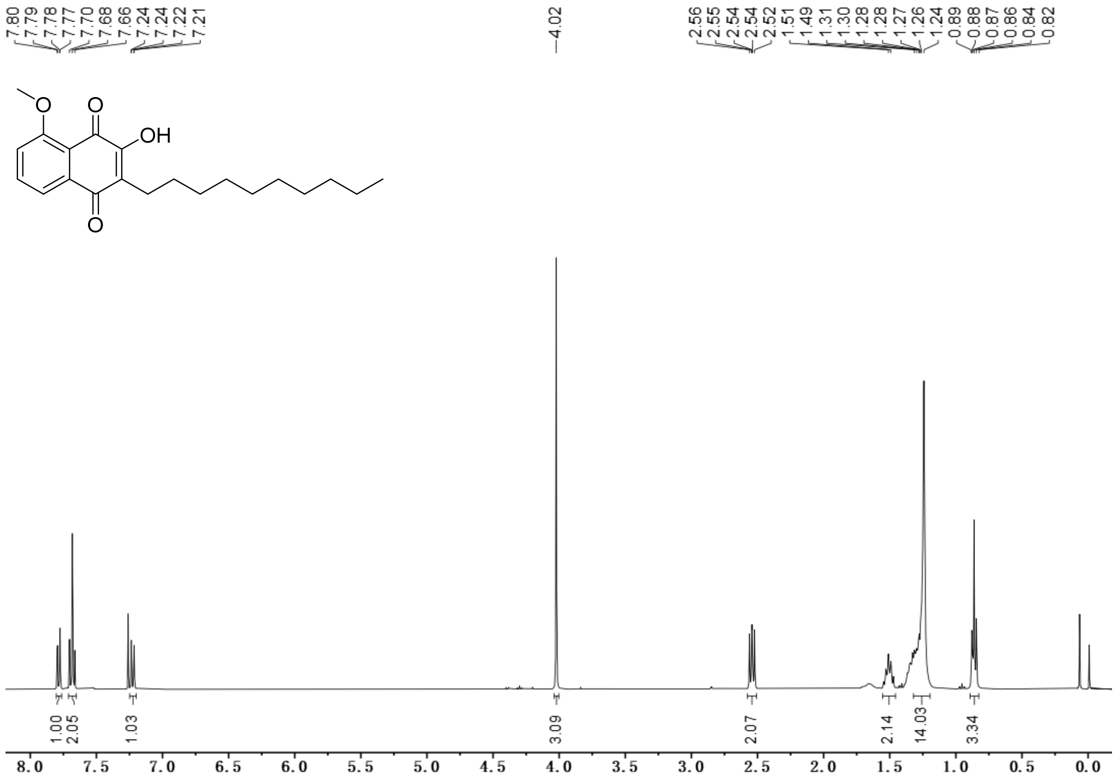

^{1}H NMR Spectra of **19**

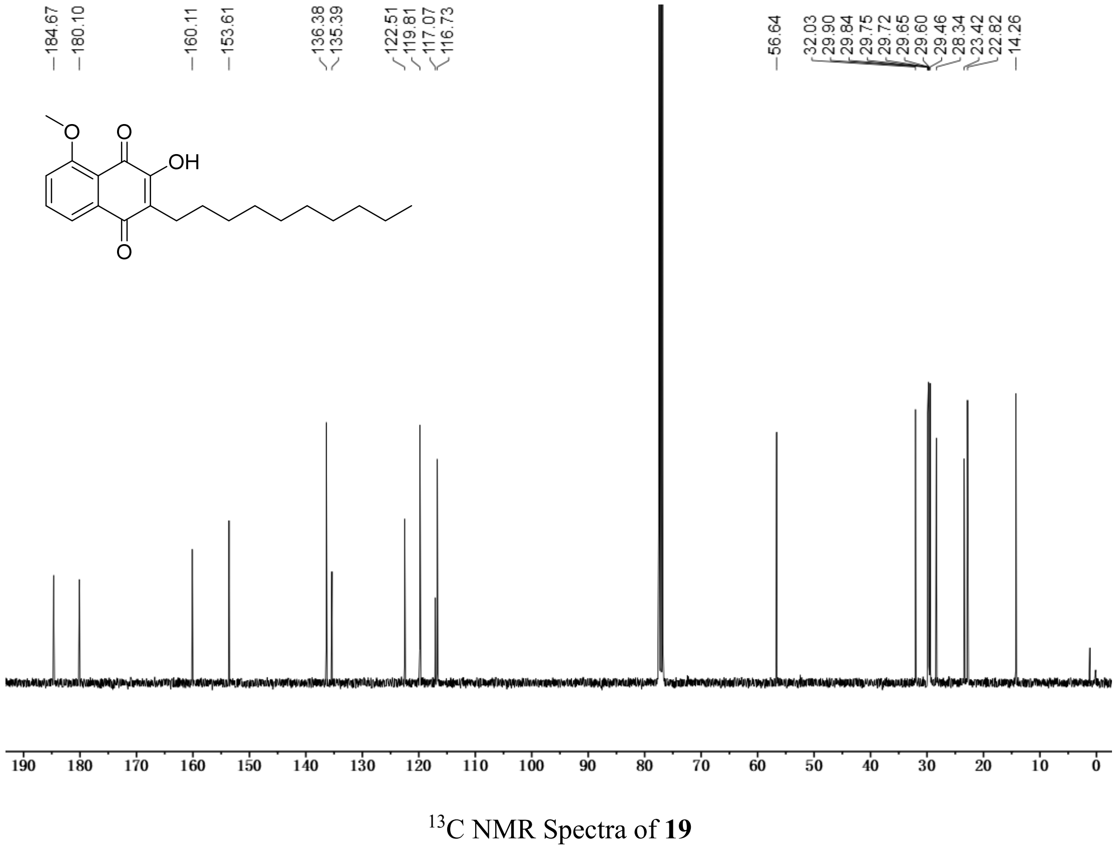

^{13}C NMR Spectra of **19**

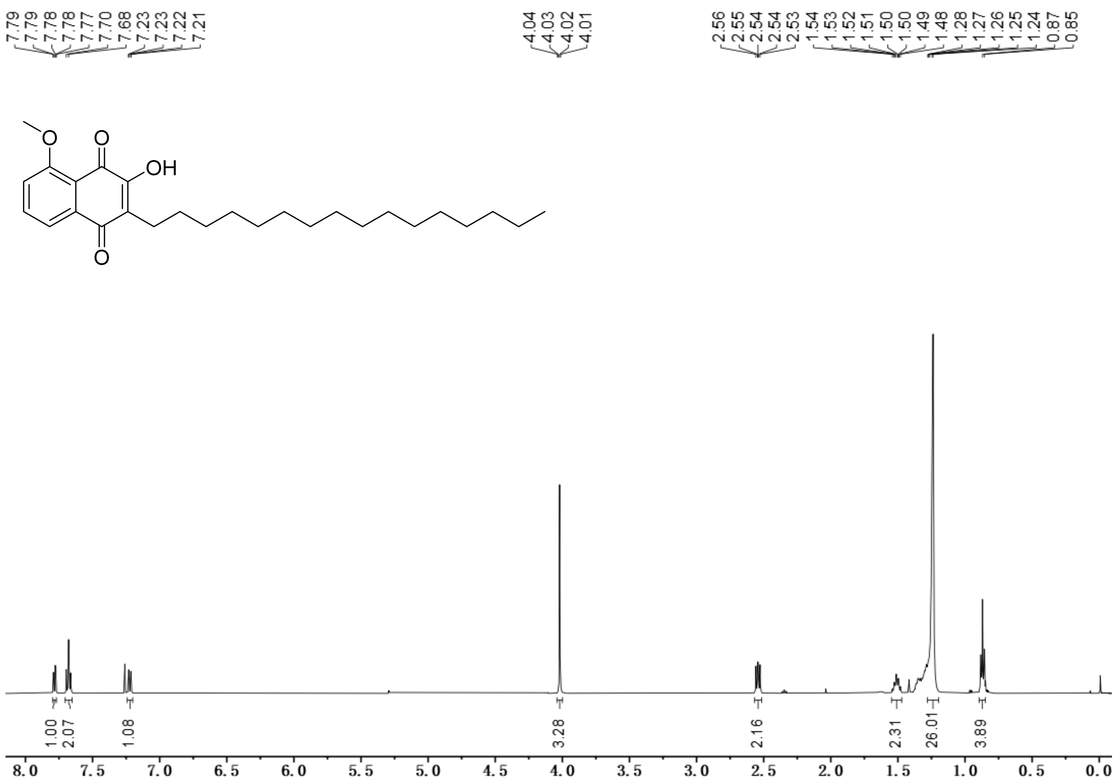

^{1}H NMR Spectra of **20**

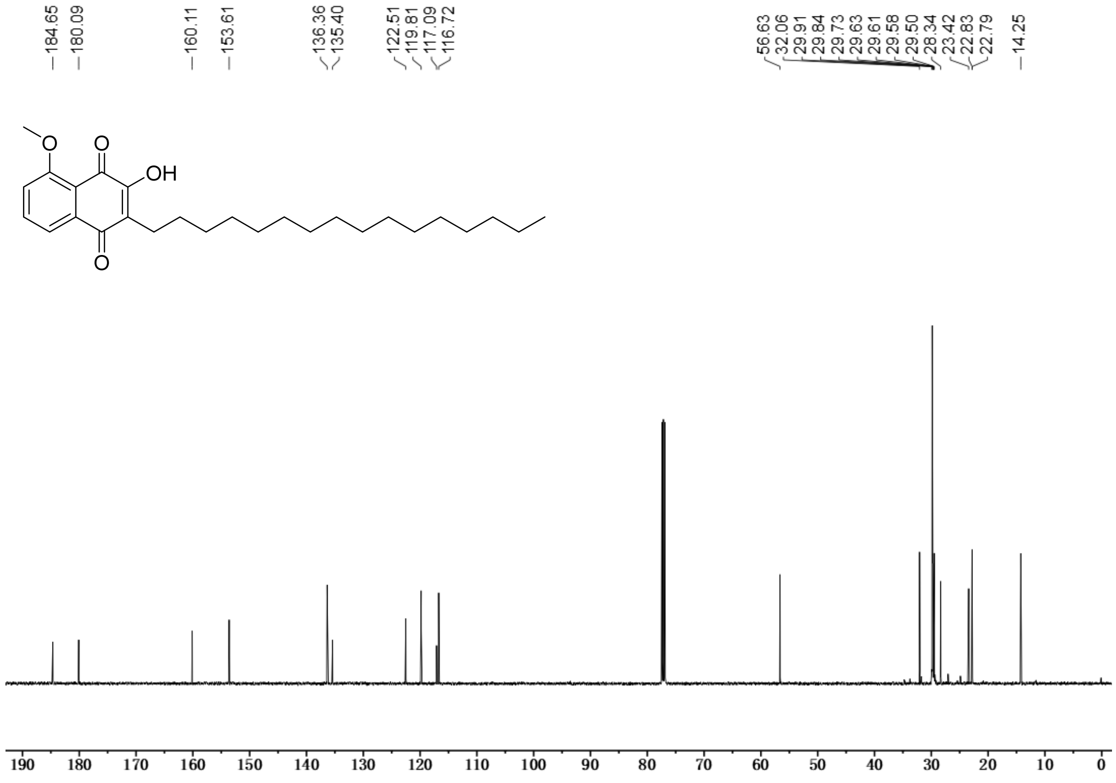

^{13}C NMR Spectra of **20**

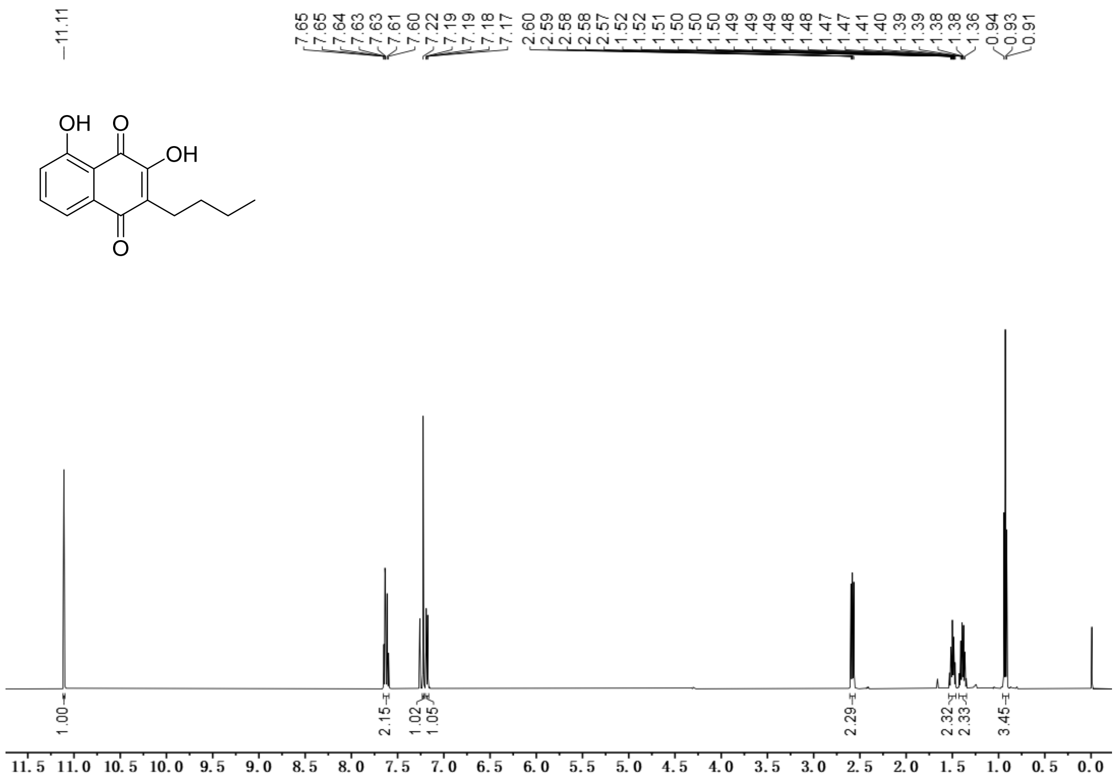

^{1}H NMR Spectra of **21**

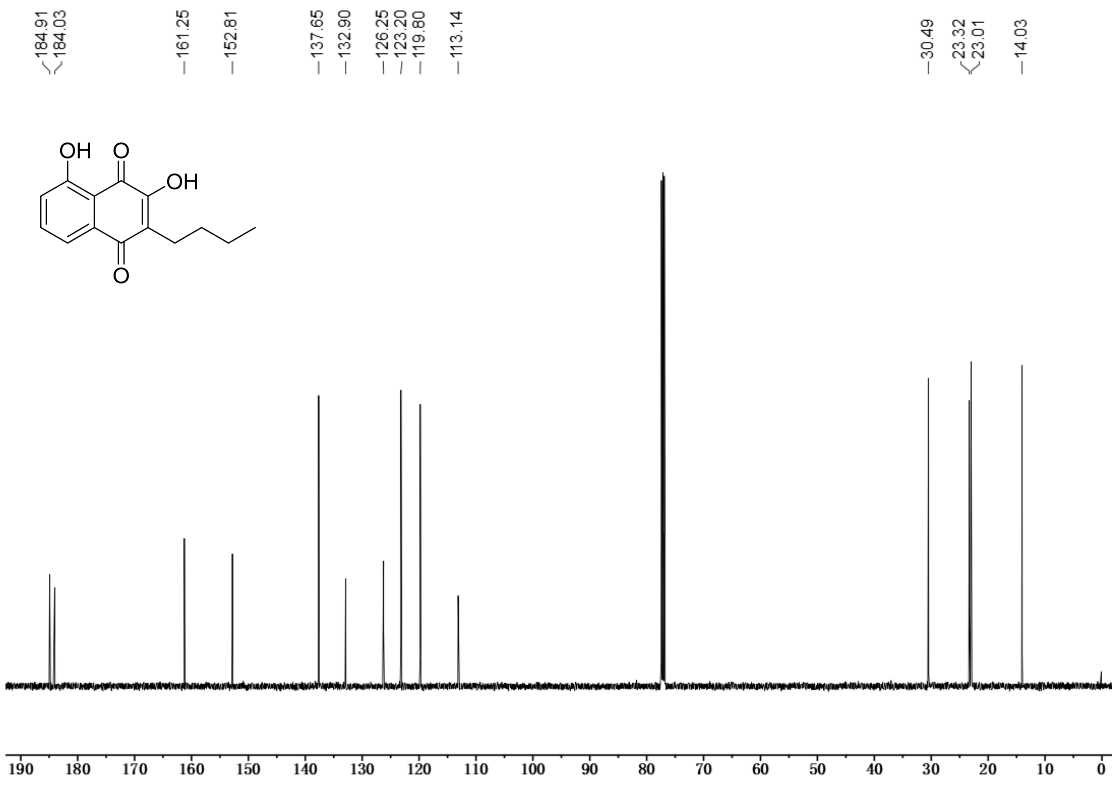

^{13}C NMR Spectra of **21**

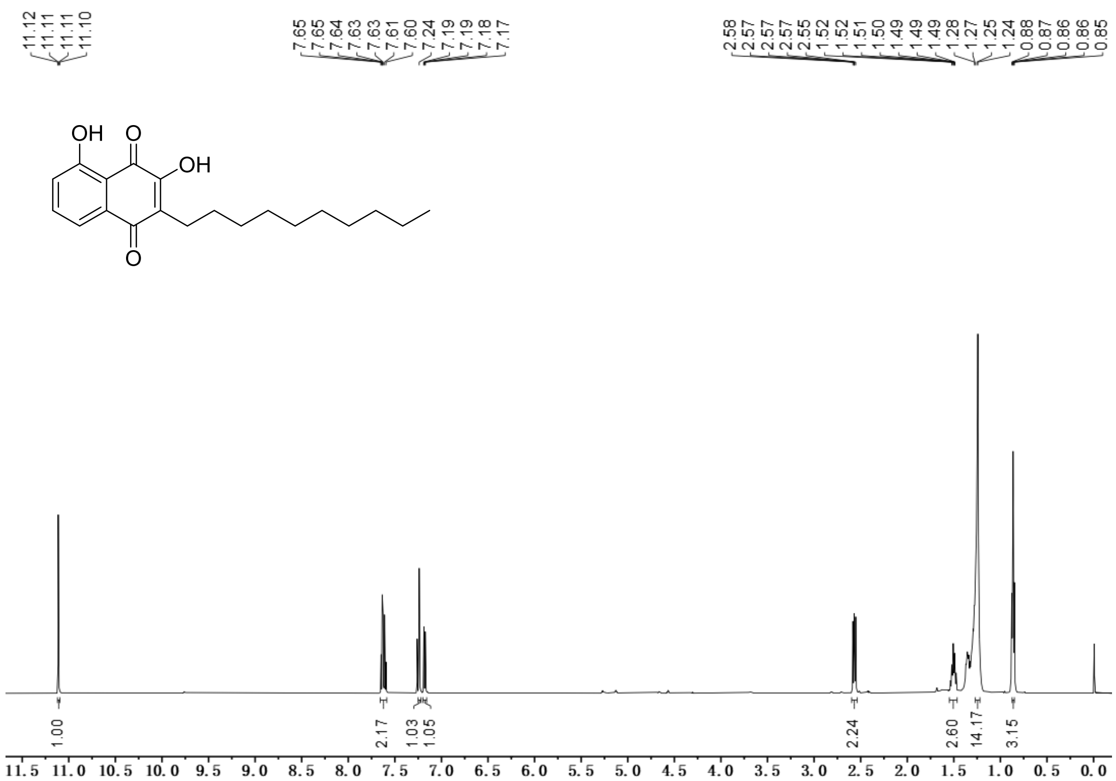

^{1}H NMR Spectra of **22**

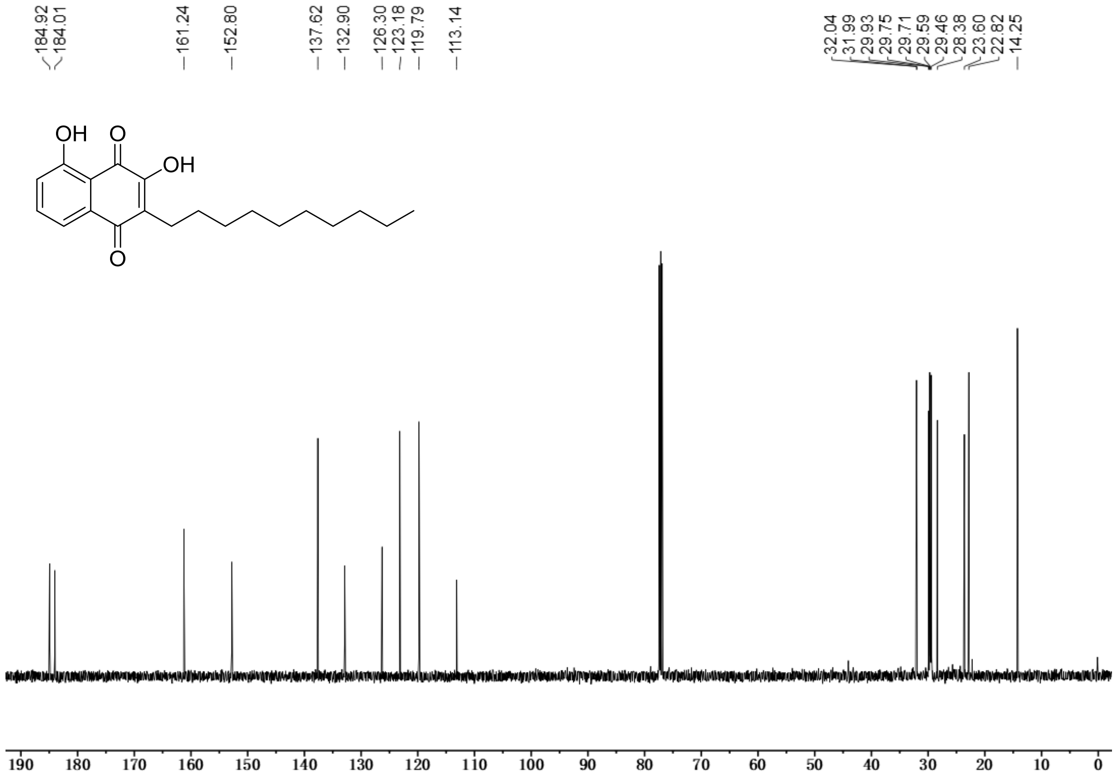

^{13}C NMR Spectra of **22**

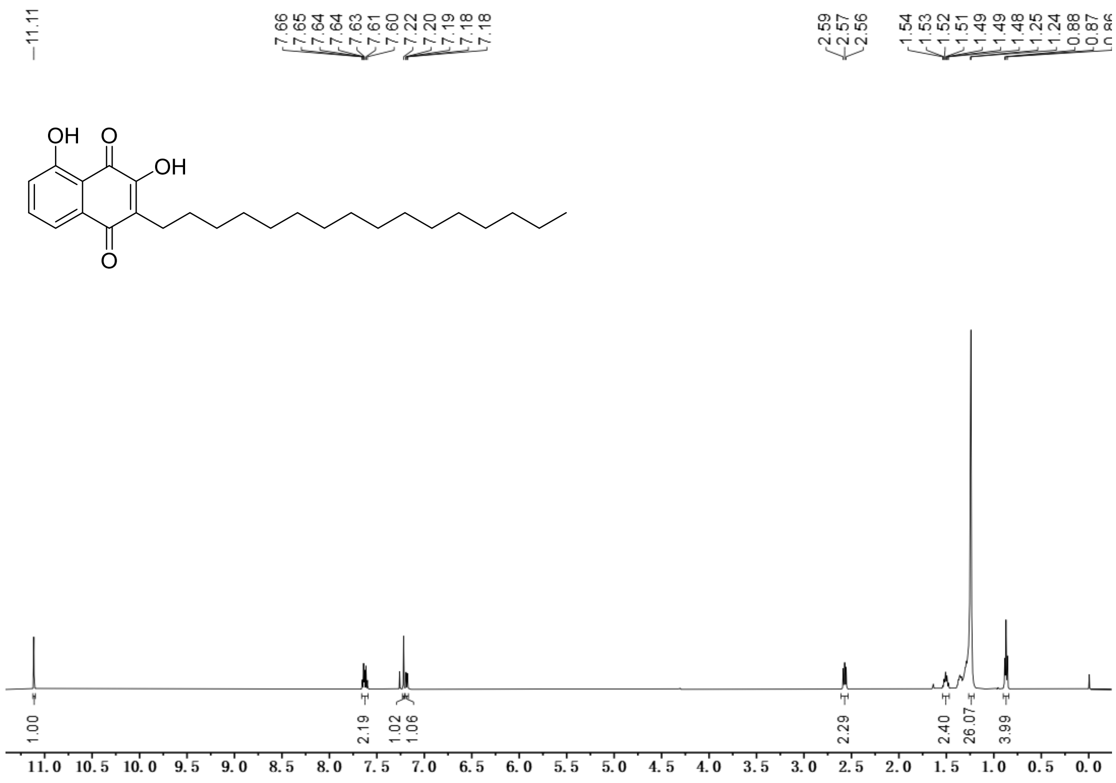

^{1}H NMR Spectra of **23**

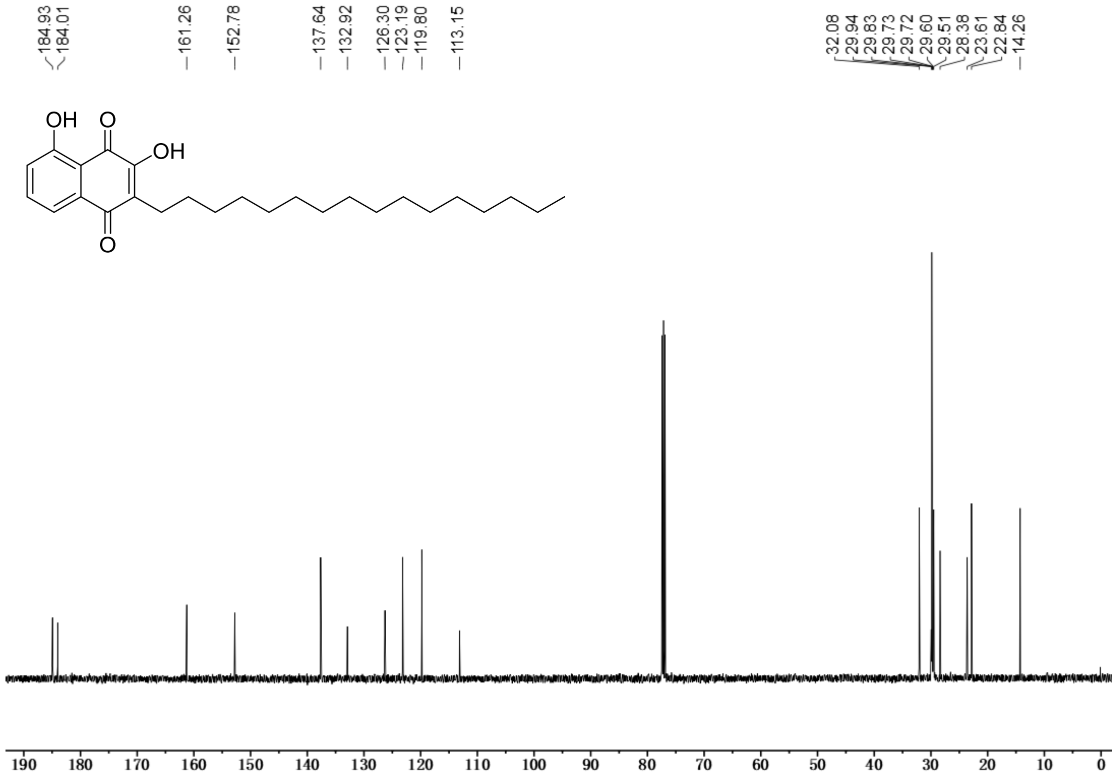

^{13}C NMR Spectra of **23**

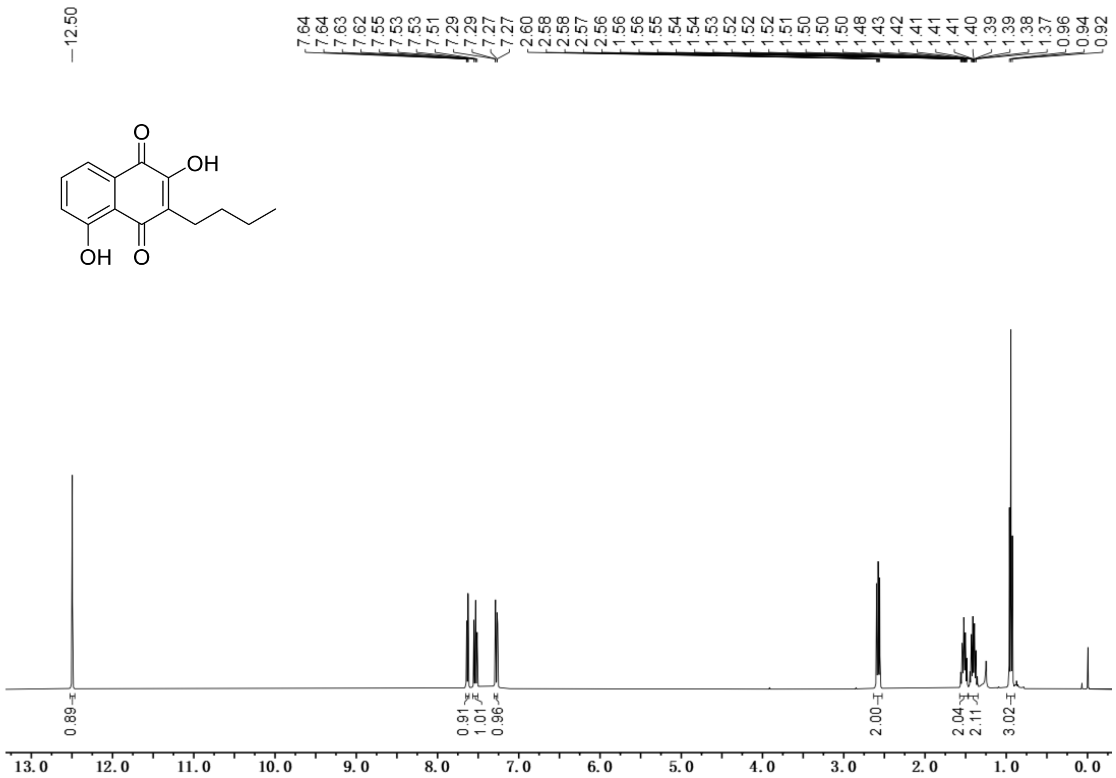

^{1}H NMR Spectra of **24**

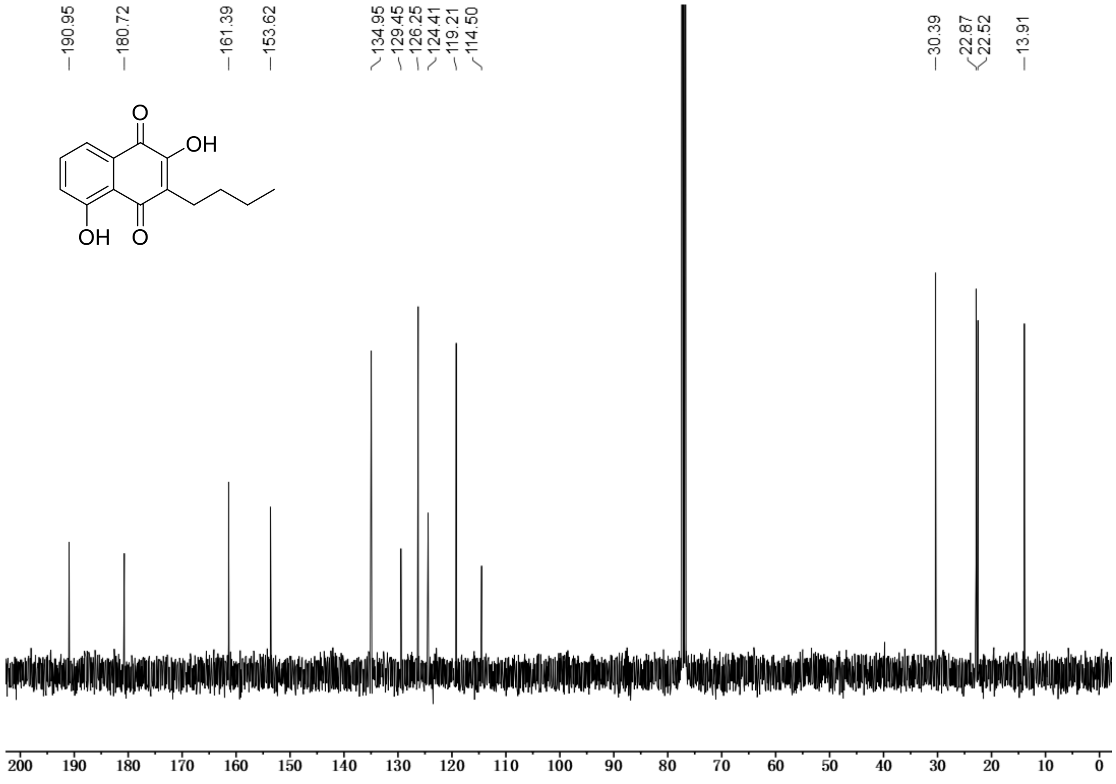

^{13}C NMR Spectra of **24**

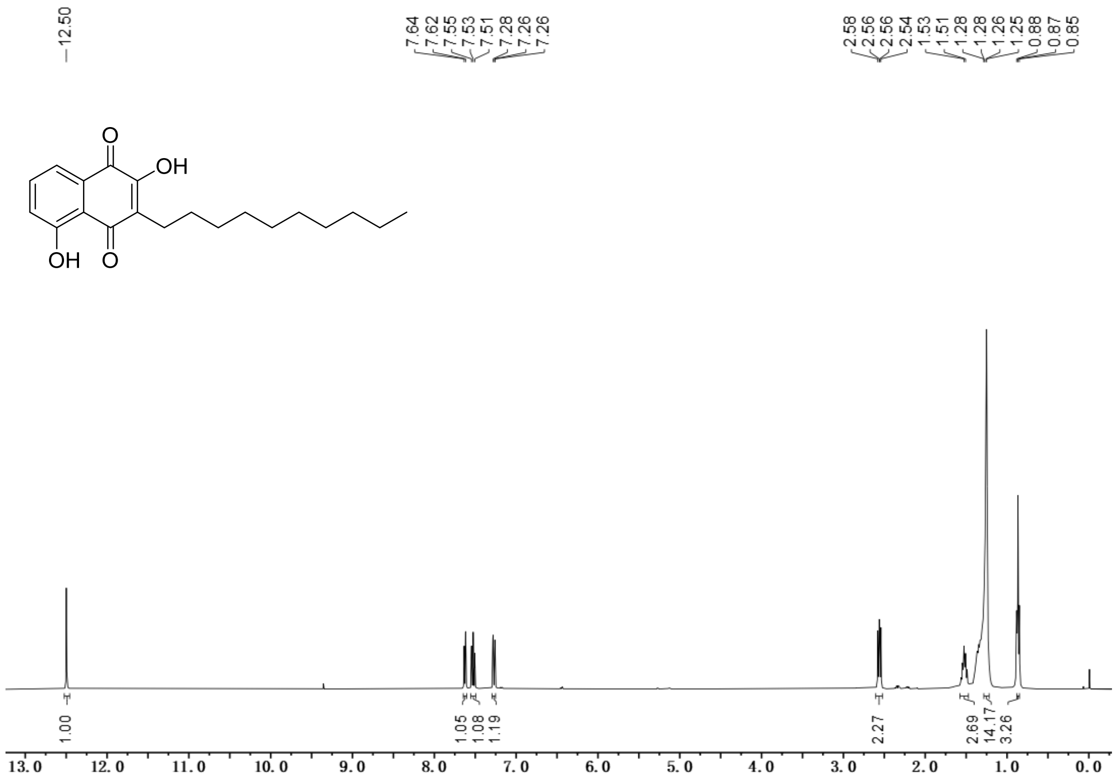

^{1}H NMR Spectra of **25**

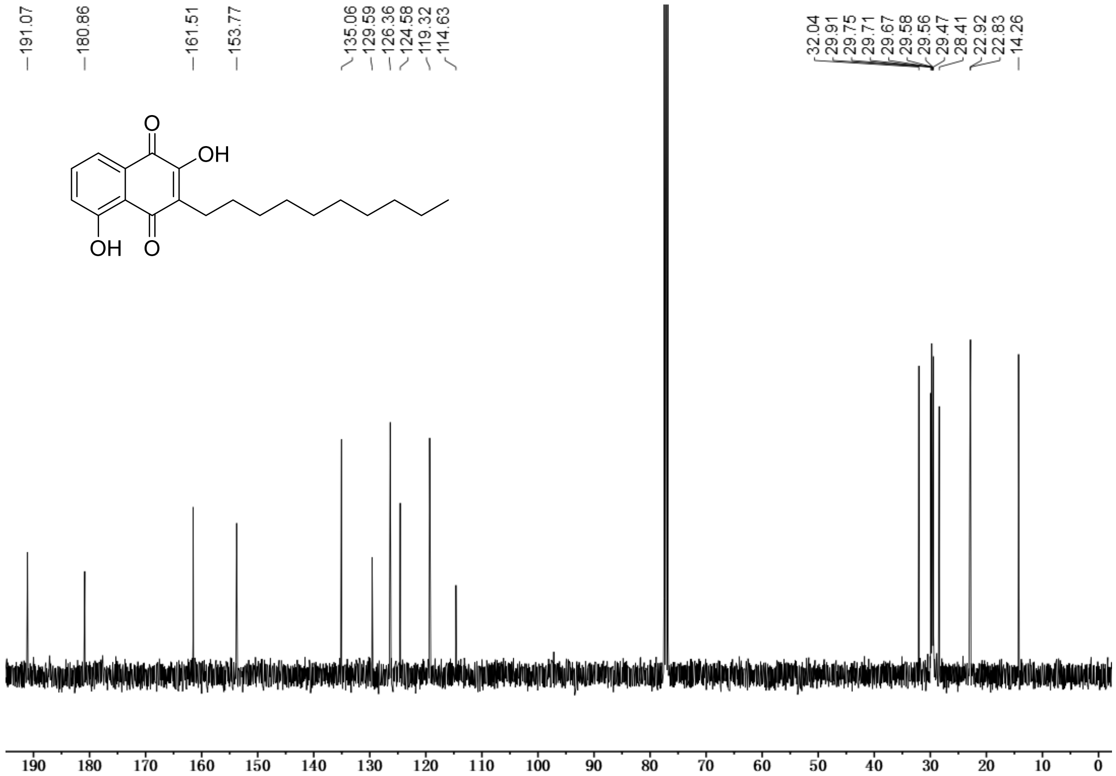

^{13}C NMR Spectra of **25**

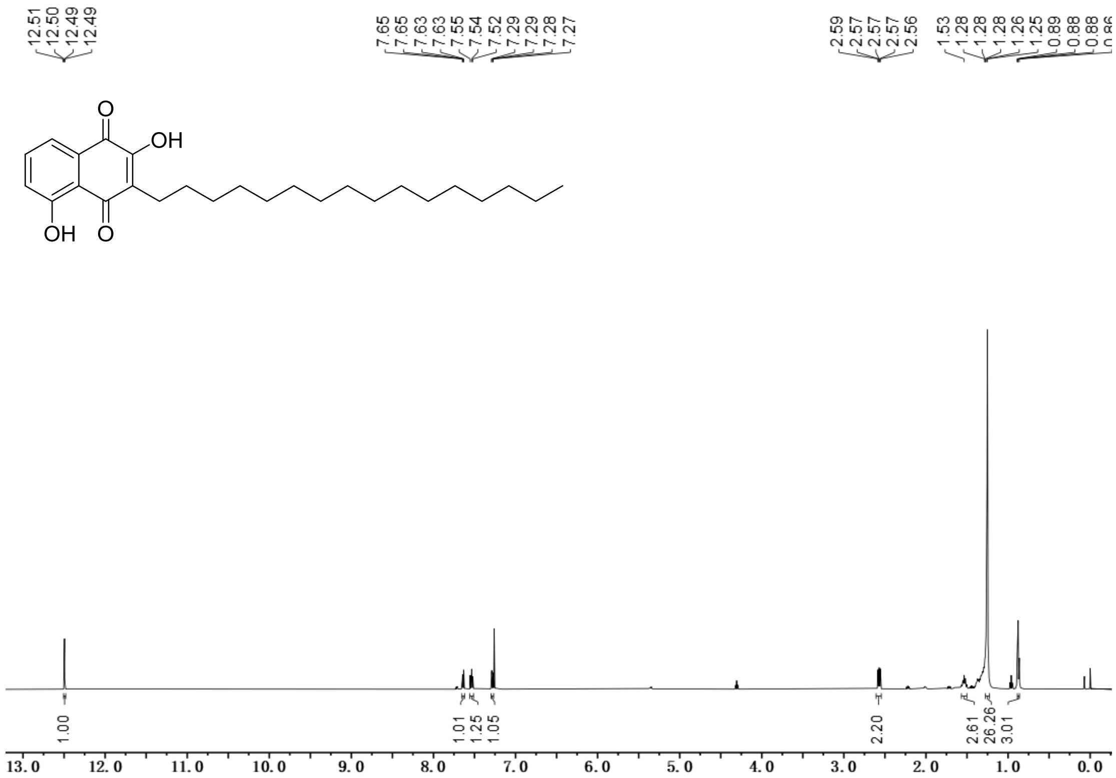

^{1}H NMR Spectra of **26**

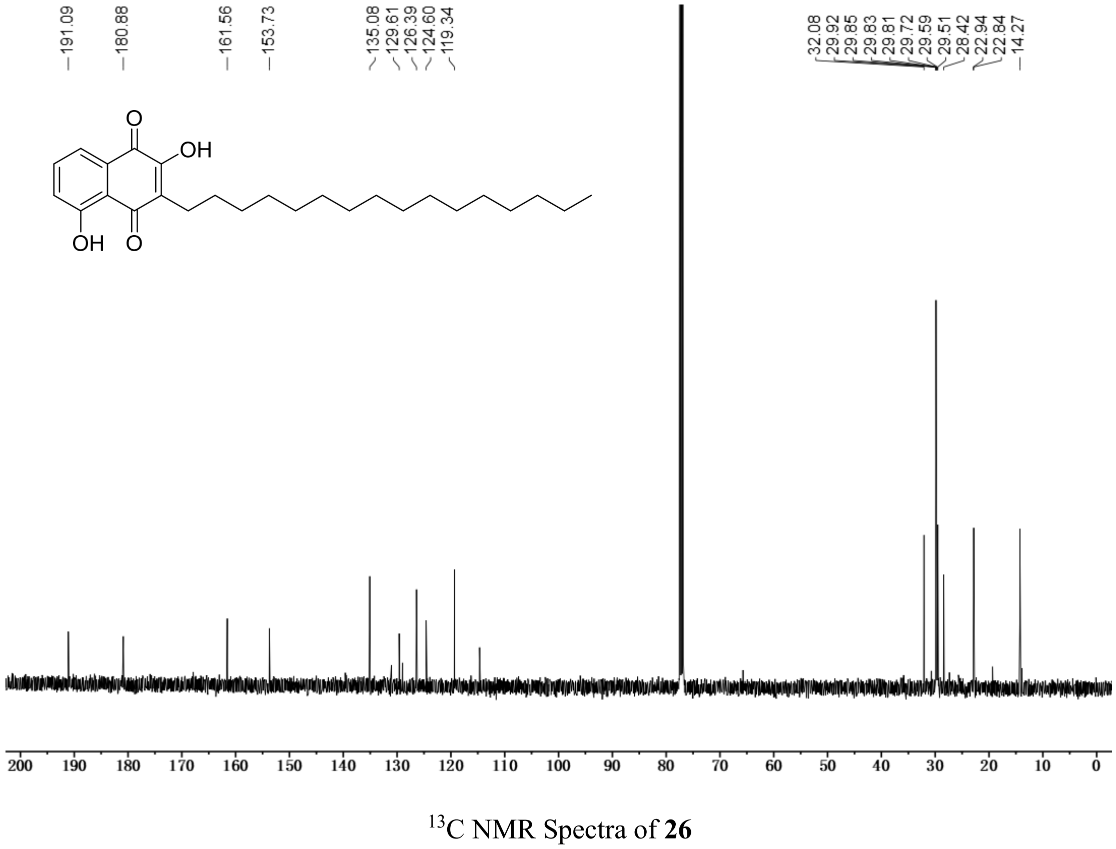

^{13}C NMR Spectra of **26**

Data S2. HPLC purity analysis of representative active compounds 4 and 11

Representative active compounds **4** and **11** were analyzed by analytical HPLC. Purity was calculated by peak-area normalization.

Figure S1. HPLC chromatogram of compound **4**.

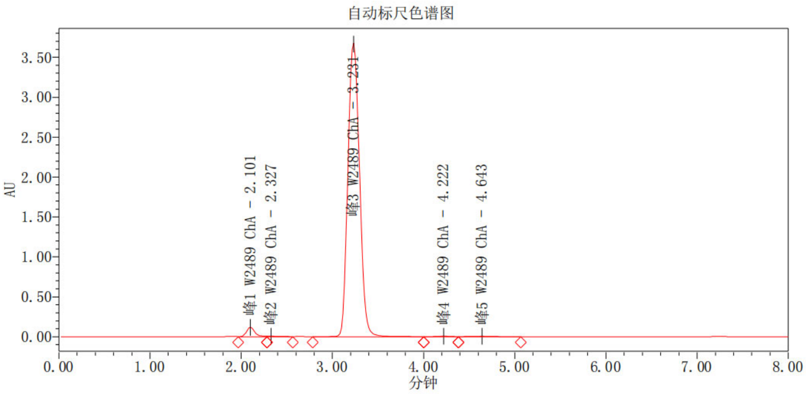

峰结果

	名称	保留时间 (分钟)	面积 (微伏*秒)	高度 (微伏)	含量	单位
1	峰1 W2489 ChA	2. 101	768777	118447		
2	峰2 W2489 ChA	2. 327	60857	6642		
3	峰3 W2489 ChA	3. 231	32540435	3676883		
4	峰4 W2489 ChA	4. 222	73398	6842		
5	峰5 W2489 ChA	4. 643	90874	6078		

The HPLC purity of compound 4 was 97.036% based on peak-area normalization.

Figure S2. HPLC chromatogram of compound **11**.

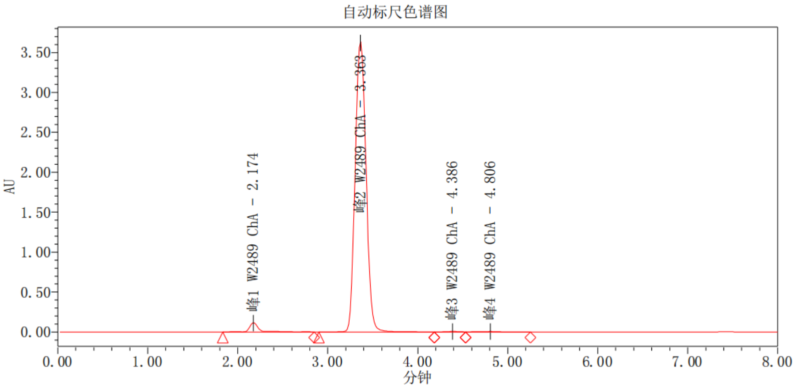

峰结果

	名称	保留时间 (分钟)	面积 (微伏*秒)	高度 (微伏)	含量	单位
1	峰1 W2489 ChA	2. 174	821730	112541		
2	峰2 W2489 ChA	3. 363	30700161	3636230		
3	峰3 W2489 ChA	4. 386	68741	6751		
4	峰4 W2489 ChA	4. 806	91953	6165		

The HPLC purity of compound **11** was 96.899% based on peak-area normalization.

Table S1. Raw cell viability data (%) of juglone and its derivatives in HaCaT cells after 24 h treatment at the indicated concentrations, determined by the CCK-8 assay. Data are expressed as individual technical replicate values from three parallel wells (n = 3).

**Compounds**	**Cell viability (%) at different concentrations (µg/mL)**						
	**200**	**100**	**50**	**25**	**12.5**	**6.25**	**0**
	6.639	8.501	9.587	40.161	86.788	90.014	93.703
**1**	7.323	8.044	10.425	41.669	94.064	98.173	99.8
	6.865	7.452	8.961	43.483	93.993	99.8	99.8
	72.219	79.465	87.885	91.270	98.815	99.298	99.800
**2**	67.271	69.353	86.741	98.673	99.757	99.298	99.800
	63.025	80.306	86.429	94.334	94.987	99.298	96.966
	0.843	2.573	2.996	4.524	6.757	11.095	99.800
**3**	0.876	2.663	3.150	4.359	6.628	9.418	99.533
	0.944	2.688	2.923	4.292	6.665	9.633	95.445
	29.631	63.575	86.075	96.230	95.622	98.628	99.800
**4**	40.618	51.416	80.442	82.551	93.135	98.628	94.406
	38.895	55.595	77.605	90.498	99.800	98.628	99.800
	8.983	10.393	11.520	12.186	22.151	49.597	96.297
**5**	8.809	9.749	10.252	10.374	23.798	54.460	98.470
	9.267	8.796	9.935	10.726	25.271	52.151	99.800
**6**	6.511	8.365	52.202	78.745	92.821	83.829	99.800

Compounds	Cell viability (%) at different concentrations (µg/mL)						
	**200**	**100**	**50**	**25**	**12.5**	**6.25**	**0**
	6.821	8.349	53.023	68.686	80.842	87.447	96.581
	6.883	8.757	55.449	66.206	86.150	87.657	99.800
	0.502	0.597	1.095	2.957	4.735	4.891	96.327
**7**	0.522	0.575	1.233	2.947	4.570	5.187	99.800
	0.516	0.523	1.076	2.837	4.495	5.339	99.800
	51.195	82.537	90.159	93.824	94.192	98.581	99.800
**8**	55.753	78.613	88.052	93.824	99.442	97.895	98.486
	54.732	68.468	84.946	84.629	97.510	99.211	99.135
	53.081	85.542	92.955	99.800	94.524	99.125	99.800
**9**	55.073	93.149	99.800	97.230	99.800	99.125	96.023
	53.095	89.434	99.475	96.646	99.800	99.125	99.800
	17.514	33.624	48.683	81.555	78.466	95.168	99.800
**10**	17.459	33.869	48.033	82.129	90.867	94.001	98.308
	17.076	34.556	41.399	80.169	85.995	93.536	99.800
	59.842	65.658	71.182	86.301	98.251	99.264	99.800
**11**	61.390	70.997	71.921	96.296	91.310	99.264	91.743
	59.062	66.581	70.132	99.800	99.800	99.264	99.800
	2.568	13.534	35.361	51.699	64.989	96.075	95.254
**12**	2.519	13.133	37.151	57.735	54.753	81.667	99.800
	2.413	13.645	40.926	53.691	63.071	90.384	99.800
	75.351	76.286	84.582	87.389	99.800	92.989	99.800
**13**	71.140	72.551	83.303	77.584	84.629	99.800	99.800
	72.900	71.564	75.252	98.505	98.723	99.800	92.232

Compounds	Cell viability (%) at different concentrations (μg/mL)						
	**200**	**100**	**50**	**25**	**12.5**	**6.25**	**0**
**14**	18.101	22.007	32.689	54.251	67.780	96.993	99.800
	18.287	22.713	31.744	48.533	65.646	99.132	93.859
	17.146	19.414	32.563	54.637	72.759	98.045	99.800
**15**	38.686	79.577	89.974	89.639	99.800	83.939	99.610
	47.303	84.416	91.130	89.215	89.975	96.207	99.800
	39.995	94.733	90.550	83.670	89.954	96.207	99.774
**16**	83.913	87.785	94.571	99.235	92.805	99.800	97.902
	85.273	92.573	99.800	99.235	99.800	99.800	99.800
	86.757	88.242	99.800	99.235	96.349	98.743	99.800
**17**	41.150	77.763	95.355	96.059	98.015	99.519	99.800
	43.207	74.394	96.771	99.800	96.434	99.519	99.800
	45.017	80.399	90.437	93.568	97.957	99.519	97.528
**18**	17.855	35.823	70.102	79.964	98.860	99.039	98.341
	17.272	36.102	72.716	79.963	92.203	99.039	99.800
	17.520	35.428	69.829	80.366	99.800	99.039	98.919
**19**	1.031	16.655	53.329	53.207	57.498	90.348	93.985
	1.145	18.731	48.435	62.139	70.851	99.800	99.800
	1.060	18.438	49.118	53.183	62.534	96.947	99.800
**20**	24.514	25.518	30.739	93.386	99.800	99.735	99.338
	21.799	23.216	29.864	80.034	97.264	99.735	99.800
	22.217	29.796	31.455	88.050	89.996	99.735	99.800
**21**	41.265	69.981	81.142	84.939	82.913	92.738	99.800
	37.807	73.736	78.241	80.537	92.347	81.894	99.800

Compounds	Cell viability (%) at different concentrations (µg/mL)						
	**200**	**100**	**50**	**25**	**12.5**	**6.25**	**0**
	42.021	68.732	87.581	93.979	92.608	83.628	98.699
	67.116	90.339	97.880	99.800	94.907	99.601	98.983
**22**	81.348	82.888	99.800	99.800	98.765	99.601	96.443
	74.902	76.927	99.130	94.085	99.800	99.601	99.800
	99.800	99.800	99.800	99.800	99.800	98.051	99.800
**23**	91.861	99.800	99.800	93.923	95.567	99.800	99.800
	99.800	94.243	95.482	99.531	99.800	99.800	99.800
	7.206	20.394	50.216	66.778	94.680	89.014	99.800
**24**	8.090	18.245	58.394	66.634	86.162	99.800	99.800
	7.939	18.126	59.331	73.353	93.570	99.800	90.319
	8.376	11.251	20.545	21.418	27.081	36.102	99.022
**25**	7.775	10.617	16.789	22.114	28.030	31.502	99.800
	8.849	11.955	16.489	22.056	25.183	35.631	99.800
	68.013	74.124	87.657	99.800	96.396	99.800	99.800
**26**	65.663	73.189	88.533	84.341	99.800	92.779	99.800
	63.467	83.606	99.800	99.800	99.800	99.800	96.787
	3.332	10.451	21.305	46.589	44.145	57.879	98.878
**JUG**	3.335	10.888	19.349	40.260	51.385	58.698	99.800
	3.403	10.994	21.360	38.770	53.410	58.865	99.800

Data are expressed as individual replicate values (n = 3)

.

Table S2. Raw nitric oxide (NO) production data of juglone and its derivatives in LPS-stimulated HaCaT cells. Data are expressed as individual technical replicate values from three parallel wells (n = 3).

Treatment	NO production (μM)		
Control	3.2	3.5	4.7
LPS	50.4	54.3	57.5
DEX	10.9	13.8	11.3
JUG	15.6	20.1	16.2
1	12.1	14.6	13.2
2	14.1	16.8	15
4	12	10.1	12.1
6	39.8	47.1	43
8	41.9	49.2	46.9
9	32	38.4	34.9
10	20.1	24.9	21.6
11	12.6	9.9	11.7
12	28.6	35.2	30.7
13	24.1	29.6	26.4
14	16.5	20.8	17.6
15	18.2	22.7	19.1
16	30.2	37.9	31.8
17	9.1	11.8	9.7
18	16.9	21.2	16.8
19	27.4	34.8	28.7

Treatment	NO production (μM)		
20	17.2	21.6	17.9
21	13	17.6	13.5
22	16.2	20.9	16.9
23	24.6	31.8	25.5
24	56.8	48.9	49.1
26	29.9	38.6	31.4

Data are expressed as individual replicate values (n = 3).

Table S3. Predicted ADMET properties of compound 11

Physicochemical properties

Parameter	Value
Volume	201.042
Density	1.015
nHA	4
nHD	1
nRot	1
TPSA (Å^2)	63.6
logS	-3.388
logD	1.62
pKa (acid)	6.548
pKa (base)	3.153

Medicinal chemistry

Parameter	Value
QED	0.753
Synthetic accessibility score	2.0
Fsp3	0.091
Lipinski rule	0 violations
PAINS alerts	1
ALARM NMR alerts	2

Absorption

Parameter	Value
Caco-2 permeability	-4.551
MDCK permeability	-4.595
P-gp inhibitor probability	0.178
P-gp substrate probability	0.007
HIA probability	0.958

Distribution

Parameter	Value
Plasma protein binding (%)	93.262
BBB penetration probability	0.001
Fraction unbound (Fu, %)	6.277

Metabolism

Parameter	Value
CYP1A2 inhibitor probability	1.000
CYP2D6 inhibitor probability	0.989
CYP3A4 inhibitor probability	0.650
HLM instability probability	0.975

Excretion

Parameter	Value
Plasma clearance (ml/min/kg)	0.923
Half-life (h)	1.949

Toxicity

Parameter	Value
hERG blocker probability	0.056
DILI probability	0.752
AMES mutagenicity probability	0.773
A549 cytotoxicity probability	0.084
